# Identification of Crucial Candidate Genes and Pathways in Glioblastoma Multiform by Bioinformatics Analysis

**DOI:** 10.3390/biom9050201

**Published:** 2019-05-24

**Authors:** Ali Mohamed Alshabi, Basavaraj Vastrad, Ibrahim Ahmed Shaikh, Chanabasayya Vastrad

**Affiliations:** 1 Department of Clinical Pharmacy, College of Pharmacy, Najran University, Najran 61441, Saudi Arabia; dr.aliresearch19@gmail.com; 2 Department of Pharmaceutics, SET`S College of Pharmacy, Dharwad, Karnataka 580002, India; basavarajmv@gmail.com; 3 Department of Pharmacology, College of Pharmacy, Najran University, Najran 61441, Saudi Arabia; i.ibrahimshaikh09@gmail.com; 4 Biostatistics and Bioinformatics, Chanabasava Nilaya, Bharthinagar, Dharwad 580001, Karnataka, India

**Keywords:** glioblastoma multiform, topology analysis, miRNA-target gene network, TF-target gene network, differential gene expression

## Abstract

The present study aimed to investigate the molecular mechanisms underlying glioblastoma multiform (GBM) and its biomarkers. The differentially expressed genes (DEGs) were diagnosed using the limma software package. The ToppGene (ToppFun) was used to perform pathway and Gene Ontology (GO) enrichment analysis of the DEGs. Protein-protein interaction (PPI) networks, extracted modules, miRNA-target genes regulatory network and TF-target genes regulatory network were used to obtain insight into the actions of DEGs. Survival analysis for DEGs was carried out. A total of 590 DEGs, including 243 up regulated and 347 down regulated genes, were diagnosed between scrambled shRNA expression and Lin7A knock down. The up-regulated genes were enriched in ribosome, mitochondrial translation termination, translation, and peptide biosynthetic process. The down-regulated genes were enriched in focal adhesion, VEGFR3 signaling in lymphatic endothelium, extracellular matrix organization, and extracellular matrix. The current study screened the genes in the PPI network, extracted modules, miRNA-target genes regulatory network, and TF-target genes regulatory network with higher degrees as hub genes, which included *NPM1, CUL4A, YIPF1, SHC1, AKT1, VLDLR, RPL14, P3H2, DTNA, FAM126B, RPL34*, and *MYL5*. Survival analysis indicated that the high expression of *RPL36A* and *MRPL35* were predicting longer survival of GBM, while high expression of *AP1S1* and *AKAP12* were predicting shorter survival of GBM. High expression of *RPL36A* and *AP1S1* were associated with pathogenesis of GBM, while low expression of *ALPL* was associated with pathogenesis of GBM. In conclusion, the current study diagnosed DEGs between scrambled shRNA expression and Lin7A knock down samples, which could improve our understanding of the molecular mechanisms in the progression of GBM, and these crucial as well as new diagnostic markers might be used as therapeutic targets for GBM.

## 1. Introduction

Glioblastoma multiform (GBM) is a malignancy that occurs in the astrocytes and it is the most common type of central nervous system cancer in adults worldwide [1]. In recent decades, the mortality caused by glioblastoma has diminished dramatically owing to the great development in early identification and treatment [2]. However, glioblastoma debris is an outstanding global health problem that may be associated to the absence of exhaustive systemic and sympathetic underlying molecular mechanisms of tumorigenesis. The metastasis and recurrence of GBM as well as its indigent prognosis, results in indigent survival for patients [3]. Thus, an enhanced interpretation of the elemental molecular mechanisms and gene networks associated in the development and advancement of GBM is required.

Various studies on the mechanisms and therapeutic actions for the treatment of GBM have been disclosed to date [4]. Exposure to prior radiation, cigarette smoking, alcohol consumption, use of drugs of any kind, or dietary intake of cured or smoked meat or fish, is considered to be a significant risk factor for the development of GBM [5]. During cancer development, multiple biomarkers experience upor down regulation [6]. A number of biomarkers and signaling pathways linked in the advancement of GBM have already been diagnosed [7]. Graff et al. (2005) [8] reported that protein kinase Cβ (PKCβ) was an effective activator of angiogenesis and cancer growth in carcinomas of the brain. In addition, the authors noticed that the expression of SIRT2 was down regulated in patients with GBM [9]. Previous studies have established that expression of vascular endothelial growth factor (VGEF) is responsible for progression of GBM [10]. Additionally, epidermal growth factor receptor (EGFR), which participates in signaling pathways associated with various tumors such as lung cancer [11], colorectal cancer [12], head and neck cancer [13], and breast cancer [14], and this gene has been previously noticed to be over expressed in glioblastoma [15]. The tumor suppressor gene Lin7A decreases the invasion of GBM cells [16]. Lin7A knockdown may assist in advancement of GBM. The aim of our study is to know if the knockdown of Lin7A is responsible for invasion of GBM cells and also find out how it affects or alters the expression of other genes. In this study we identified differential expressed genes (DEGs) between scrambled shRNA expression and Lin7A knock down samples by various bioinformatics methods.

In the present study, we utilized the conception of this high-throughput method. Therefore, we examined the hub node recognition using the statistical methods and comparative analysis among the list of data compiled from numerous sources. We analyzed the differential expressed genes (DEGs) between scrambled shRNA expression and Lin7A knock down samples, in order to investigate the underlying molecular mechanisms of GBM. Afterwards, pathway and gene enrichment analysis were executed. Protein-protein interaction (PPI) networks and its modules were constructed and topological parameters such as degree, betweenness centrality, closeness centrality, stress centrality and clustering coefficient, in order to exercise and analyze target genes for the diagnosis and treatment of GBM. In addition, target gene-miRNA networks and target gene-TFs networks were constructed and analyzed. In addition, our study concentrated on the present developments around the GBM and their important differences underlined between the subgroups. Moreover, the heterogeneity of GBM has not been treated sufficiently in clinical trials, which is one of the enigmatic reasons for the challengein advancing the novel therapeutic approaches. This study aimed to diagnose protein-coding genes that can be used as molecular markers of early stage GBM.

## 2. Materials and Methods

### 2.1. Illumina Microarray Data

The microarray expression profile dataset E-MTAB-5871, which is based on the HumanHT-12 v4 Expression BeadChip (Illumina Way, San Diego, CA, USA), was downloaded from the European Bioinformatics Institute ArrayExpress database (https://www.ebi.ac.uk/arrayexpress/experiments/E-MTAB-5871/). The dataset contained 9 samples, including 3 scrambled shRNA expression samples, 3 Lin7A knock down samples, and 3 Lin7A knock down complemented by wild type Lin7A samples. In the current study, the scrambled shRNA expression, Lin7A knock down samples, and Lin7A knock down complemented by wild type Lin7A samples were analyzed by bioinformatics methods.

### 2.2. Data Preprocessing and Differential Expression Analysis

The authentic array data were transformed into expression measures. Background correction, data normalization, and probe summarization were performed using the quantile [17] algorithm in the R lumi package (https://www.bioconductor.org/packages/release/bioc/html/lumi.html) [18]. Paired test based on the Linear Models for Microarray Data package (https://bioconductor.org/packages/release/bioc/html/limma.html) [19] in R (https://www.rproject.org/) was used to identify DEGs between scrambled shRNA expression and Lin7A knock down samples. Multiple testing corrections were executed with the Benjamini-Hochberg method [20] to gain the adjusted *p*-value. Subsequently, log2fold change (log2FC) was determined. Only those genes indicating |log2FC| > 1.0 and adjusted *p* < 0.001 were regarded as DEGs.

### 2.3. Pathway Enrichment Analysis

By using online based software ToppGene (ToppFun) (https://toppgene.cchmc.org/enrichment.jsp) [21], which integrates different pathway databases such as Kyoto Encyclopedia of Genes and Genomes (KEGG; http://www.genome.jp/kegg/) [22], Pathway Interaction Database (PID, http://pid.nci.nih.gov/) [23], Reactome (https://reactome.org/PathwayBrowser/) [24], Molecular signatures database (MSigDB, http://software.broadinstitute.org/gsea/msigdb/) [25], GenMAPP (http://www.genmapp.org/) [26], Pathway Ontology (https://bioportal.bioontology.org/ontologies/PW) [27] and PantherDB (http://www.pantherdb.org/) [28]. In order to analyze the identified DEGs at the functional level, KEGG, PID, Reactome, MSigDB and PantherDB pathway analysis were performed using the ToppGene (ToppFun) online tool. *p* < 0.05 was set as the threshold value.

### 2.4. GO Term Enrichment Analysis

Gene Ontology (GO; http://geneontology.org/) [29] is a tool for consolidation of biology that compiles structured, defined, and disciplined glossary for huge scale gene annotation. The ToppGene (ToppFun) involves an extensive set of functional annotation tools that have been advanced for associating functional terms with lists of genes via clustering algorithms. In order to analyze the identified DEGs at the functional level, GO enrichment was performed using the ToppGene (ToppFun) online tool. *p* < 0.05 was set as the threshold value.

### 2.5. PPI Network Construction

Biomolecular Interaction Network Database (BIND, http://www.bind.ca/) [30], Human Protein Reference Database (HPRD, http://www.hprd.org/) [31], General Repository for Interaction Datasets (BioGRID, https://thebiogrid.org/) [32], The comprehensive resource of mammalian protein complexes (CORUM, http://mips.helmholtz-muenchen.de/corum/) [33], Database of Interacting Proteins (DIP, http://dip.doe-mbi.ucla.edu) [34], The International Molecular Exchange Consortium (IntAct, http://www.imexconsortium.org) [35], The Molecular INTeraction Database (MINT, http://mint.bio.uniroma2.it/mint/) [36], the Munich Information Center for Protein Sequences (MIPS) protein interaction resource on yeast (MPact, http://mips.gsf.de/genre/proj/mpact) [37], Mammalian Protein-Protein Interaction Database (MPPI, http://mips.gsf.de/proj/ppi/) [38], and The Online Predicted Human Interaction Database (OPHID, http://ophid.utoronto.ca) [39] are a precompiled global resource designed to evaluate PPI information. In the current study, the iRefIndex (http://irefindex.org/wiki/index.php?title=iRefIndex) [40] online tool was used to generate the graph file for the PPI network of DEGs, and those experimentally validated interactions with a combined score >0.4 were selected as significant.

The bulk of the PPI networks in the biological network constructed were noticed to obey topological properties [41]. Thus, the degree of connectivity, betweenness centrality, stress, closeness centrality, and clustering coefficient were statistically analyzed in networks using the cytoscape version 3.6.0 (www.cytoscape.org/) [42], to obtain the significant nodes or hub proteins [43] in the PPI networks. Subsequently, the overlapping target genes were identified and the miRNA-target gene pairs.

### 2.6. Module Analysis

Interaction reliability assessment and weighted clustering coefficient (PEWCC1) explain the densely connected nodes from the large protein-protein interaction (PPI) network, which can be called as modules [44]. The PEWCC1 algorithm used in the module construction limits the existence of a single node in more than one module. Further, if a single hub is interacting with more than one module with huge interactions, the node is attributed to be a super hub, which can physically allow us to restrict it as a crosstalk among the modules. The modules obtained were used for further analysis after refinement using degree of connectivity.

### 2.7. Construction of the Target Gene-miRNA Network

miRNet (http://www.mirnet.ca) [45] is an online tool for target gene-miRNA network generation, which integrates 10 miRNA database such as TarBase (http://diana.imis.athena-innovation.gr/DianaTools/index.php?r=tarbase/index) [46], miRTarBase (http://mirtarbase.mbc.nctu.edu.tw/php/download.php) [47], miRecords (http://miRecords.umn.edu/miRecords) [48], miR2Disease (http://www.mir2disease.org/) [49], the Human microRNA Disease Database HMDD (http://www.cuilab.cn/hmdd) [50], PhenomiR (http://mips.helmholtz-muenchen.de/phenomir/) [51], SM2miR (http://bioinfo.hrbmu.edu.cn/SM2miR/) [52], PharmacomiR (http://www.pharmaco-mir.org/) [53], EpimiR (http://bioinfo.hrbmu.edu.cn/EpimiR/) [54], and starBase (http://starbase.sysu.edu.cn/) [55]. The targategene-miRNA network was visualized using the Cytoscape version 3.6.0.

### 2.8. Construction of the Target Gene-TF-Network

NetworkAnalyst (https://www.networkanalyst.ca/) [56] is online tool for target gene-TF network generation, which integrates the ENCODE ChIP-seqTF database (https://www.encodeproject.org/) [57], which provided data on eukaryotic transcription factors, consensus binding sequences (positional weight matrices), experimentally confirmed binding sites, and regulated genes. The target gene-TF network was visualized using Cytoscape version 3.6.0 software.

### 2.9. Survival Analysis of Hub Genes

Gene expression profiling and interactive analyses (GEPIA, http://gepia.cancer-pku.cn/) [58] is a web-based database for exploring correlations between gene expression and cancer patient survival. The survival analysis of core genes was conducted by clinical data of GBM patients obtained from the TCGA and GTEx data portal, including the overall survival (OS) time and vital status. The patients were divided into the following two groups according to the median expression value of the diagnosed overlapping hub genes in the cancer group: High expression group and low expression group. The Kaplan–Meier curves of the two groups are drawn. *p*-value, 0.05 is set as a statistically significant threshold. Based on the GEPIA on the webpage, the hazard ratio (HR) with 95% confidence intervals and log rank *p*-values were calculated and the curves were generated.

### 2.10. The mRNA Expression Levels of Hub Genes in GBM

The expression level of all the prognosis-related genes identified by Kaplan–Meier analysis and Cox proportional hazard analysis was performed in boxplots to visualize the relationship between GBM and healthy tissue based on data from TCGA and GTEx. The expression level was expressed as mean (standard deviation). A *p*-value of 0.01 is considered statistically significant.

### 2.11. Mutation Analysis

The online tool CBio Cancer Genomics Portal (http://www.cbioportal.org) [59] is an open strong platform that is useful for visualization, analysis, and downloadinga large-scale cancer genomic dataset of GBM. This online tool is useful for investigators to find and compare genetic modifications across samples.

## 3. Results

### 3.1. Identification of DEGs

**Figure 1:**
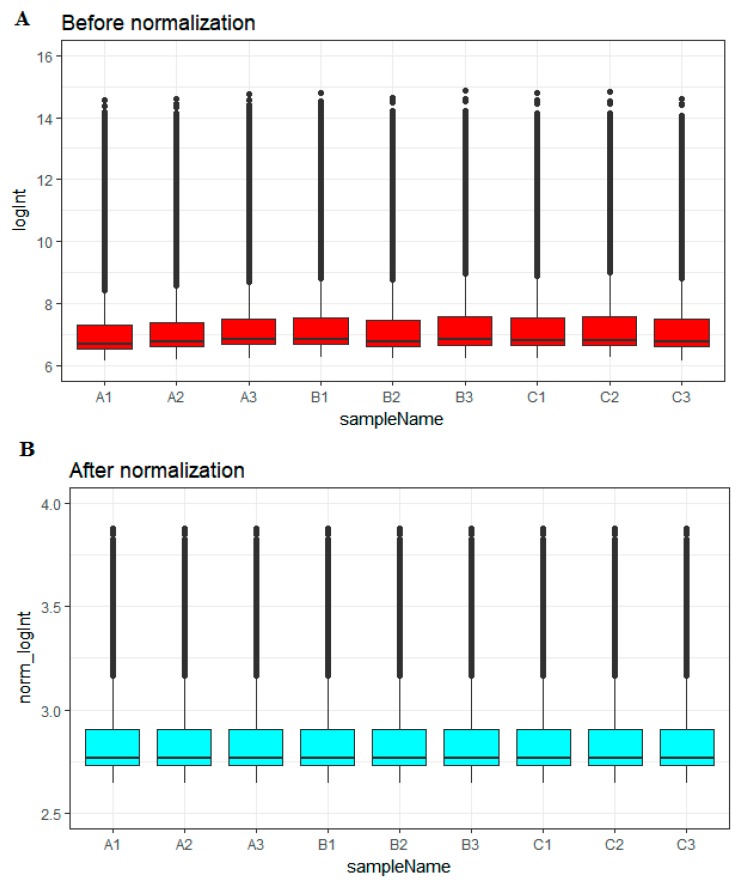
Box plots of the gene expression data before (**A**) and after normalization (**B**). The horizontal axis represents the sample symbol and the vertical axis represents the gene expression values. The black line in the box plot represents the median value of gene expression. (A1, A2, A3 = scrambled shRNA expression samples; B1, B2, B3 = Lin7A knock down samples; C1, C2, C3 = Lin7A knock down complemented by wild type Lin7A samples).

Before normalization, the medians of gene expression in each sample were notably recognizable (Figure 1A). However, the medians became persistent and were at a similar level following normalization (Figure 1B), advising us that the normalization process was fruitful and the normalized data may be used for additional analysis.

**Figure 2:**
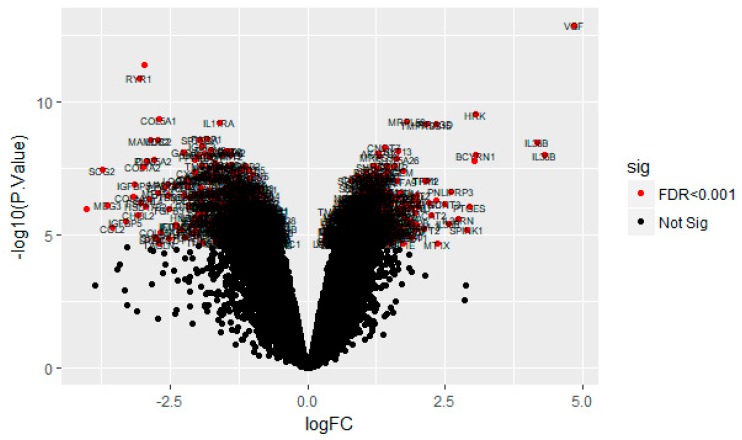
Volcano plot of differentially expressed genes. Genes with a significant change of more than two-fold were selected.

**Figure 3:**
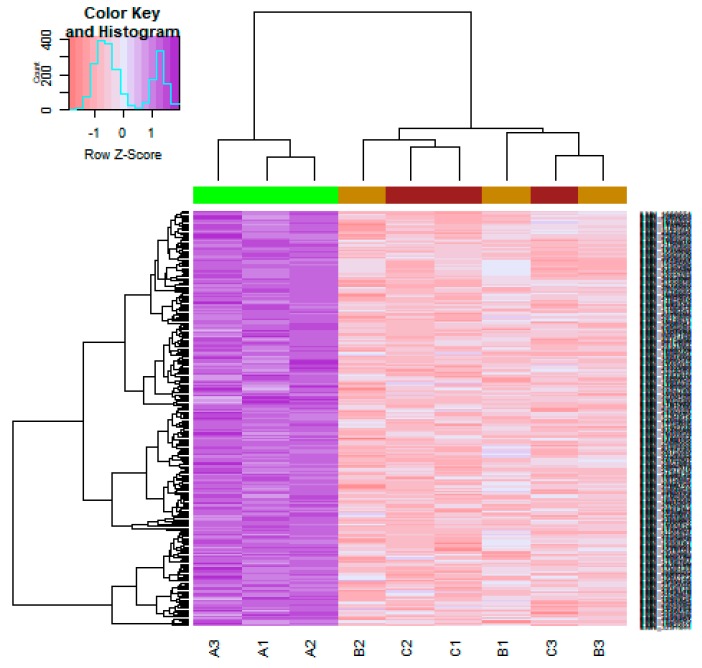
Heat map of up regulated differentially expressed genes. Legend on the top left indicates the log fold change of the genes. (A1, A2, A3 = scrambled shRNA expression samples; B1, B2, B3 = Lin7A knock down samples; C1, C2, C3 = Lin7A knock down complemented by wild type Lin7A samples).

**Figure 4:**
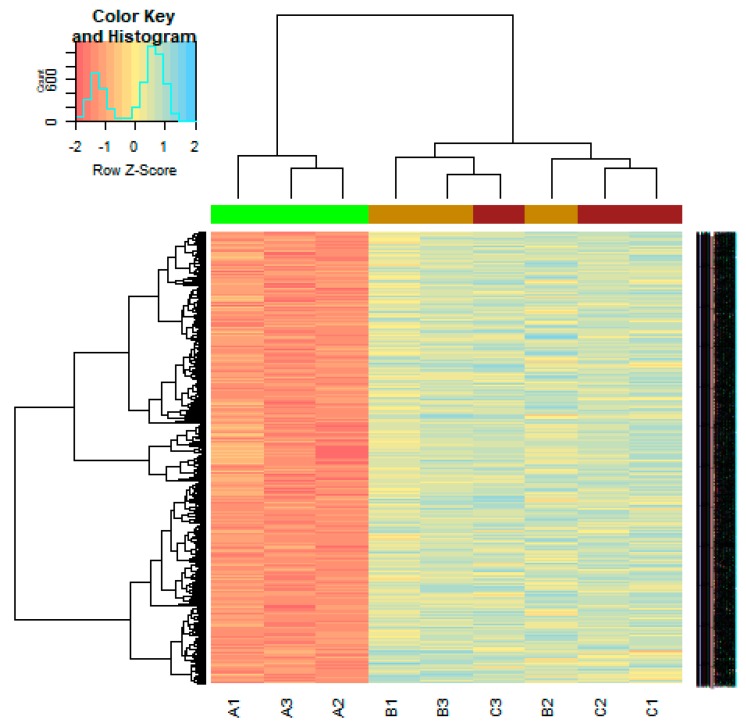
Heat map of down regulated differentially expressed genes. Legend on the top left indicates the log fold change of the genes. (A1, A2, A3 = scrambled shRNA expression samples; B1, B2, B3 = Lin7A knock down samples; C1, C2, C3 = Lin7A knock down complemented by wild type Lin7A samples).

On the basis of the threshold criteria, a total of 590 DEGs were obtained, including 243 up regulated and 347 down regulated genes in scrambled shRNA expression and Lin7A knock down samples (Table S1). There were 590 common DEGs across the two treatment groups. A volcano plot illuminated the expression variance in the number of DEGs at different *p*-values and log fold changes (Figure 2). A heat map revealed that the up and down regulated DEGs in the scrambled shRNA expression and in the Lin7A knock down samples (Figure 3 and Figure 4).

### 3.2. Pathway Enrichment Analysis

The up-regulated genes were enriched in pathways from different pathway databases such as ribosome, mineral absorption, mitochondrial translation termination, mitochondrial translation, pyruvate metabolism, and oxidative phosphorylation, which are listed Table S2, meanwhile down-regulated genes were enriched in pathways from different pathway databases such as focal adhesion, protein digestion, absorption, VEGFR3 signaling in lymphatic endothelium, IL4-mediated signaling events, collagen biosynthesis, modifying enzymes, collagen formation, ensemble of genes encoding extracellular matrix, extracellular matrix-associated proteins, ensemble of genes encoding core extracellular matrix including ECM glycoproteins, collagens, proteoglycans, integrin signaling pathway, and interleukin signaling pathway, as shown in Table S3.

### 3.3. GO Term Enrichment Analysis

GO analysis results showed that up-regulated genes were significantly enriched in all GO terms (BP, CC and MF) such as translation, peptide biosynthetic process, ribosome, ribosomal subunit, structural constituent of ribosome and RNA binding, as listed in Table S4, while, down-regulated DEGs were significantly enriched in all GO terms (BP, CC and MF) such as extracellular matrix organization, extracellular structure organization, extracellular matrix, extracellular matrix component, platelet-derived growth factor binding, and growth factor binding, as listed in Table S5.

### 3.4. Construction PPI Network and Topological Analysis

**Figure 5:**
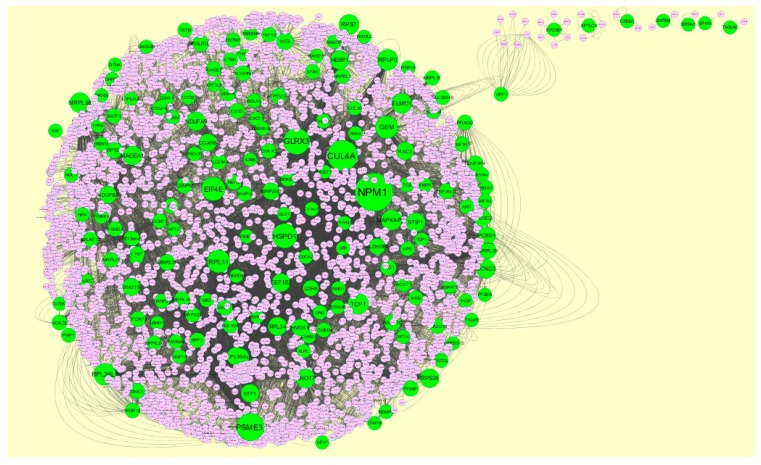
Protein–protein interaction network of differentially expressed genes (DEGs). Green nodes denotes up regulated genes.

**Figure 6:**
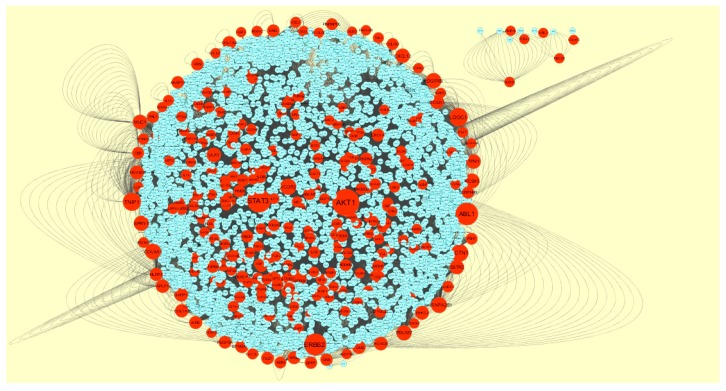
Protein–protein interaction network of differentially expressed genes (DEGs). Red nodes denotes down regulated genes.

**Figure 7:**
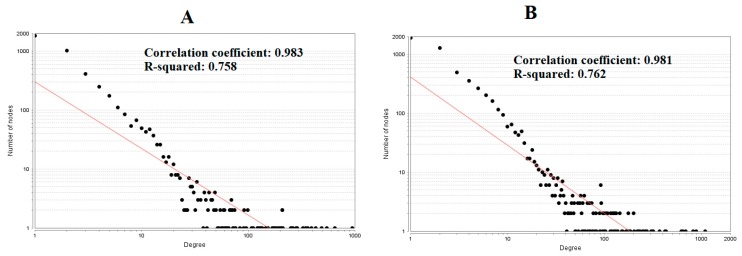
Node degree distribution (**A**—up regulated genes; **B**—down regulated genes).

**Figure 8:**
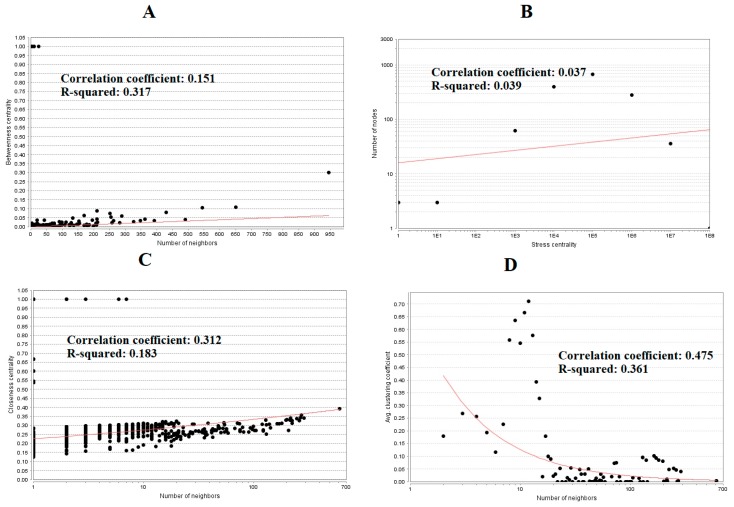
Regression diagrams for up regulated genes (**A**—betweenness centrality; **B**—stress centrality; **C**—closeness centrality; **D**—clustering coefficient).

**Figure 9:**
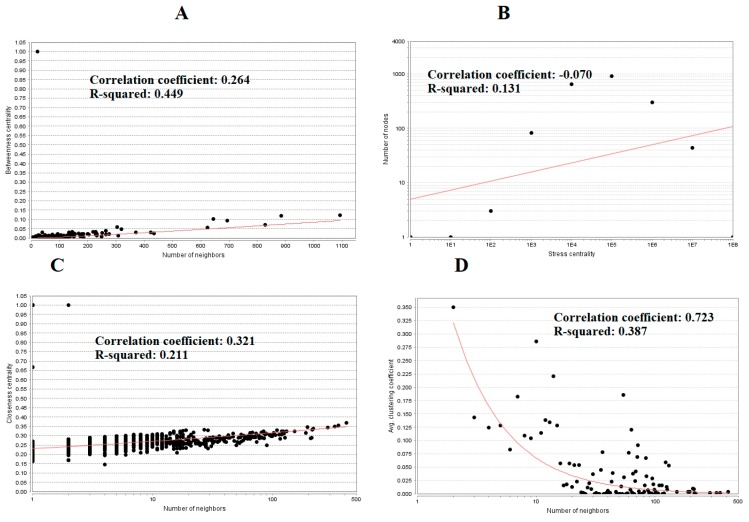
Regression diagrams for down regulated genes (**A**—betweenness centrality; **B**—stress centrality; **C**—closeness centrality; **D**—clustering coefficient).

The PPI network (up regulated) had 4437 nodes and 14,519 interactions, as shown in Figure 5 and are listed in Table S6. Similarly, The PPI network (down regulated) had 4683 nodes and 21,058 interactions, as shown in Figure 6 and are listed in Table S6. Hub genes (up regulated) with a high node degree [60] such as *NPM1*, *CUL4A*, *PSME3, GLRX3*, and *HSPD1* are listed in Table S6. R square = 0.758 and correlation coefficient = 0.983 for node degree (Figure 7A). Hub genes (up regulated) with high betweenness centrality [61] such as *NPM1*, *CUL4A*, *PSME3*, *HEBP1*, and *HSPD1* are listed in Table S6. R square = 0.317 and correlation coefficient = 0.151 for betweenness (Figure 8A). Hub genes (up regulated) with high stress genes [62] such as *NPM1*, *HSPD1*, *CENPM*, *RAC2*, and *CUL4A* are listed in Table S6. R square = 0.039 and correlation coefficient = 0.037 for stress (Figure 8B). Hub genes (up regulated) with high closeness centrality [63] such as *NPM1*, *CUL4A*, *PSME3*, *RPL14*, and *HSPD1* are listed in Table S6. R square = 0.183 and correlation coefficient = 0.312 for closeness (Figure 8C). Hub genes (up regulated) with low clustering coefficient [64] such as *YIPF1*, *EFCAB7*, *SPTLC3*, *CPEB1*, and *ZNF586* are listed in Table S6. R square = 0.361 and correlation coefficient = 0.475 for clustering coefficient (Figure 8D). Meanwhile, hub genes (down regulated) with a high node degree such as *SHC1*, *AKT1*, *STAT3*, *ABL1*, and *ERBB2*. R square = 0.762 and correlation coefficient = 0.981 for node degree (Figure 7B). Hub genes (down regulated) with high betweenness centrality such as *SHC1*, *AKT1*, *ABL1*, *STAT3*, and *HNRNPA2B1* are listed in Table S6. R square = 0.449 and correlation coefficient = 0.264 for betweenness (Figure 9A). Hub genes (down regulated) with high stress such as *AKT1*, *ABL1*), *STAT3*, *SHC1*, and *SSTR2* are listed in Table S6. R square = 0.131 and correlation coefficient = −0.070 for stress (Figure 9B). Hub genes (down regulated) with high closeness centrality such as *SHC1*, *AKT1*, *ABL1*, *ERBB2*, and *STAT3* are listed in Table S6. R square = 0.211 and correlation coefficient = 0.321 for closeness (Figure 9C). Hub genes (down regulated) with low clustering coefficient such as *VLDLR*, *FIBCD1*, FILIP1L, *UBL3*, and *TCEA3* are listed in Table S6. R square = 0.387 and correlation coefficient = 0.723 for the clustering coefficient (Figure 9D).

### 3.5. Module Analysis

**Figure 10:**
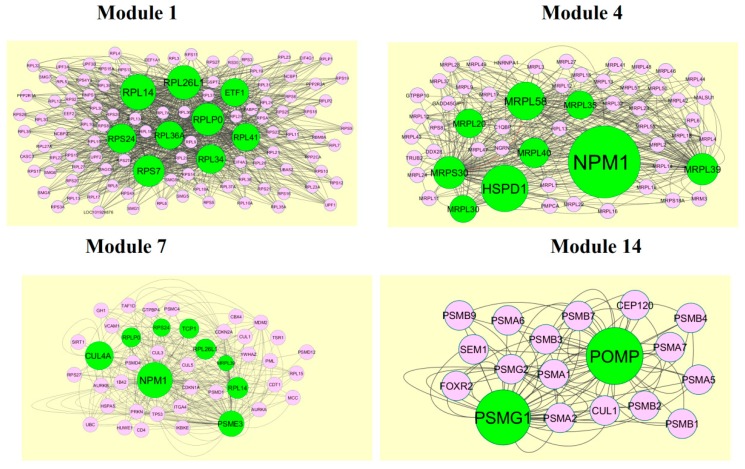
Modules in the protein-protein interaction (PPI) network. The green nodes denote the up-regulated genes.

A total of 1473 modules were extracted from the PPI network (up regulated). Module 1, module 4, module 7, and module 14 were significant modules in the PPI network (Figure 10). Module 1 had 108 nodes and 1002 edges. Hub genes such as *RPL14*, *RPL26L1*, *ETF1*, *RPS24*, *RPL36A*, *RPLP0*, *RPL41*, *RPL34*, and RPS7 were involved in module 1. Module 4 had 43 nodes and 262 edges. Hub genes such as *NPM1*, *MRPL39*, *MRPL35*, *MRPL58*, *MRPL20*, *MRPL40*, *MRPS30*, *HSPD1*, and *MRPL30* were involved in module 4. Module 7 had 43 nodes and 262 edges. Hub genes such as *PSME3*, *CUL4A*, *TCP1*, *RPLP0*, *RPS24*, *RPL26L1*, *MRPL39*, and *RPL14* were involved in module 7. Module 14 had 18 nodes and 82 edges. Hub genes such as *PSMG1* and *POMP* were involved in module 14.

**Figure 11:**
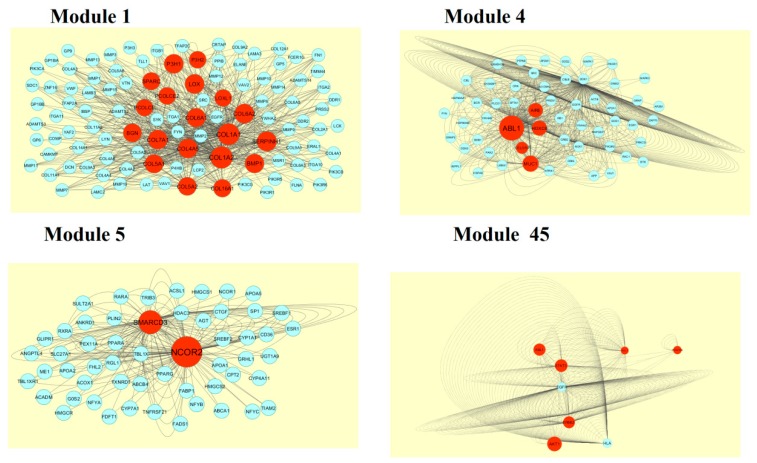
Modules in the PPI network. The red nodes denote the down-regulated genes.

A total of 4198 modules were extracted from PPI network (down regulated). Module 1, module 4, module 5, and module 45 were significant modules in this PPI network (Figure 11). Module 1 had 108 nodes and 622 edges. Hub genes such as *P3H2*, *P3H1*, *LOX*, *PCOLCE2*, *SPARC*, *PCOLCE*, *COL6A1*, *COL6A2*, *COL1A1*, *COL1A2*, *COL4A5*, *COL7A1*, *BGN*, *COL5A1*, *COL5A2*, *COL16A1*, *BMP1*, and *SERPINH1* were involved in module 1. Module 4 had 57 nodes and 553 edges. Hub genes such as *ABL1*, *CELSR3*, *MUC1*, *HOXC8*, and *AIRE* were involved in module 4. Module 5 had 55 nodes and 182 edges. Hub genes of such as *NCOR2* and *SMARCD3* were involved in module 5. Module 45 had 9 nodes and 346 edges. Hub genes such as *AKT1*, *ERBB2*, *ABL1*, *STAT3*, *MUC1*, and *PDGFRB* were involved in module 45.

### 3.6. Construction of the Target Gene-miRNA Network

**Figure 12:**
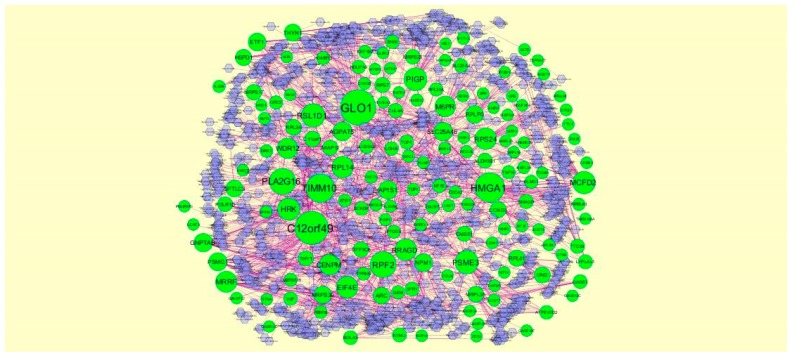
The network of up-regulated DEGs and their related miRNAs. The green circles nodes are the up-regulated DEGs, and purple diamond nodes are the miRNAs.

**Figure 13:**
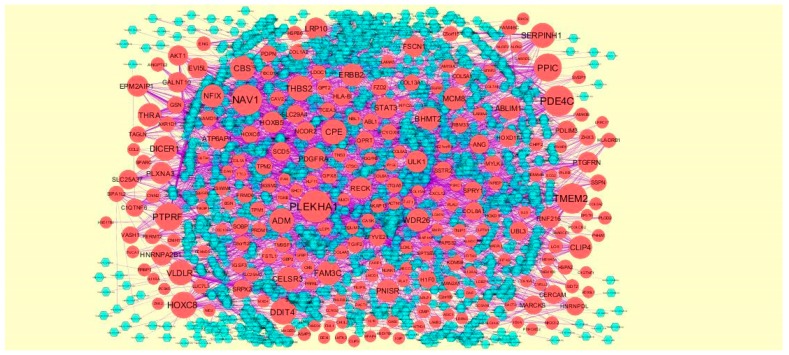
The network of down-regulated DEGs and their related miRNAs. The red circle nodes are the down-regulated DEGs, and blue diamond nodes are the miRNAs.

MicroRNA (miRNA) expression is responsible for development of cancer progression [65]. The miRNAs that may control the DEGs were diagnosed based on the up and down regulation expressions (Figure 12 and Figure 13). Top five up regulated targeted genes such as DTNA interacts with 182 miRNAs, *CNOT7* interacts with 182 miRNAs, *PSME3* interacts with 163 miRNAs, *CPEB1* interacts with 158 miRNAs, and EIF4E interacts with 157 miRNAs, are listed in Table S7. Meanwhile, top five down regulated targeted genes such as *FAM126B* interacts with 289 miRNAs, *WDR26* interacts with 283 miRNAs, *PRDM1* interacts with 235 miRNAs, *DICER1* interacts with 233 miRNAs and *PTGFRN* interacts with 225 miRNAs, as listed in Table S7.

### 3.7. Construction of the Target Gene-TF Network

**Figure 14:**
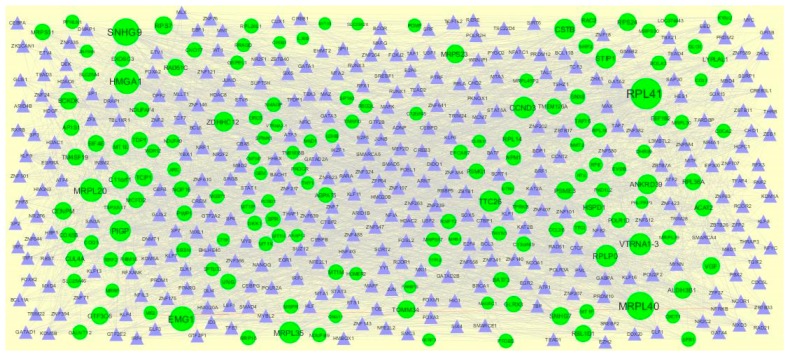
Transcription factor (TF)-gene network of predicted target up regulated genes. (Blue triangle—TFs and green circles—target up regulated genes).

**Figure 15:**
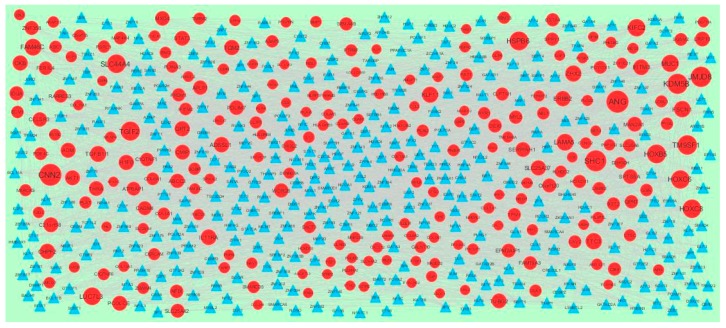
TF-gene network of predicted target down regulated genes. (Blue triangle—TFs and red circles—target down regulated genes).

Transcription factors (TFs) were responsible for the pathogenesis of various cancers [66]. The TFs for target up and down regulated genes are presented in Figure 14 and Figure 15, respectively. Top five up regulated targeted genes such as *RPL34* interacts with 47 TFs, *LOC374443* interacts with 42 TFs, *CENPM* interacts with 41 TFs, *TPRKB* interacts with 37 TFs, and *MRPL20* interacts with 35 TFs, which are listed in Table S8. Meanwhile the top five down regulated targeted genes such as *MYL5* interacts with 64 TFs, *NKX3-2* interacts with 59 TFs, *BGN* interacts with 55 TFs, *TPM1* interacts with 50 TFs and *FAT1* interacts with 47 TFs, as listed in Table S8.

### 3.8. Survival Curve Analysis of Hub Genes

**Figure 16:**
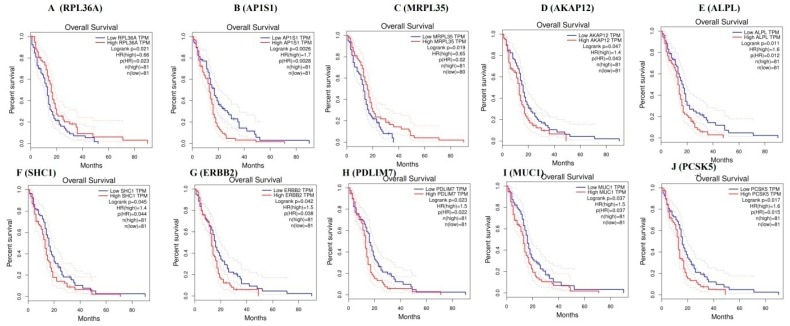
Kaplan-Meier survival curves using The Cancer Genome Atlas TCGA database validate the prognostic value of genes expressed in glioblastoma (blue—low risk; red—high risk).

TCGA data of cervical cancer patients are used via the GEPIA data portal. The Kaplan-Meier curve for overall survival of TCGA patients with GBM is obtained according to the low and high expression of each gene. The results showed that patients in the high mRNA expression group for *RPL36A* had favorable overall survival than those in the low expression group (*p* = 0.021) (Figure 16A), the high mRNA expression group for *AP1S1* had worse overall survival than those in the low expression group (*p* = 0.0026) (Figure 16B), the high mRNA expression group for *MRPL35* had favorable overall survival than those in the low expression group (*p* = 0.019) (Figure 16C), the high mRNA expression group for *AKAP12* had worse overall survival than those in the low expression group (*p* = 0.047) (Figure 16D), the high mRNA expression group for *ALPL* had worse overall survival than those in the low expression group (*p* = 0.011) (Figure 16E), the high mRNA expression group for *SHC1* had worse overall survival than those in the low expression group (*p* = 0.011) (Figure 16F), the high mRNA expression group for *ERBB2* had worse overall survival than those in the low expression group (*p* = 0.042) (Figure 16G), the high mRNA expression group for *PDLIM7* had favorable overall survival than those in the low expression group (*p* = 0.023) (Figure 16H), the high mRNA expression group for *MUC1* had worse overall survival than those in the low expression group (*p* = 0.037) (Figure 16I), and the high mRNA expression group for *PCSK5* had favorable overall survival than those in the low expression group (*p* = 0.017) (Figure 16J).

### 3.9. The mRNA Expression Levels of Hub Genes in GBM

**Figure 17:**
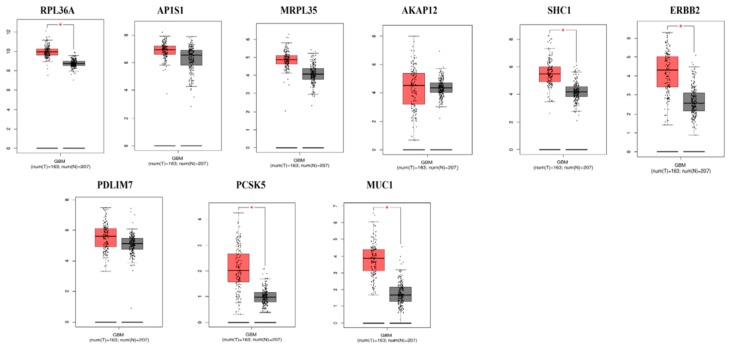
High expression levels of selected genes in glioblastoma multiform (GBM) using TCGA database.

**Figure 18:**
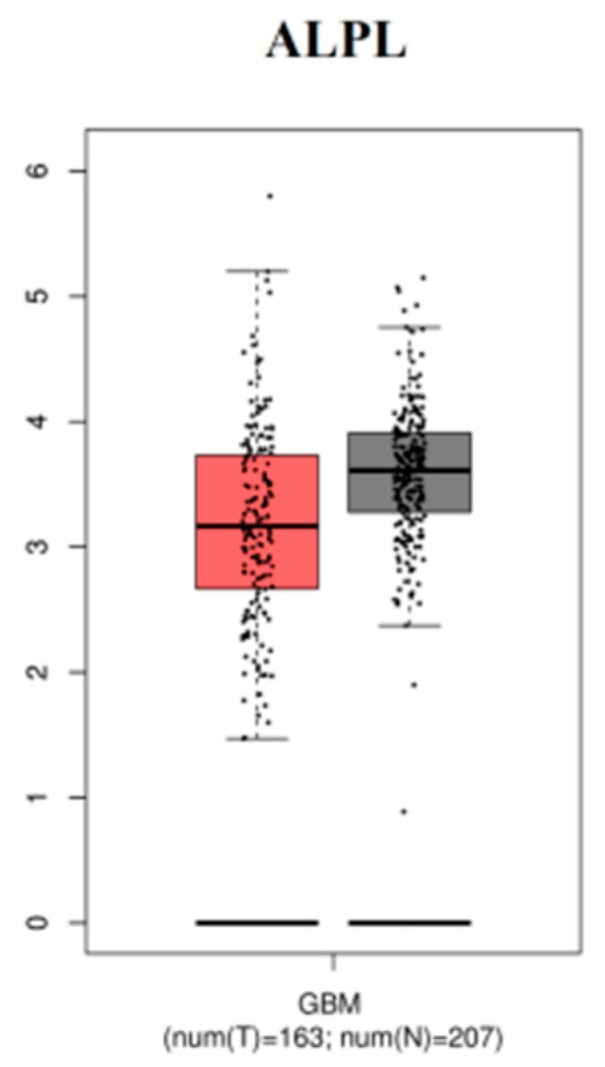
Low expression levels of selected gene in GBM using TCGA database.

The expression level of hub genes are assessed in 163 tumor patients and 207 normal patients. The data showed that the hub gene expression of *RPL36A*, *AP1S1*, *MRPL35*, *AKAP12*, *SHC1*, *ERBB2*, *PDLIM7*, *PCSK5*, and *MUC1* were increased (Figure 17), while that of expression of *ALPL* was reduced (Figure 18) in GBM compared with those in the normal patients.

### 3.10. Mutation Analysis

**Figure 19:**
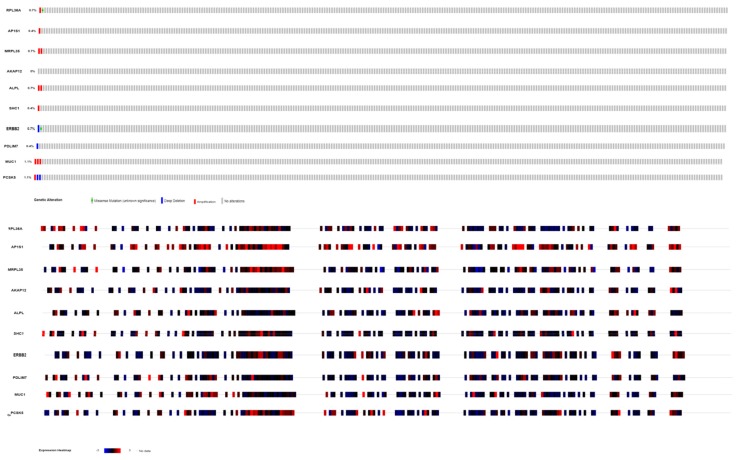
Mutation analysis (genetic mutation towards these ten hub genes).

Ten hub genes’ alteration status in TCGA GBM patients were analyzed using the CbioPortal database. Hub genes such as *RPL36A* (0.7% amplification and missense mutation), *AP1S1* (0.4% amplification), *MRPL35* (0.7% amplification), *AKAP12* (0% alteration), *ALPL* (0.7% amplification), *SHC1* (0.4% amplification), *ERBB2* (0.7% deep deletion and missense mutation), *PDLIM7* (0.4% deep deletion), *MUC1* (1.1% amplification) and *PCSK5* (1.1% amplification and deep deletion) were altered in patients and the frequency of alteration of each hub gene are shown in Figure 19.

## 4. Discussion

The cause of GBM has not been fully enlightened. It is accepted to contain environmental and genetic factors. Genetic factors are crucial to resolve the process of GBM occurrence and advancement [67]. However, no single causal gene has been determined. Instead, multiple genes may influence a person’s chance of advancing GBM, when initiated by environmental factors [68]. Microarray analysis may be used to compute the expression levels of large numbers of genes simultaneously, combined with bioinformatics analysis, this may allow for the recognition of the linked pathways and important genes with complex diseases. In the current study, analysis of E-MTAB-5871 gene expression profiles identified a total of 590 DEGs (243 up-and 347 down-regulated DEGs) between the scrambled shRNA expression and Lin7A knock down samples.

VGF was associated with development of pulmonary neuroendocrine cancer [69], but this gene may be responsible for development of GBM. Somatic mutations in tumor suppressor CNOT7 was responsible for the development of colorectal carcinoma [70], but loss of this gene may be linked with GBM. Genes such as *SYT13* [71] and *BCYRN1* [72] were responsible for invasion and migration of many cancer cells such as gastric cancer and lung cancer, but these genes may be associated with invasion and migration of GBM cells. Overexpression of genes such as *IL11RA* [73], BST2 [74] and GAS6 [75] were important for pathogenesis of many cancer types such as gastric cancer, breast cancer, and ovarian cancer, but high expression of these genes may be responsible for advancement of GBM. *FERMT2* was responsible for proliferation of esophageal squamous cancer cells [76], but this gene may be linked with proliferation of GBM. Mutations in PPFIBP2 [77] were involved in pathogenesis of prostate cancer, but alteration in this this gene may be important for the advancement of GBM. Genes such as *HRK* [78] and *ENG* [79] were responsible for the pathogenesis of GBM.

In pathway enrichment analysis for up regulated genes, genes such as *RPLP0* [80] and *MRPS30* [81] were associated with pathogenesis of many cancer types such as gastric cancer and breast cancer, but these genes may be responsible for advancement of GBM. Inactivation of tumor suppressor genes such as RPS7 [82], *MRPL39* [83], *MRPS23* [84], *ME2* [85], and *NDUFB9* [86] were diagnosed with the development of many cancer types such as colorectal cancer, gastric cancer, breast cancer, and pancreatic cancer, but loss of these genes may be responsible for the development of GBM. *RPS24* [87] was linked with the proliferation of colon cancer cells, but this gene may be linked with the proliferation of GBM cells. Polymorphism in tumor suppressor *RPL14* was associated with the pathogenesis of lung and oral cancers [88], but this polymorphic gene may be responsible for the growth of GBM. Genes such as *RPL34* [89], *GLO1* [90], *LDHB* [91], and *COX5B* [92] were responsible for pathogenesis of GBM. *RPL41*, *RPL36A*, *MRPL30*, *MRPL35*, *MRPS17*, *MRPL20*, *RPL26L1*, *MRPS31*, MRRF, *MRPL58*, *MRPL40*, *NDUFA9*, and *ATP6V0D2* were identified as novel biomarkers for the pathogenesis of GBM. Meanwhile, pathway enrichment analysis for down regulated genes, genes such as *MYL5* [93], *CAV2* [94], *BMP1* [95], *COL5A1* [96], *SERPINH1* [97], *COL7A1* [98], *COL8A1* [99], *COL13A1* [100], *BGN* [101], *SEMA4B* [102], *ECM1* [103], *TGFB3* [104], *ANGPTL2* [105], *MUC1* [106], and *CTSO* [107] were responsible for the invasion of many cancer cells such as cervical cancer, triple-negative breast cancer, lung cancer, esophageal squamous cell carcinoma, urothelial carcinoma, gastric cancer and breast cancer, but these genes may be liable for the invasion of GBM cells. MYLK was important for pathogenesis of breast cancer through the inhibition of apoptosis [108], but this gene may be associated with the inhibition of apoptosis in GBM. Over expression of *COL1A2* was important for the pathogenesis of gastric cancer [109], but high expression of this gene may be involved in development of GBM. Loss of *SHC1* was answerable for the development of pancreatic cancer [110], but inactivation of this gene may be involved in the pathogenesis of GBM. Genes such as *LAMA4* [111], *LAMA5* [112], and *C1QTNF6* [113] were involved in angiogenesis in many cancer types such as renal cell carcinoma, colorectal cancer, and hepatocellular carcinoma, but these genes may be linked with angiogenesis in GBM. Genes such as *COL5A2* [114], *SVEP1* [115], *NID2* [116], and *MMP23B* [117] were responsible for the growth of many cancer types such as bladder cancer and mammary adenocarcinoma, but these genes may be involved in the pathogenesis of GBM. Methylation inactivation of tumor suppressor genes such as *P3H2* [118] and *PAMR1* [119] were involved in the pathogenesis of breast cancer, but loss of these genes may be important for pathogenesis of GBM. CTSC was associated with proliferation of colorectal cancer cell [120], but this gene may be responsible for the proliferation of GBM cells. Genes such as *ERBB2* [121], PDGFRA [122], *PDGFRB* [123], *ITGA5* [124], *AKT1* [125], *COL1A1* [126], *COL6A1* [127], *THBS2* [128], *PLOD2* [129], *P4HA1* [130], *IL10* [131], *SPARC* [132], *IL18* [133], *SRPX2* [134], *CCL2* [135], *PLAT* [136], *PLAU* [137], *CXCL12* [138], *SFRP2* [139], *ANGPT1* [140], *CXCL14* [141], *TGM2* [142], *FSTL1* [143], *IGFBP5* [144], *IGFBP7* [145], *LOX* [146], and *LOXL1* [147] were diagnosed with the development of GBM. ACTN1, *COL4A5*, *COL6A2*, *LAMB2*, *P3H1*, *COL15A1*, *COL16A1*, *PCOLCE2*, *PCOLCE*, *PCSK5*, IK3IP1, *MFAP4*, *FAM20C*, *C1QTNF1*, *PLXNA3*, and *NTNG1* were identified as novel biomarkers for the pathogenesis of GBM.

In GO enrichment analysis for up regulated genes, genes such as EIF4E [148] and *RPS24* [87] were associated with cancer cell proliferation in many cancer types such as prostate cancer and colorectal cancer cells, but these genes may be responsible for the proliferation of GBM cells. Genes such as *NPM1* [149] and *CPEB1* [150] were diagnosed with the development of GBM. *RSL1D1*, *ETF1*, *MRPL30*, *SLC25A26*, *SNU13*, *C8orf88*, *SLC25A4*, *NHP2*, and *EEF1B2* were identified as novel biomarkers for the pathogenesis of GBM. Meanwhile, for GO enrichment analysis for down regulated genes, genes such as *GSN* [151], *FSCN1* [152], and *PHLDB2* [153] were important for the invasion of many cancer types such as pancreatic cancer, bladder cancer, and colon cancer cells, but these genes may be involved in the invasion of GBM. Somatic mutation in *LMCD1* is responsible for metastasis of hepatocellular carcinoma [154], but the loss of this gene may be diagnosed with metastasis of GBM. Genes such as *UPAR* [155] and *TGFB1I1* [156] were diagnosed with the development of GBM. *ABL1*, *RECK*, *DPP4*, *ERO1A*, *APLP1*, *LCP1*, *MAMDC2*, and *CASK* were identified as novel biomarkers for the pathogenesis of GBM.

In the PPI network (up regulated), genes such as *CUL4A* [157] and *GLRX3* [158] were responsible for the invasion of many cancer types such as prostate cancer and oral squamous cell carcinoma, but these genes may be identified with the invasion of GBM. Genes such as *PSME3* [159] and *HSPD1* [160] were involved in the pathogenesis of many cancer types such as colorectal cancer and colon cancer, but these genes may be important for the growth of GBM. Alteration in *RAC2* was responsible for pathogenesis of GBM [161]. *CENPM*. *HEBP1*, *YIPF1*, *EFCAB7*, *SPTLC3*, and *ZNF586* were novel biomarkers for the pathogenesis of GBM. In the PPI network (down regulated), genes such as *STAT3* [162], *HNRNPA2B1* [163], and *SSTR2* [164] were responsible for the pathogenesis of GBM. Methylation inactivation of tumor suppressor gene FILIP1L was important for pathogenesis of prostate cancer [165], but loss of this gene may be associated with the development of GBM. Low expression of tumor suppressor *TCEA3* is liable for pathogenesis of ovarian cancer [166], but decreased expression of this gene may be identified with the growth of GBM. *VLDLR*, *FIBCD1*, and *UBL3* were identified as novel biomarkers for pathogenesis of GBM.

In module analysis for the PPI network (up regulated), *TCP1* was responsible for the growth of GBM [167]. *PSMG1* and *POMP* were novel biomarkers for the pathogenesis of GBM. Meanwhile module analysis for the PPI network (down regulated), methylation inactivation of tumor suppressor gene *CELSR3* was identified with the development of oral squamous cell carcinoma [168], but loss of this gene may be responsible for the pathogenesis of GBM. Genes such as *HOXC8* [169] and *NCOR2* [170] were associated with the pathogenesis of GBM. *AIRE* and *SMARCD3* were novel biomarkers for the pathogenesis of GBM.

In target genes-miRNA network (up regulated), DTNA was a novel biomarker for the pathogenesis of GBM. Meanwhile, in target genes-miRNA network (down regulated), WDR26 was responsible for the invasion of breast cancer cells [171], but this gene may be identified with invasion of GBM. *PRDM1* was responsible for pathogenesis of GBM [172]. Modification in tumor suppressor *DICER1* was liable for the pathogenesis of ovarian cancers [173], but mutation in this gene may be diagnosed with the growth of GBM. *FAM126B* and *PTGFRN* were identified as novel biomarkers for the pathogenesis of GBM.

In target genes-TF network (up regulated), *LOC374443* and *TPRKB* were novel biomarkers for pathogenesis of GBM. Meanwhile, in target genes-TF network (down regulated), genes such as TPM1 [174] and *FAT1* [175] were diagnosed with growth of GBM. NKX3-2 was a novel biomarker for pathogenesis of GBM.

Survival analysis revealed that genes such as *RPL36A*, *MRPL35*, *PDLIM7*, and *PCSK5* were predicting longer survival of GBM, while *AP1S1*, *AKAP12*, *ALPL*, *SHC1*, *ERBB2*, and *MUC1* were predicting shorter survival of GBM. High expression of genes such as *RPL36A*, *AP1S1*, *MRPL35*, *AKAP12*, *SHC1*, *ERBB2*, *PDLIM7*, *PCSK5*, and *MUC1* were linked with GBM, while low expression of genes such as *ALPL* was linked with the pathogenesis of GBM. *AKAP12* were associated with the development of GBM [176]. From the TCGA database, genes such as *AP1S1*, *RPL36A*, and *MRPL35* were expressed in GBM.

These findings could significantly enhance our understanding of the cause and underlying molecular mechanisms in GBM, these candidate genes and pathways could serve as new prognostic biomarkers and therapeutic targets for GBM. In the future, these findings will assist in developing and designing new diagnostic and therapeutic agents for the management of GBM.

## 5. Conclusions

In conclusion, several hub genes and pathways diagnosed in the current study may be associated in the molecular mechanism of GBM pathogenesis. These results obtained from gene expression microarray data may improve our understanding of molecular mechanisms underlying GBM. However, further validation of these hub genes and pathways diagnosed in the current study will be required to investigate the pathogenic molecular mechanisms of GBM.

## Supplementary Materials

The following are available online at https://www.mdpi.com/2218-273X/9/5/201/s1, Table S1. The statistical metrics for key differentially expressed genes (DEGs). Table S2. The enriched pathway terms of the up-regulated differentially expressed genes. Table S3. The enriched pathway terms of the down-regulated differentially expressed genes. Table S4. The enriched GO terms of the up-regulated differentially expressed genes. Table S5. The enriched GO terms of the down-regulated differentially expressed genes. Table S6. Topology table for up and down regulated genes. Table S7. Target gene- miRNA interaction table. Table S8. Target gene-TF interaction table.
