# Supplementary material for: Identification of Crucial Candidate Genes and Pathways in Glioblastoma Multiform by Bioinformatics Analysis
Source: Biomolecules. 2019 May 24;9(5):201. doi: 10.3390/biom9050201 (PMC6571969; doi:10.3390/biom9050201)
Supplement: Supplementary file 1 [file biomolecules-09-00201-s001.pdf]

Table 1 The statistical metrics for key differentially expressed genes (DEGs)

Illumina Id	Gene Symbol	logFC	p Value	FDR	t value	Regulation	Gene name
ILMN_1757497	VGF	4.848711	1.27E-13	6.11E-09	48.36804	UP	VGF nerve growth factor inducible
ILMN_2193706	HRK	3.058491	2.89E-10	3.47E-06	22.91096	UP	harakiri, BCL2 interacting protein
ILMN_2182198	MRPL58	1.8013	5.14E-10	3.64E-06	21.65441	UP	mitochondrial ribosomal protein L58
ILMN_1699772	RRAGD	2.331637	6.56E-10	3.64E-06	21.14388	UP	Ras related GTP binding D
ILMN_1695969	TMPRSS15	2.158671	6.81E-10	3.64E-06	21.06686	UP	transmembrane protease, serine 15
ILMN_1754002	IL36B	4.161387	3.21E-09	1.10E-05	18.09187	UP	interleukin 36, beta
ILMN_1656134	CNOT7	1.387716	4.97E-09	1.49E-05	17.32692	UP	CCR4-NOT transcription complex subunit 7
ILMN_1658499	SYT13	1.653015	6.80E-09	1.59E-05	16.79842	UP	synaptotagmin 13
ILMN_1684836	AKAP12	1.291595	8.20E-09	1.68E-05	16.48925	UP	A-kinase anchoring protein 12
ILMN_1799519	IL36B	4.299495	9.58E-09	1.68E-05	16.23691	UP	interleukin 36, beta
ILMN_1678757	BCYRN1	3.056229	9.58E-09	1.68E-05	16.23661	UP	brain cytoplasmic RNA 1
ILMN_1726743	MRPS30	1.302467	1.08E-08	1.73E-05	16.04765	UP	mitochondrial ribosomal protein S30
ILMN_1777976	SLC25A26	1.609861	1.37E-08	2.06E-05	15.66752	UP	solute carrier family 25 member 26
ILMN_3308961	MT-ND6	3.019664	1.61E-08	2.15E-05	15.41788	UP	mitochondrially encoded NADH:ubiquinoneoxidoreductase core subunit 6
ILMN_2380999	RECQL	1.437639	2.36E-08	2.72E-05	14.83968	UP	RecQ like helicase
ILMN_3233229	SNHG7	1.202524	2.50E-08	2.72E-05	14.7569	UP	small nucleolar RNA host gene 7
ILMN_1693287	POMP	1.568757	2.55E-08	2.72E-05	14.7311	UP	proteasome maturation protein
ILMN_1710001	RPL41	1.285956	3.09E-08	2.96E-05	14.44915	UP	ribosomal protein L41
ILMN_1677092	GEM	1.73552	3.79E-08	3.31E-05	14.15535	UP	GTP binding protein overexpressed in skeletal muscle
ILMN_1712806	AP1S1	1.469754	3.98E-08	3.40E-05	14.08686	UP	adaptor related protein complex 1 sigma 1 subunit
ILMN_1664167	RPF2	1.057697	4.03E-08	3.40E-05	14.06673	UP	ribosome production factor 2 homolog
ILMN_1766154	MRPL30	1.189265	4.88E-08	3.79E-05	13.79948	UP	mitochondrial ribosomal protein L30
ILMN_1784320	ELMO1	1.385612	5.45E-08	4.03E-05	13.64781	UP	engulfment and cell motility 1
ILMN_1733998	DHRS9	1.302631	5.99E-08	4.29E-05	13.51785	UP	dehydrogenase/reductase 9
ILMN_1726391	MRPL39	1.233196	6.24E-08	4.29E-05	13.46276	UP	mitochondrial ribosomal protein L39
ILMN_1799889	ATP6V0D2	1.155423	6.63E-08	4.29E-05	13.3809	UP	ATPase H+ transporting V0 subunit d2
ILMN_1802355	RBM18	1.079522	6.90E-08	4.31E-05	13.32626	UP	RNA binding motif protein 18
ILMN_1692545	NPM1	1.062554	8.15E-08	4.59E-05	13.10498	UP	Nucleophosmin 1
ILMN_2124802	MT1H	1.455292	8.40E-08	4.59E-05	13.06373	UP	metallothionein 1H
ILMN_1680850	RECQL	1.309379	8.46E-08	4.59E-05	13.05513	UP	RecQ like helicase
ILMN_2068104	TFPI2	2.166826	8.49E-08	4.59E-05	13.05078	UP	tissue factor pathway inhibitor 2
ILMN_1779875	THY1	2.142515	8.79E-08	4.59E-05	13.00372	UP	Thy-1 cell surface antigen
ILMN_1760741	NDUFA9	1.628833	8.88E-08	4.59E-05	12.99102	UP	NADH:ubiquinoneoxidoreductase subunit A9
ILMN_1770692	WDR12	1.101995	9.88E-08	4.95E-05	12.8511	UP	WD repeat domain 12
ILMN_2369603	CPEB1	1.347261	1.08E-07	5.30E-05	12.73577	UP	cytoplasmic polyadenylation element binding protein 1
ILMN_1750981	SLC25A26	1.080503	1.10E-07	5.35E-05	12.71031	UP	solute carrier family 25 member 26
ILMN_1715886	CNOT7	1.167941	1.22E-07	5.69E-05	12.58376	UP	CCR4-NOT transcription complex subunit 7
ILMN_1659601	CCL26	1.031457	1.33E-07	5.93E-05	12.46884	UP	C-C motif chemokine ligand 26
ILMN_1702177	GLO1	1.195556	1.36E-07	5.93E-05	12.44489	UP	glyoxalase I
ILMN_1743303	TTC1	1.132109	1.37E-07	5.94E-05	12.43127	UP	tetratricopeptide repeat domain 1
ILMN_3199609	TCP1	1.210626	1.50E-07	6.32E-05	12.31937	UP	t-complex 1
ILMN_2357361	THYN1	1.055324	1.69E-07	6.55E-05	12.16926	UP	thymocyte nuclear protein 1
ILMN_3228822	NEMP1	1.230904	1.71E-07	6.59E-05	12.15278	UP	nuclear envelope integral membrane protein 1
ILMN_2404850	RPL14	1.396325	2.11E-07	7.74E-05	11.89807	UP	ribosomal protein L14
ILMN_1723729	RSL1D1	1.132765	2.15E-07	7.74E-05	11.87064	UP	ribosomal L1 domain containing 1
ILMN_1693345	CPEB1	1.242623	2.20E-07	7.77E-05	11.84682	UP	cytoplasmic polyadenylation element binding protein 1
ILMN_1678655	PNLIPRP3	2.600287	2.27E-07	7.95E-05	11.8085	UP	pancreatic lipase related protein 3
ILMN_3244090	GAGE12H	1.490422	2.30E-07	7.95E-05	11.79327	UP	G antigen 12H
ILMN_1749930	NDC1	1.111263	2.38E-07	8.11E-05	11.75256	UP	NDC1 transmembranenucleoporin
ILMN_1791792	TIGAR	1.316672	2.44E-07	8.21E-05	11.71987	UP	TP53 induced glycolysis regulatory phosphatase
ILMN_2181593	MAGEA1	1.692059	2.47E-07	8.26E-05	11.70429	UP	MAGE family member A1
ILMN_3273885	C8orf88	1.020644	2.77E-07	8.84E-05	11.56737	UP	chromosome 8 open reading frame 88
ILMN_3192446	NPM1	1.139428	2.87E-07	8.92E-05	11.52518	UP	nucleophosmin
ILMN_1695959	EVA1C	1.095958	3.04E-07	9.31E-05	11.45996	UP	eva-1 homolog C
ILMN_3231944	NMT2	1.978529	3.11E-07	9.42E-05	11.43103	UP	N-myristoyltransferase 2
ILMN_1705849	SPR	1.368984	3.34E-07	9.74E-05	11.34867	UP	sepiapterinreductase (7,8-dihydrobiopterin:NADP+ oxidoreductase)
ILMN_1685856	FAM92A	1.127165	3.41E-07	9.81E-05	11.32616	UP	family with sequence similarity 92 member A
ILMN_1690307	TPRKB	1.536421	3.41E-07	9.81E-05	11.32573	UP	TP53RK binding protein
ILMN_1670817	GRPEL1	1.206391	3.46E-07	9.82E-05	11.30684	UP	GrpE like 1, mitochondrial
ILMN_1767422	POLR1D	1.422704	3.58E-07	0.0001	11.26729	UP	RNA polymerase I subunit D
ILMN_2367883	GEM	1.93171	3.68E-07	0.000102	11.23462	UP	GTP binding protein overexpressed in skeletal muscle
ILMN_1803799	EIF4E	1.01955	3.77E-07	0.000103	11.20947	UP	eukaryotic translation initiation factor 4E
ILMN_1710979	ANKRD39	1.01801	3.88E-07	0.000105	11.17465	UP	ankyrin repeat domain 39
ILMN_3299905	RNFT2	1.610512	4.15E-07	0.000108	11.09644	UP	ring finger protein, transmembrane 2
ILMN_1789510	STIP1	1.512192	4.23E-07	0.000109	11.07692	UP	stress induced phosphoprotein 1
ILMN_1698934	CMTM7	1.025624	4.37E-07	0.00011	11.0397	UP	CKLF like MARVEL transmembrane domain containing 7
ILMN_1671486	HOMER2	1.552027	4.38E-07	0.00011	11.03608	UP	homer scaffolding protein 2
ILMN_1728132	LDHB	1.029818	4.75E-07	0.000117	10.94328	UP	lactate dehydrogenase B

ILMN_1736757	GNPTAB	1.012532	4.82E-07	0.000118	10.92836	UP	N-acetylglucosamine-1-phosphate transferase alpha and beta subunits
ILMN_3305111	AL513283.1	2.341719	4.96E-07	0.00012	10.89566	UP	NA
ILMN_1657435	MT1M	1.177419	4.96E-07	0.00012	10.89537	UP	metallothionein 1M
ILMN_2070072	RPS7	1.932593	5.00E-07	0.00012	10.88626	UP	ribosomal protein S7
ILMN_1808325	TM4SF19	1.369966	5.19E-07	0.000122	10.8437	UP	transmembrane 4 L six family member 19
ILMN_3265742	NA	2.011555	5.28E-07	0.000124	10.82489	UP	NA
ILMN_1703301	MRPL45P2	1.091498	5.35E-07	0.000125	10.80962	UP	mitochondrial ribosomal protein L45 pseudogene 2
ILMN_2056479	GLRX3	1.08002	5.43E-07	0.000126	10.79259	UP	glutaredoxin 3
ILMN_2316173	AP1S1	1.40468	5.96E-07	0.000132	10.6893	UP	adaptor related protein complex 1 sigma 1 subunit
ILMN_1737514	KYNU	2.20214	6.20E-07	0.000135	10.64563	UP	kynureninase
ILMN_1678707	TAF15	1.504327	6.38E-07	0.000137	10.61446	UP	TATA-box binding protein associated factor 15
ILMN_1687359	MRPS23	1.224253	6.44E-07	0.000138	10.60352	UP	mitochondrial ribosomal protein S23
ILMN_1750722	RPS7	1.828962	6.53E-07	0.000138	10.58955	UP	ribosomal protein S7
ILMN_1667711	PLA2G16	1.15827	6.57E-07	0.000138	10.58152	UP	phospholipase A2 group XVI
ILMN_1712082	GCNT3	2.485409	6.61E-07	0.000138	10.57537	UP	glucosaminyl (N-acetyl) transferase 3, mucin type
ILMN_2311537	HMGA1	1.761646	6.64E-07	0.000138	10.57017	UP	high mobility group AT-hook 1
ILMN_3226814	NA	1.307365	7.47E-07	0.00015	10.44152	UP	NA
ILMN_3243856	NA	1.097654	7.57E-07	0.000152	10.42734	UP	NA
ILMN_2346479	HOMER2	1.070292	8.15E-07	0.000153	10.34718	UP	homer scaffolding protein 2
ILMN_3306440	NEMP1	1.045549	8.63E-07	0.000155	10.28533	UP	nuclear envelope integral membrane protein 1
ILMN_2402131	TAF15	1.200247	8.64E-07	0.000155	10.28401	UP	TATA-box binding protein associated factor 15
ILMN_1713829	PTGES	2.934372	8.65E-07	0.000155	10.28321	UP	prostaglandin E synthase
ILMN_1746517	KYNU	1.992538	8.73E-07	0.000156	10.27303	UP	kynureninase
ILMN_1782705	GAGE13	1.891453	8.82E-07	0.000156	10.26201	UP	G antigen 13
ILMN_1674046	C10orf128	1.750908	8.93E-07	0.000157	10.2484	UP	chromosome 10 open reading frame 128
ILMN_1728435	C12orf45	1.107955	9.18E-07	0.000159	10.21941	UP	chromosome 12 open reading frame 45
ILMN_1774949	PIGP	1.047814	9.37E-07	0.000161	10.19701	UP	phosphatidylinositol glycan anchor biosynthesis class P
ILMN_1660654	CDCA2	1.055928	9.62E-07	0.000164	10.16923	UP	cell division cycle associated 2
ILMN_1743049	PWP1	1.01807	9.79E-07	0.000166	10.15074	UP	PWP1 homolog, endonuclein
ILMN_1751345	AP1S1	1.647354	1.02E-06	0.000169	10.10234	UP	adaptor related protein complex 1 sigma 1 subunit
ILMN_1786105	PCBD1	1.013255	1.13E-06	0.000182	9.99934	UP	pterin-4 alpha-carbinolaminedehydratase 1
ILMN_2343010	BOLA3	1.497888	1.14E-06	0.000183	9.992888	UP	bolA family member 3
ILMN_1735157	GALNT12	1.415897	1.17E-06	0.000185	9.967359	UP	polypeptide N-acetylgalactosaminyltransferase 12
ILMN_1917095	LINC02454	1.307938	1.19E-06	0.000186	9.947329	UP	long intergenic non-protein coding RNA 2454
ILMN_2415949	MRRF	1.084664	1.21E-06	0.000188	9.924701	UP	mitochondrial ribosome recycling factor
ILMN_1801378	COQ3	1.685823	1.23E-06	0.000188	9.907374	UP	coenzyme Q3, methyltransferase
ILMN_1797074	EMG1	1.720984	1.28E-06	0.000194	9.87203	UP	EMG1, N1-specific pseudouridinemethyltransferase
ILMN_1698673	EFCAB7	1.276151	1.31E-06	0.000196	9.847564	UP	EF-hand calcium binding domain 7
ILMN_1706873	RPL34	1.315152	1.36E-06	0.000202	9.803535	UP	ribosomal protein L34
ILMN_2241627	MAGEC1	1.086707	1.38E-06	0.000203	9.791266	UP	MAGE family member C1
ILMN_1715638	GAGE2A	1.953782	1.48E-06	0.000212	9.720376	UP	G antigen 2A
ILMN_1676555	TTC26	1.118373	1.49E-06	0.000213	9.712673	UP	tetratricopeptide repeat domain 26
ILMN_2346573	PSME3	1.654012	1.50E-06	0.000214	9.708095	UP	proteasome activator subunit 3
ILMN_1652464	TUBA3E	1.227156	1.52E-06	0.000214	9.694091	UP	tubulin alpha 3e
ILMN_1675186	ME2	1.145395	1.64E-06	0.000225	9.614552	UP	malic enzyme 2
ILMN_1708672	ACAT2	2.254208	1.72E-06	0.000233	9.564453	UP	acetyl-CoA acetyltransferase 2
ILMN_1654552	MRPS31	1.007677	1.81E-06	0.000237	9.512447	UP	mitochondrial ribosomal protein S31
ILMN_2285713	TDP1	1.736008	1.85E-06	0.000238	9.495635	UP	tyrosyl-DNA phosphodiesterase 1
ILMN_2084353	M6PR	1.190688	1.85E-06	0.000238	9.491345	UP	mannose-6-phosphate receptor, cation dependent
ILMN_1671905	SFR1	1.336209	1.87E-06	0.000238	9.484456	UP	SWI5 dependent homologous recombination repair protein 1
ILMN_1786658	BOLA3	1.531441	1.90E-06	0.000241	9.468811	UP	bolA family member 3
ILMN_3242920	GAGE12C	1.211134	1.95E-06	0.000243	9.44203	UP	G antigen 12C
ILMN_2157240	MNS1	1.398621	1.95E-06	0.000243	9.441392	UP	meiosis specific nuclear structural 1
ILMN_3247723	NOP16	1.320803	2.02E-06	0.000247	9.404662	UP	NOP16 nucleolar protein
ILMN_1660661	TCP1	1.396472	2.03E-06	0.000248	9.399537	UP	t-complex 1
ILMN_1747622	CD33	1.149505	2.07E-06	0.00025	9.380458	UP	CD33 molecule
ILMN_1695792	CUL4A	1.109981	2.15E-06	0.000254	9.34153	UP	cullin 4A
ILMN_1663131	LYST	1.745141	2.16E-06	0.000254	9.338334	UP	lysosomal trafficking regulator
ILMN_2399893	RPS24	1.748777	2.23E-06	0.000256	9.308712	UP	ribosomal protein S24
ILMN_1697614	SNU13	1.084495	2.26E-06	0.000258	9.295568	UP	small nuclear ribonucleoprotein 13
ILMN_1766713	HSPD1	1.071611	2.26E-06	0.000258	9.291972	UP	heat shock protein family D (Hsp60) member 1
ILMN_1685678	EEF1B2	1.222237	2.33E-06	0.000262	9.262429	UP	eukaryotic translation elongation factor 1 beta 2
ILMN_1659524	NDUFAF4	1.090084	2.34E-06	0.000262	9.258825	UP	NADH:ubiquinoneoxidoreductase complex assembly factor 4
ILMN_1701603	ALPL	1.426589	2.36E-06	0.000263	9.252812	UP	alkaline phosphatase, liver/bone/kidney
ILMN_1804901	IL36RN	2.728795	2.50E-06	0.000272	9.195772	UP	interleukin 36 receptor antagonist
ILMN_1681301	AIM2	1.372047	2.51E-06	0.000272	9.191419	UP	absent in melanoma 2
ILMN_1803564	YIPF1	1.150704	2.51E-06	0.000272	9.191146	UP	Yip1 domain family member 1
ILMN_1685834	AMPH	1.163425	2.51E-06	0.000272	9.19098	UP	amphiphysin
ILMN_1664660	GAGE12D	1.826058	2.76E-06	0.000291	9.09728	UP	G antigen 12D
ILMN_1763452	EVI2B	1.012762	2.83E-06	0.000295	9.072004	UP	ecotropic viral integration site 2B
ILMN_1803033	MGST1	1.023526	2.84E-06	0.000295	9.069653	UP	microsomal glutathione S-transferase 1
ILMN_2189424	MRPL20	1.608898	2.87E-06	0.000296	9.05995	UP	mitochondrial ribosomal protein L20
ILMN_1693394	BCKDK	1.063651	2.90E-06	0.000296	9.050618	UP	branched chain ketoacid dehydrogenase kinase
ILMN_1738450	GAGE12H	1.823061	2.96E-06	0.000301	9.029987	UP	G antigen 12H

ILMN_1674097	GAGE2B	1.869184	3.00E-06	0.000304	9.017238	UP	G antigen 2B
ILMN_1721128	TOMM34	1.497091	3.27E-06	0.00032	8.93444	UP	translocase of outer mitochondrial membrane 34
ILMN_1761808	MCFD2	1.519915	3.28E-06	0.00032	8.932162	UP	multiple coagulation factor deficiency 2
ILMN_1708905	LOC374443	1.187282	3.29E-06	0.000321	8.928105	UP	C-type lectin domain family 2 member D pseudogene
ILMN_1703370	ZDHHC12	1.393931	3.30E-06	0.000321	8.924104	UP	zinc finger DHHC-type containing 12
ILMN_1795906	PCBD1	1.03727	3.36E-06	0.000324	8.907353	UP	pterin-4 alpha-carbinolaminedehydratase 1
ILMN_1698179	TAGLN3	1.304215	3.42E-06	0.000326	8.892099	UP	transgelin 3
ILMN_1813544	OXCT1	1.102376	3.48E-06	0.00033	8.87429	UP	3-oxoacid CoA-transferase 1
ILMN_1666727	ZNF586	1.041322	3.55E-06	0.000334	8.85518	UP	zinc finger protein 586
ILMN_1761927	IL36B	2.550482	3.60E-06	0.000336	8.842537	UP	interleukin 36, beta
ILMN_2128967	C11orf1	1.028822	3.64E-06	0.000339	8.831338	UP	chromosome 11 open reading frame 1
ILMN_2195385	GAGE1	1.907208	3.82E-06	0.000347	8.786313	UP	G antigen 1
ILMN_2233576	NA	1.834413	3.85E-06	0.000347	8.778871	UP	NA
ILMN_1705093	ORC5	1.341777	3.86E-06	0.000347	8.775223	UP	origin recognition complex subunit 5
ILMN_1678140	MROH7-TTC4	1.07432	4.10E-06	0.000362	8.720597	UP	MROH7-TTC4 readthroug
ILMN_1668721	CCND3	1.251494	4.10E-06	0.000362	8.719668	UP	cyclin D3
ILMN_1819384	VTRNA1-3	1.983731	4.30E-06	0.000375	8.674797	UP	vault RNA 1-3
ILMN_2332250	ACOT7	1.049651	4.30E-06	0.000375	8.674457	UP	acyl-CoA thioesterase 7
ILMN_3219455	HSPD1	1.158325	4.32E-06	0.000375	8.669912	UP	heat shock protein family D (Hsp60) member 1
ILMN_2322842	PPHLN1	1.154526	4.44E-06	0.000384	8.64436	UP	periphilin 1
ILMN_2161556	C12orf49	1.228683	4.46E-06	0.000385	8.640758	UP	chromosome 12 open reading frame 49
ILMN_2413644	TM4SF19	1.657306	4.87E-06	0.000405	8.558584	UP	transmembrane 4 L six family member 19
ILMN_2380243	SMAGP	1.205263	4.93E-06	0.000408	8.548385	UP	small cell adhesion glycoprotein
ILMN_1764690	NTS	1.02382	4.94E-06	0.000408	8.544959	UP	neurotensin
ILMN_3243333	GAGE12F	1.541751	4.95E-06	0.000408	8.544463	UP	G antigen 12F
ILMN_3310643	VTRNA2-1	1.095224	5.05E-06	0.000414	8.525196	UP	vault RNA 2-1
ILMN_1711120	ARC	1.026855	5.09E-06	0.000416	8.518528	UP	activity regulated cytoskeleton associated protein
ILMN_1782419	GNG11	1.229156	5.18E-06	0.000422	8.501468	UP	G protein subunit gamma 11
ILMN_1804851	MRPS17	1.28852	5.22E-06	0.000422	8.49548	UP	mitochondrial ribosomal protein S17
ILMN_1745075	RPLP0	1.063149	5.37E-06	0.000426	8.469559	UP	ribosomal protein lateral stalk subunit P0
ILMN_1792456	CFAP36	1.097784	5.39E-06	0.000426	8.465776	UP	cilia and flagella associated protein 36
ILMN_1738093	RNFT2	2.110618	5.69E-06	0.000441	8.416105	UP	ring finger protein, transmembrane 2
ILMN_2112811	RPL36A	1.436589	5.79E-06	0.000442	8.400418	UP	ribosomal protein L36a
ILMN_1672611	CDH11	1.717565	5.96E-06	0.000453	8.37399	UP	cadherin 11
ILMN_2341952	MRPL35	1.098105	6.00E-06	0.000455	8.367775	UP	mitochondrial ribosomal protein L35
ILMN_1728662	ALDH3B1	1.016702	6.17E-06	0.000462	8.341404	UP	aldehyde dehydrogenase 3 family member B1
ILMN_3238785	SNHG9	1.436476	6.25E-06	0.000465	8.33019	UP	small nucleolar RNA host gene 9
ILMN_1787266	SPINK1	2.900279	6.27E-06	0.000465	8.327838	UP	serine peptidase inhibitor, Kazal type 1
ILMN_2136089	MT1IP	1.708939	6.33E-06	0.000468	8.318097	UP	metallothionein 1I, pseudogene
ILMN_1718766	MT1F	1.957094	6.48E-06	0.000473	8.297502	UP	metallothionein 1F
ILMN_2217809	TMEM126A	1.409649	6.66E-06	0.000479	8.273376	UP	transmembrane protein 126A
ILMN_1773337	DKK1	1.331397	6.72E-06	0.000481	8.264075	UP	dickkopf WNT signaling pathway inhibitor 1
ILMN_1705753	CMSS1	1.101981	6.82E-06	0.000484	8.250737	UP	cms1 ribosomal small subunit homolog (yeast)
ILMN_2222991	ETF1	1.080008	6.86E-06	0.000486	8.245505	UP	eukaryotic translation termination factor 1
ILMN_1662640	MT1P3	1.4199	7.14E-06	0.000496	8.209481	UP	metallothionein 1 pseudogene 3
ILMN_1783832	GAGE6	1.536703	7.62E-06	0.000515	8.151553	UP	G antigen 6
ILMN_1661997	NA	1.210329	7.71E-06	0.000518	8.141577	UP	NA
ILMN_1804051	SNX8	1.00603	8.01E-06	0.000529	8.107263	UP	sorting nexin 8
ILMN_2148796	MND1	1.209682	8.92E-06	0.000567	8.012479	UP	meiotic nuclear divisions 1
ILMN_1800975	PSME3	1.636721	9.06E-06	0.000572	7.998141	UP	proteasome activator subunit 3
ILMN_2355168	MGST1	1.706965	9.10E-06	0.000573	7.99415	UP	microsomal glutathione S-transferase 1
ILMN_2374383	TSPAN17	1.209962	9.11E-06	0.000573	7.993702	UP	tetraspanin 17
ILMN_1683120	UNG	1.000572	9.19E-06	0.000574	7.98555	UP	uracil DNA glycosylase
ILMN_1662049	AGPAT5	1.049626	9.37E-06	0.000577	7.969148	UP	1-acylglycerol-3-phosphate O-acyltransferase 5
ILMN_1712759	SBSN	1.553921	9.56E-06	0.000585	7.951394	UP	suprabasin
ILMN_1758895	CTSK	1.190477	9.76E-06	0.000594	7.933106	UP	cathepsin K
ILMN_1763207	BATF3	1.081974	1.01E-05	0.000605	7.907033	UP	basic leucine zipper ATF-like transcription factor 3
ILMN_2246882	SP140	1.838916	1.02E-05	0.000609	7.893965	UP	SP140 nuclear body protein
ILMN_3247835	PRR32	1.029526	1.02E-05	0.000609	7.892505	UP	proline rich 32
ILMN_2274586	PKD1L2	1.037612	1.05E-05	0.000622	7.86973	UP	polycystin 1 like 2 (gene/pseudogene)
ILMN_1726460	RPL14	1.439308	1.06E-05	0.000625	7.859055	UP	ribosomal protein L14
ILMN_1701058	TATDN1	1.057867	1.07E-05	0.000626	7.856357	UP	TatDDNase domain containing 1
ILMN_2268068	MAPKAP1	1.329536	1.08E-05	0.00063	7.848348	UP	mitogen-activated protein kinase associated protein 1
ILMN_1761797	CSTB	1.397528	1.09E-05	0.000633	7.840339	UP	cystatin B
ILMN_1687403	MRPL40	1.056672	1.11E-05	0.000642	7.820164	UP	mitochondrial ribosomal protein L40
ILMN_1734194	EXOSC3	1.110517	1.12E-05	0.000646	7.810958	UP	exosome component 3
ILMN_3207122	GTF3C6	1.543199	1.13E-05	0.00065	7.802124	UP	general transcription factor IIIC subunit 6
ILMN_1685661	RRP15	1.096084	1.18E-05	0.000667	7.765888	UP	ribosomal RNA processing 15 homolog
ILMN_1781952	MGST1	1.884082	1.19E-05	0.000668	7.762078	UP	microsomal glutathione S-transferase 1
ILMN_1663512	COX5B	1.290675	1.19E-05	0.000668	7.761648	UP	cytochrome c oxidase subunit 5B
ILMN_3226807	NHP2	1.003154	1.21E-05	0.000673	7.748162	UP	NHP2 ribonucleoprotein
ILMN_1757278	HSPD1	1.169032	1.21E-05	0.000674	7.744797	UP	heat shock protein family D (Hsp60) member 1
ILMN_1717262	PROCR	1.254319	1.34E-05	0.000722	7.656769	UP	protein C receptor
ILMN_1709795	RAC2	1.048994	1.34E-05	0.000722	7.656547	UP	ras-related C3 botulinum toxin substrate 2 (rho family, small GTP binding protein Rac2)
ILMN_1730201	DTNA	1.505244	1.40E-05	0.000743	7.623916	UP	dystrobrevin alpha

ILMN_1739345	LBHD1	1.104877	1.41E-05	0.00075	7.614525	UP	LBH domain containing 1
ILMN_1704055	NOP16	1.306487	1.47E-05	0.000777	7.579211	UP	NOP16 nucleolar protein
ILMN_3227023	SNHG7	1.221204	1.48E-05	0.000777	7.576414	UP	small nucleolar RNA host gene 7
ILMN_1652549	DTNA	1.003144	1.50E-05	0.000782	7.563748	UP	dystrobrevin alpha
ILMN_1720311	SLC25A46	1.015267	1.53E-05	0.000792	7.548638	UP	solute carrier family 25 member 46
ILMN_2233454	SPTLC3	1.270102	1.54E-05	0.000796	7.539245	UP	serine palmitoyltransferase long chain base subunit 3
ILMN_2368721	CENPM	1.097911	1.56E-05	0.000802	7.530348	UP	centromere protein M
ILMN_2211780	SLC25A4	1.050337	1.64E-05	0.000826	7.491293	UP	solute carrier family 25 member 4
ILMN_1699476	RPE	1.072324	1.64E-05	0.000826	7.490652	UP	ribulose-5-phosphate-3-epimerase
ILMN_1912662	NA	1.058742	1.68E-05	0.000841	7.469251	UP	NA
ILMN_1765332	TIMM10	1.089741	1.68E-05	0.000843	7.466679	UP	translocase of inner mitochondrial membrane 10
ILMN_1779264	PSMG1	1.193296	1.71E-05	0.00085	7.452232	UP	proteasome assembly chaperone 1
ILMN_1658607	DLEU2L	1.077523	1.72E-05	0.000852	7.447551	UP	deleted in lymphocytic leukemia 2-like
ILMN_2044617	MTERF3	1.074365	1.78E-05	0.000867	7.422457	UP	mitochondrial transcription termination factor 3
ILMN_1776586	RPL26L1	1.022555	1.80E-05	0.000876	7.412239	UP	ribosomal protein L26 like 1
ILMN_1669940	TMEM38B	1.210727	1.85E-05	0.00089	7.388717	UP	transmembrane protein 38B
ILMN_3256471	FAM87B	1.178361	1.91E-05	0.000911	7.360085	UP	family with sequence similarity 87 member B
ILMN_3243851	GAGE12D	1.552344	1.92E-05	0.000912	7.357524	UP	G antigen 12D
ILMN_1775170	MT1X	2.348193	2.05E-05	0.000956	7.303241	UP	metallothionein 1X
ILMN_1760635	RAD51C	1.127283	2.07E-05	0.00096	7.297171	UP	RAD51 paralog C
ILMN_2173611	MT1E	1.73121	2.12E-05	0.000973	7.275216	UP	metallothionein 1E
ILMN_3310351	RNU6-33P	1.492367	2.12E-05	0.000973	7.274225	UP	RNA, U6 small nuclear 33, pseudogene
ILMN_1802557	HEBP1	1.141586	2.14E-05	0.000976	7.269221	UP	heme binding protein 1
ILMN_3243859	NDUFB9	1.298753	2.14E-05	0.000977	7.266336	UP	NADH:ubiquinoneoxidoreductase subunit B9
ILMN_1803775	NA	1.179177	2.18E-05	0.000988	7.251772	UP	NA
ILMN_1682935	LYPLAL1	1.099385	2.22E-05	0.000997	7.239552	UP	lysophospholipase like 1
ILMN_1758106	NA	-2.96765	4.03E-12	9.70E-08	-34.6661	Down	NA
ILMN_2411781	RYR1	-3.06977	1.25E-11	2.01E-07	-31.074	Down	ryanodine receptor 1
ILMN_1706505	COL5A1	-2.70633	4.32E-10	3.64E-06	-22.0264	Down	collagen type V alpha 1 chain
ILMN_1664912	IL11RA	-1.61102	5.62E-10	3.64E-06	-21.4654	Down	interleukin 11 receptor subunit alpha
ILMN_1678353	FARP1	-1.83982	2.32E-09	1.01E-05	-18.6784	Down	FERM, ARH/RhoGEF and pleckstrin domain protein 1
ILMN_1699676	SPTSSA	-1.97044	2.58E-09	1.01E-05	-18.4875	Down	serine palmitoyltransferase small subunit A
ILMN_1679391	MAMDC2	-2.86794	2.70E-09	1.01E-05	-18.4053	Down	MAM domain containing 2
ILMN_1800697	LDB2	-2.72827	2.72E-09	1.01E-05	-18.3893	Down	LIM domain binding 2
ILMN_1704353	IGSF3	-1.92826	4.46E-09	1.43E-05	-17.5146	Down	immunoglobulin superfamily member 3
ILMN_1767934	PCSK5	-1.76304	6.00E-09	1.59E-05	-17.008	Down	proproteinconvertasesubtilisin/kexin type 5
ILMN_3259146	BST2	-1.39969	6.64E-09	1.59E-05	-16.8384	Down	bone marrow stromal cell antigen 2
ILMN_1771987	SLC44A2	-1.48973	6.91E-09	1.59E-05	-16.7717	Down	solute carrier family 44 member 2
ILMN_1687921	JMJD8	-1.45577	6.92E-09	1.59E-05	-16.7687	Down	jumonji domain containing 8
ILMN_1779558	GAS6	-2.24874	7.48E-09	1.64E-05	-16.6406	Down	growth arrest specific 6
ILMN_3203444	CERCAM	-1.8836	8.72E-09	1.68E-05	-16.3899	Down	cerebral endothelial cell adhesion molecule
ILMN_1760778	ENG	-1.39429	9.44E-09	1.68E-05	-16.2614	Down	endoglin
ILMN_1695290	FERMT2	-1.5428	9.81E-09	1.68E-05	-16.1996	Down	fermitin family member 2
ILMN_1675656	PPFIBP2	-1.99518	1.07E-08	1.73E-05	-16.0629	Down	PPFIA binding protein 2
ILMN_1756992	MUC1	-1.78241	1.17E-08	1.81E-05	-15.9194	Down	mucin 1, cell surface associated
ILMN_1789627	SEPT5	-2.057	1.44E-08	2.10E-05	-15.5897	Down	septin 5
ILMN_1729117	COL5A2	-2.80181	1.50E-08	2.12E-05	-15.527	Down	collagen type V alpha 2 chain
ILMN_1738742	PLAT	-2.92228	1.58E-08	2.15E-05	-15.4514	Down	plasminogen activator, tissue type
ILMN_1758731	CYP2J2	-1.6795	2.06E-08	2.67E-05	-15.042	Down	cytochrome P450 family 2 subfamily J member 2
ILMN_3249366	JMJD8	-1.37258	2.11E-08	2.67E-05	-15.0073	Down	jumonji domain containing 8
ILMN_1784661	TMEM2	-1.92934	2.35E-08	2.72E-05	-14.8473	Down	transmembrane protein 2
ILMN_2352131	ERBB2	-1.13695	2.37E-08	2.72E-05	-14.8355	Down	erb-b2 receptor tyrosine kinase 2
ILMN_1653711	FZD2	-1.70334	2.43E-08	2.72E-05	-14.7999	Down	frizzled class receptor 2
ILMN_2388547	EPSTI1	-1.43776	2.52E-08	2.72E-05	-14.7479	Down	epithelial stromal interaction 1
ILMN_2104356	COL1A2	-2.99013	2.64E-08	2.76E-05	-14.676	Down	collagen type I alpha 2 chain
ILMN_1690839	PLPP7	-1.57925	2.83E-08	2.87E-05	-14.5761	Down	phospholipid phosphatase 7 (inactive)
ILMN_1715169	HLA-DRB1	-1.63269	2.86E-08	2.87E-05	-14.5578	Down	major histocompatibility complex, class II, DR beta 1
ILMN_1776157	SEPT4	-1.66175	3.10E-08	2.96E-05	-14.4414	Down	septin 4
ILMN_1721283	HSPB6	-1.81144	3.13E-08	2.96E-05	-14.4275	Down	heat shock protein family B (small) member 6
ILMN_1788250	LDOC1	-1.19399	3.34E-08	3.04E-05	-14.3336	Down	LDOC1, regulator of NFKB signaling
ILMN_1674908	HOXB5	-1.06009	3.35E-08	3.04E-05	-14.3301	Down	homeobox B5
ILMN_1703178	SCG2	-3.72906	3.47E-08	3.09E-05	-14.2793	Down	secretogranin II
ILMN_1748323	CXCL14	-2.04525	4.24E-08	3.51E-05	-13.9978	Down	C-X-C motif chemokine ligand 14
ILMN_1772692	DICER1	-1.37828	4.31E-08	3.51E-05	-13.9731	Down	dicer 1, ribonuclease III
ILMN_1805842	FHL1	-1.31901	4.41E-08	3.53E-05	-13.9414	Down	four and a half LIM domains 1
ILMN_1676213	SRPX2	-1.70434	4.88E-08	3.79E-05	-13.8013	Down	sushi repeat containing protein, X-linked 2
ILMN_1665831	CLPTM1	-1.82184	5.44E-08	4.03E-05	-13.649	Down	CLPTM1, transmembrane protein
ILMN_2066060	HLA-DRB6	-1.27999	6.33E-08	4.29E-05	-13.4433	Down	major histocompatibility complex, class II, DR beta 6 (pseudogene)
ILMN_1669709	TMEM108	-1.58478	6.37E-08	4.29E-05	-13.4339	Down	transmembrane protein 108
ILMN_1809928	COL6A2	-1.73792	6.38E-08	4.29E-05	-13.4333	Down	collagen type VI alpha 2 chain
ILMN_1746158	HOXD11	-1.74893	6.43E-08	4.29E-05	-13.4228	Down	homeobox D11
ILMN_1682062	RYR1	-1.98541	6.68E-08	4.29E-05	-13.3694	Down	ryanodine receptor 1
ILMN_2401978	STAT3	-1.46266	6.70E-08	4.29E-05	-13.3672	Down	signal transducer and activator of transcription 3
ILMN_2340052	NCOR2	-1.07118	6.88E-08	4.31E-05	-13.3309	Down	nuclear receptor corepressor 2
ILMN_1746888	PCOLCE2	-1.51317	7.14E-08	4.38E-05	-13.2811	Down	procollagen C-endopeptidase enhancer 2
ILMN_2370624	COL13A1	-1.99766	7.19E-08	4.38E-05	-13.2712	Down	collagen type XIII alpha 1 chain

ILMN_2086470	PDGFRA	-1.95344	7.44E-08	4.47E-05	-13.2261	Down	platelet derived growth factor receptor alpha
ILMN_1784749	GAS6	-2.18245	8.29E-08	4.59E-05	-13.0819	Down	growth arrest specific 6
ILMN_2380237	C1QTNF1	-1.61813	8.39E-08	4.59E-05	-13.0656	Down	C1q and TNF related 1
ILMN_3226214	LOC728755	-2.19084	8.44E-08	4.59E-05	-13.0576	Down	uncharacterized LOC728755
ILMN_3240222	PRAG1	-1.26144	8.44E-08	4.59E-05	-13.0574	Down	PEAK1 related kinase activating pseudokinase 1
ILMN_1662932	LCP1	-1.76494	8.63E-08	4.59E-05	-13.0284	Down	lymphocyte cytosolic protein 1
ILMN_2048591	LRRN3	-1.44968	9.53E-08	4.88E-05	-12.8982	Down	leucine rich repeat neuronal 3
ILMN_1754921	FAM43B	-1.25859	9.80E-08	4.95E-05	-12.8618	Down	family with sequence similarity 43 member B
ILMN_2066066	HLA-DRB6	-1.25262	1.05E-07	5.21E-05	-12.7716	Down	major histocompatibility complex, class II, DR beta 6 (pseudogene)
ILMN_1752592	HLA-DRB4	-1.30984	1.11E-07	5.35E-05	-12.6988	Down	major histocompatibility complex, class II, DR beta 4
ILMN_2193325	MMP23B	-2.55575	1.20E-07	5.69E-05	-12.5991	Down	matrix metallopeptidase 23B
ILMN_1801068	DACT1	-1.15795	1.23E-07	5.69E-05	-12.5685	Down	dishevelled binding antagonist of beta catenin 1
ILMN_1672589	SEMA4B	-1.07458	1.23E-07	5.69E-05	-12.5682	Down	semaphorin 4B
ILMN_2132982	IGFBP5	-3.14421	1.28E-07	5.87E-05	-12.5173	Down	insulin like growth factor binding protein 5
ILMN_1815057	PDGFRB	-2.41794	1.29E-07	5.87E-05	-12.505	Down	platelet derived growth factor receptor beta
ILMN_2349831	DICER1	-1.07473	1.32E-07	5.93E-05	-12.4807	Down	dicer 1, ribonuclease III
ILMN_2389876	TGFB1I1	-1.2041	1.35E-07	5.93E-05	-12.4469	Down	transforming growth factor beta 1 induced transcript 1
ILMN_1670490	PDPN	-2.18279	1.40E-07	6.00E-05	-12.4063	Down	podoplanin
ILMN_1774387	ZHX3	-1.23772	1.44E-07	6.12E-05	-12.3717	Down	zinc fingers and homeoboxes 3
ILMN_1692535	DPP4	-1.0772	1.55E-07	6.36E-05	-12.2789	Down	dipeptidyl peptidase 4
ILMN_1785272	COL1A2	-2.58821	1.59E-07	6.42E-05	-12.2465	Down	collagen type I alpha 2 chain
ILMN_2396956	AKAP13	-1.01749	1.66E-07	6.55E-05	-12.1904	Down	A-kinase anchoring protein 13
ILMN_1808707	FSCN1	-1.22422	1.67E-07	6.55E-05	-12.1856	Down	fascin actin-bundling protein 1
ILMN_1708934	ADM	-1.93244	1.68E-07	6.55E-05	-12.1761	Down	adrenomedullin
ILMN_1695917	C5orf15	-1.25526	1.69E-07	6.55E-05	-12.1713	Down	chromosome 5 open reading frame 15
ILMN_1683576	MAGED2	-1.4592	1.80E-07	6.83E-05	-12.0878	Down	MAGE family member D2
ILMN_1723480	BST2	-1.53528	1.95E-07	7.34E-05	-11.9908	Down	bone marrow stromal cell antigen 2
ILMN_1771599	PLOD2	-1.44299	2.05E-07	7.58E-05	-11.9317	Down	procollagen-lysine,2-oxoglutarate 5-dioxygenase 2
ILMN_1779855	HSD17B6	-1.78807	2.14E-07	7.74E-05	-11.8783	Down	hydroxysteroid 17-beta dehydrogenase 6
ILMN_1738578	FILIP1L	-1.95165	2.16E-07	7.74E-05	-11.8698	Down	filamin A interacting protein 1 like
ILMN_2112493	DAP	-1.08311	2.18E-07	7.77E-05	-11.8563	Down	death associated protein
ILMN_1667893	TNS3	-1.40305	2.28E-07	7.95E-05	-11.7999	Down	tensin 3
ILMN_1813043	BHMT2	-1.12359	2.33E-07	8.01E-05	-11.7747	Down	betaine--homocysteine S-methyltransferase 2
ILMN_1774077	GBP2	-1.64656	2.43E-07	8.21E-05	-11.7239	Down	guanylate binding protein 2
ILMN_1741204	KLHDC2	-1.27356	2.49E-07	8.26E-05	-11.6966	Down	kelch domain containing 2
ILMN_1766914	MFAP4	-2.72931	2.54E-07	8.32E-05	-11.6739	Down	microfibrillar associated protein 4
ILMN_1769694	ASIC1	-1.64023	2.62E-07	8.47E-05	-11.6341	Down	acid sensing ion channel subunit 1
ILMN_2140059	LAMA4	-1.93311	2.76E-07	8.84E-05	-11.572	Down	laminin subunit alpha 4
ILMN_1769282	FRMD6	-1.75235	2.79E-07	8.84E-05	-11.5591	Down	FERM domain containing 6
ILMN_1705750	TGM2	-2.05791	2.82E-07	8.87E-05	-11.5478	Down	transglutaminase 2
ILMN_2197030	ZFYVE21	-1.06008	2.86E-07	8.92E-05	-11.5313	Down	zinc finger FYVE-type containing 21
ILMN_1755727	KDM5B	-1.44504	2.90E-07	8.95E-05	-11.5143	Down	lysine demethylase 5B
ILMN_1666502	SOBP	-1.48753	3.24E-07	9.68E-05	-11.384	Down	sine oculis binding protein homolog
ILMN_2206272	D2HGDH	-1.02891	3.28E-07	9.71E-05	-11.3689	Down	D-2-hydroxyglutarate dehydrogenase
ILMN_1801377	SLC29A4	-2.26368	3.30E-07	9.71E-05	-11.3632	Down	solute carrier family 29 member 4
ILMN_2380163	PTPRF	-2.55197	3.31E-07	9.71E-05	-11.359	Down	protein tyrosine phosphatase, receptor type F
ILMN_1750563	CERCAM	-1.78683	3.45E-07	9.82E-05	-11.3094	Down	cerebral endothelial cell adhesion molecule
ILMN_1721022	SHC1	-1.158	3.47E-07	9.82E-05	-11.3038	Down	SHC adaptor protein 1
ILMN_1796762	CCDC102A	-1.0193	3.55E-07	9.97E-05	-11.2791	Down	coiled-coil domain containing 102A
ILMN_1701308	COL1A1	-3.17663	3.68E-07	0.000102	-11.2371	Down	collagen type I alpha 1 chain
ILMN_2184966	ZHX2	-1.21965	3.91E-07	0.000105	-11.1669	Down	zinc fingers and homeoboxes 2
ILMN_1655077	PRDM1	-1.69805	3.96E-07	0.000106	-11.1528	Down	PR/SET domain 1
ILMN_1796216	VASH1	-1.22439	4.08E-07	0.000107	-11.1184	Down	vasohibin 1
ILMN_2062468	IGFBP7	-2.13765	4.10E-07	0.000107	-11.1121	Down	insulin like growth factor binding protein 7
ILMN_2394250	PLEKHA1	-1.12985	4.34E-07	0.00011	-11.0453	Down	pleckstrin homology domain containing A1
ILMN_1727135	FIBCD1	-1.36665	4.38E-07	0.00011	-11.0357	Down	fibrinogen C domain containing 1
ILMN_2228732	CCNG2	-1.00278	4.49E-07	0.000112	-11.0083	Down	cyclin G2
ILMN_1661599	DDIT4	-2.87903	4.58E-07	0.000114	-10.9843	Down	DNA damage inducible transcript 4
ILMN_1783909	COL6A2	-1.9603	4.63E-07	0.000114	-10.9728	Down	collagen type VI alpha 2 chain
ILMN_1763834	APLP1	-1.09319	5.05E-07	0.000121	-10.8752	Down	amyloid beta precursor like protein 1
ILMN_3228688	HLA-DRB1	-1.27744	5.16E-07	0.000122	-10.8509	Down	major histocompatibility complex, class II, DR beta 1
ILMN_1685433	COL8A1	-1.5761	5.60E-07	0.000128	-10.7597	Down	collagen type VIII alpha 1 chain
ILMN_2201678	FSTL1	-3.03715	5.74E-07	0.00013	-10.731	Down	follistatin like 1
ILMN_1714691	HOXD10	-1.0378	5.93E-07	0.000132	-10.6961	Down	homeobox D10
ILMN_1727778	NTNG1	-1.77157	5.94E-07	0.000132	-10.6932	Down	netrin G1
ILMN_1662340	ZNF358	-1.08083	6.12E-07	0.000134	-10.6601	Down	zinc finger protein 358
ILMN_1747281	EVI5L	-1.12823	6.19E-07	0.000135	-10.6474	Down	ecotropic viral integration site 5 like
ILMN_1771964	GSTA4	-1.22952	6.23E-07	0.000135	-10.6408	Down	glutathione S-transferase alpha 4
ILMN_1697694	ATP6AP1	-1.08422	6.24E-07	0.000135	-10.6395	Down	ATPase H+ transporting accessory protein 1
ILMN_1811238	ALPK2	-1.76924	6.47E-07	0.000138	-10.599	Down	alpha kinase 2
ILMN_2375002	MAP4K4	-1.08718	6.50E-07	0.000138	-10.5931	Down	mitogen-activated protein kinase kinasekinasekinase 4
ILMN_1803018	KIFC2	-1.08987	6.84E-07	0.000141	-10.5375	Down	kinesin family member C2
ILMN_1796734	SPARC	-1.91983	6.97E-07	0.000143	-10.5179	Down	secreted protein acidic and cysteine rich
ILMN_2061435	MEG3	-3.64387	7.23E-07	0.000147	-10.4773	Down	maternally expressed 3 (non-protein coding)

ILMN_1795442	LAMA4	-1.8627	7.24E-07	0.000147	-10.4759	Down	laminin subunit alpha 4
ILMN_3247578	FAT1	-1.94464	7.66E-07	0.000152	-10.4139	Down	FAT atypical cadherin 1
ILMN_2242463	CTSC	-1.23308	7.70E-07	0.000152	-10.4091	Down	cathepsin C
ILMN_2311052	COL13A1	-1.70898	7.70E-07	0.000152	-10.4082	Down	collagen type XIII alpha 1 chain
ILMN_2406815	LRRC17	-2.15528	7.71E-07	0.000152	-10.4066	Down	leucine rich repeat containing 17
ILMN_2410929	PAPSS2	-1.53257	7.77E-07	0.000152	-10.3991	Down	3'-phosphoadenosine 5'-phosphosulfate synthase 2
ILMN_1684497	PYROXD2	-1.30406	7.82E-07	0.000152	-10.3913	Down	pyridine nucleotide-disulphide oxidoreductase domain 2
ILMN_1729288	C1QTNF6	-1.62426	7.93E-07	0.000152	-10.3761	Down	C1q and TNF related 6
ILMN_1756541	MXD4	-1.69193	7.94E-07	0.000152	-10.3758	Down	MAX dimerization protein 4
ILMN_1684554	COL16A1	-1.44548	8.01E-07	0.000152	-10.3654	Down	collagen type XVI alpha 1 chain
ILMN_1808713	HSD17B2	-2.95221	8.21E-07	0.000154	-10.3398	Down	hydroxysteroid 17-beta dehydrogenase 2
ILMN_1778457	IL18	-1.03845	8.25E-07	0.000154	-10.334	Down	interleukin 18
ILMN_1779071	FEZ1	-1.99291	8.49E-07	0.000155	-10.3032	Down	fasciculation and elongation protein zeta 1
ILMN_1734950	LOXL1	-1.94225	8.49E-07	0.000155	-10.3032	Down	lysyl oxidase like 1
ILMN_1779171	SGSM2	-1.21166	8.51E-07	0.000155	-10.3011	Down	small G protein signaling modulator 2
ILMN_1810729	UBL3	-1.00926	8.53E-07	0.000155	-10.2978	Down	ubiquitin like 3
ILMN_2189371	NTNG1	-1.8086	8.89E-07	0.000157	-10.254	Down	netrin G1
ILMN_1791447	CXCL12	-1.45251	9.85E-07	0.000166	-10.1446	Down	C-X-C motif chemokine ligand 12
ILMN_1760688	SAMD14	-1.04169	9.88E-07	0.000166	-10.1406	Down	sterile alpha motif domain containing 14
ILMN_2313901	PAM	-1.02007	9.89E-07	0.000166	-10.14	Down	peptidylglycine alpha-amidatingmonooxygenase
ILMN_1673409	MIR503HG	-4.0338	1.01E-06	0.000168	-10.1197	Down	MIR503 host gene
ILMN_1687652	TGFB3	-2.5175	1.04E-06	0.00017	-10.0882	Down	transforming growth factor beta 3
ILMN_2401906	CDAN1	-1.15619	1.04E-06	0.000171	-10.0819	Down	codanin 1
ILMN_1742534	COL4A5	-1.39882	1.15E-06	0.000185	-9.97979	Down	collagen type IV alpha 5 chain
ILMN_1681984	GALNT10	-1.01045	1.17E-06	0.000185	-9.96269	Down	polypeptide N-acetylgalactosaminyltransferase 10
ILMN_3283032	CERCAM	-1.50136	1.21E-06	0.000188	-9.92495	Down	cerebral endothelial cell adhesion molecule
ILMN_1751656	KLF11	-1.20904	1.23E-06	0.000188	-9.9144	Down	Kruppel like factor 11
ILMN_1730906	FILIP1L	-1.58604	1.32E-06	0.000198	-9.83702	Down	filamin A interacting protein 1 like
ILMN_1698706	NID2	-1.23439	1.34E-06	0.0002	-9.82529	Down	nidogen 2
ILMN_2371911	MUC1	-1.18471	1.38E-06	0.000203	-9.79521	Down	mucin 1, cell surface associated
ILMN_2329735	ECM1	-1.46377	1.39E-06	0.000204	-9.78428	Down	extracellular matrix protein 1
ILMN_1694325	NFIX	-1.27785	1.44E-06	0.000208	-9.74941	Down	nuclear factor I X
ILMN_1695880	LOX	-1.87174	1.46E-06	0.000211	-9.73402	Down	lysyl oxidase
ILMN_1696187	PYGL	-1.43631	1.50E-06	0.000214	-9.70306	Down	glycogen phosphorylase L
ILMN_1799139	PLOD2	-1.5585	1.54E-06	0.000216	-9.67894	Down	procollagen-lysine,2-oxoglutarate 5-dioxygenase 2
ILMN_2206746	BGN	-1.99063	1.58E-06	0.00022	-9.65193	Down	biglycan
ILMN_2391150	FILIP1L	-1.37917	1.59E-06	0.00022	-9.6439	Down	filamin A interacting protein 1 like
ILMN_1794492	HOXC6	-1.1556	1.63E-06	0.000224	-9.62038	Down	homeobox C6
ILMN_1791912	SIDT2	-1.20706	1.70E-06	0.000232	-9.5775	Down	SID1 transmembrane family member 2
ILMN_2332964	LGMN	-1.38794	1.74E-06	0.000233	-9.55518	Down	legumain
ILMN_1807042	MARCKS	-1.24401	1.74E-06	0.000233	-9.5546	Down	myristoylated alanine rich protein kinase C substrate
ILMN_2326273	CHI3L2	-3.0745	1.74E-06	0.000233	-9.55279	Down	chitinase 3 like 2
ILMN_2375003	MAP4K4	-1.01579	1.76E-06	0.000234	-9.54439	Down	mitogen-activated protein kinase kinasekinasekinase 4
ILMN_1713266	FAM46C	-1.33194	1.77E-06	0.000235	-9.53612	Down	family with sequence similarity 46 member C
ILMN_1700268	QPRT	-1.47136	1.81E-06	0.000237	-9.51398	Down	quinolinatephosphoribosyltransferase
ILMN_2223903	PPIC	-1.11497	1.82E-06	0.000237	-9.50733	Down	peptidylprolylisomerase C
ILMN_3260286	PCYOX1	-1.10236	1.87E-06	0.000238	-9.48334	Down	prenylcysteine oxidase 1
ILMN_1691476	MYLK	-1.39852	1.92E-06	0.000242	-9.45803	Down	myosin light chain kinase
ILMN_2328094	DACT1	-1.01662	1.92E-06	0.000242	-9.4571	Down	dishevelled binding antagonist of beta catenin 1
ILMN_1782004	NKX3-2	-1.28472	1.93E-06	0.000242	-9.45299	Down	NK3 homeobox 2
ILMN_2309180	SMARCD3	-1.18462	1.95E-06	0.000243	-9.43895	Down	SWI/SNF related, matrix associated, actin dependent regulator of chromatin, subfamily d, member 3
ILMN_1688722	IL13RA2	-1.99282	1.95E-06	0.000243	-9.43811	Down	interleukin 13 receptor subunit alpha 2
ILMN_2150654	ZSWIM4	-1.53559	1.98E-06	0.000243	-9.42645	Down	zinc finger SWIM-type containing 4
ILMN_2086890	ANGPT1	-1.09416	2.04E-06	0.000248	-9.39325	Down	angiopoietin 1
ILMN_1677814	ABCC3	-1.38335	2.06E-06	0.00025	-9.38352	Down	ATP binding cassette subfamily C member 3
ILMN_1698019	LGMN	-1.53774	2.09E-06	0.000251	-9.37036	Down	legumain
ILMN_1653432	HNRNPDL	-2.0476	2.11E-06	0.000252	-9.36177	Down	heterogeneous nuclear ribonucleoprotein D like
ILMN_1677723	ANGPT1	-1.301	2.15E-06	0.000254	-9.3407	Down	angiopoietin 1
ILMN_3242038	GPX8	-1.36884	2.16E-06	0.000254	-9.33816	Down	glutathione peroxidase 8 (putative)
ILMN_1743187	C6orf120	-1.08701	2.18E-06	0.000254	-9.33144	Down	chromosome 6 open reading frame 120
ILMN_1670272	LRP10	-1.09028	2.18E-06	0.000254	-9.32937	Down	LDL receptor related protein 10
ILMN_1738866	DEXI	-1.05358	2.18E-06	0.000254	-9.32877	Down	Dexi homolog
ILMN_2388507	AKT1	-1.06722	2.27E-06	0.000258	-9.29135	Down	AKT serine/threonine kinase 1
ILMN_1693334	P4HA1	-1.70127	2.29E-06	0.00026	-9.27931	Down	prolyl 4-hydroxylase subunit alpha 1
ILMN_1709044	TGIF2	-1.12673	2.36E-06	0.000263	-9.25002	Down	TGFB induced factor homeobox 2
ILMN_1696347	CTSC	-1.00086	2.38E-06	0.000264	-9.24208	Down	cathepsin C
ILMN_2410909	AKT1	-1.19671	2.47E-06	0.00027	-9.20676	Down	AKT serine/threonine kinase 1
ILMN_1744963	ERO1A	-1.4567	2.52E-06	0.000272	-9.18708	Down	endoplasmic reticulum oxidoreductase 1 alpha
ILMN_1812096	CADM4	-1.31623	2.53E-06	0.000272	-9.18426	Down	cell adhesion molecule 4
ILMN_2206722	FER1L4	-1.94284	2.56E-06	0.000276	-9.16996	Down	fer-1 like family member 4, pseudogene
ILMN_1743130	PTGFRN	-1.30732	2.77E-06	0.000292	-9.09353	Down	prostaglandin F2 receptor inhibitor
ILMN_3240226	LOC155060	-1.45605	2.86E-06	0.000296	-9.06157	Down	AI894139 pseudogene
ILMN_1750324	IGFBP5	-3.3124	3.03E-06	0.000305	-9.00882	Down	insulin like growth factor binding protein 5
ILMN_1752968	LAMB2	-1.75955	3.08E-06	0.00031	-8.99021	Down	laminin subunit beta 2

ILMN_1662839	PLEKHA1	-1.33847	3.12E-06	0.000312	-8.97977	Down	pleckstrin homology domain containing A1
ILMN_1718285	HOXC8	-1.83901	3.21E-06	0.000318	-8.95249	Down	homeobox C8
ILMN_1732151	COL6A1	-1.73077	3.22E-06	0.000318	-8.9479	Down	collagen type VI alpha 1 chain
ILMN_2329914	SPRY1	-1.96464	3.25E-06	0.00032	-8.93853	Down	sprouty RTK signaling antagonist 1
ILMN_1750093	NA	-1.11886	3.32E-06	0.000322	-8.91817	Down	NA
ILMN_1728180	LUC7L3	-1.7839	3.37E-06	0.000324	-8.90437	Down	LUC7 like 3 pre-mRNA splicing factor
ILMN_1754795	FAT1	-1.89037	3.41E-06	0.000326	-8.89446	Down	FAT atypical cadherin 1
ILMN_1720235	ADSSL1	-1.98793	3.41E-06	0.000326	-8.89407	Down	adenylosuccinate synthase like 1
ILMN_1751161	COL7A1	-1.53991	3.45E-06	0.000328	-8.88219	Down	collagen type VII alpha 1 chain
ILMN_1703041	IDUA	-1.28978	3.52E-06	0.000333	-8.86403	Down	iduronidase, alpha-L-
ILMN_1778401	HLA-B	-1.74522	3.59E-06	0.000336	-8.84614	Down	major histocompatibility complex, class I, B
ILMN_1800412	BMP1	-1.27568	3.74E-06	0.000346	-8.80603	Down	bone morphogenetic protein 1
ILMN_3245413	DENND5A	-1.23926	3.82E-06	0.000347	-8.78697	Down	DENN domain containing 5A
ILMN_2067269	RECK	-1.40029	3.86E-06	0.000347	-8.77532	Down	reversion inducing cysteine rich protein with kazal motifs
ILMN_1685854	IGIP	-1.77358	4.01E-06	0.000358	-8.74104	Down	IgA inducing protein
ILMN_2368773	FAM3C	-1.1288	4.03E-06	0.000359	-8.73469	Down	family with sequence similarity 3 member C
ILMN_1805750	IFITM3	-2.40986	4.12E-06	0.000364	-8.71433	Down	interferon induced transmembrane protein 3
ILMN_1804735	CBS	-2.12162	4.22E-06	0.00037	-8.6924	Down	cystathionine-beta-synthase
ILMN_1785356	DENND5A	-1.30603	4.23E-06	0.00037	-8.68963	Down	DENN domain containing 5A
ILMN_1673352	IFITM2	-2.38382	4.31E-06	0.000375	-8.67255	Down	interferon induced transmembrane protein 2
ILMN_2274775	SLC44A4	-1.02479	4.42E-06	0.000383	-8.64945	Down	solute carrier family 44 member 4
ILMN_1656233	SPDYE7P	-1.1121	4.47E-06	0.000385	-8.63931	Down	speedy/RINGO cell cycle regulator family member E7, pseudogene
ILMN_2360784	RRBP1	-1.13698	4.56E-06	0.00039	-8.61992	Down	ribosome binding protein 1
ILMN_2402392	COL8A1	-1.42312	4.57E-06	0.00039	-8.6177	Down	collagen type VIII alpha 1 chain
ILMN_1777261	FAM3C	-1.20661	4.63E-06	0.000393	-8.60627	Down	family with sequence similarity 3 member C
ILMN_2157277	AKR1D1	-1.00208	4.71E-06	0.000397	-8.58914	Down	aldo-ketoreductase family 1 member D1
ILMN_1663490	ZNF541	-1.22192	4.75E-06	0.000399	-8.58203	Down	zinc finger protein 541
ILMN_1785252	SLC26A6	-1.02224	4.80E-06	0.000402	-8.57323	Down	solute carrier family 26 member 6
ILMN_1729980	RNF216	-1.19391	4.86E-06	0.000405	-8.56033	Down	ring finger protein 216
ILMN_1769633	CTSO	-1.17879	4.97E-06	0.000409	-8.53994	Down	cathepsin O
ILMN_1678215	RHOJ	-1.42541	5.05E-06	0.000414	-8.52494	Down	ras homolog family member J
ILMN_1720048	CCL2	-3.56374	5.10E-06	0.000416	-8.51616	Down	C-C motif chemokine ligand 2
ILMN_1682658	EPM2AIP1	-1.107	5.21E-06	0.000422	-8.49726	Down	EPM2A interacting protein 1
ILMN_1814204	NA	-1.29035	5.28E-06	0.000424	-8.4842	Down	NA
ILMN_2230025	PDLIM3	-1.24402	5.35E-06	0.000426	-8.47212	Down	PDZ and LIM domain 3
ILMN_1722898	SFRP2	-1.78984	5.36E-06	0.000426	-8.47031	Down	secreted frizzled related protein 2
ILMN_1747968	RBM33	-1.07894	5.38E-06	0.000426	-8.46696	Down	RNA binding motif protein 33
ILMN_2148913	TMEM45A	-1.51232	5.45E-06	0.000429	-8.45557	Down	transmembrane protein 45A
ILMN_1814710	PCDHB5	-1.07952	5.53E-06	0.000434	-8.44204	Down	protocadherin beta 5
ILMN_2330787	FRMD6	-1.45336	5.56E-06	0.000435	-8.43635	Down	FERM domain containing 6
ILMN_1709334	TM9SF1	-1.02885	5.60E-06	0.000436	-8.43053	Down	transmembrane 9 superfamily member 1
ILMN_1719986	PIK3IP1	-1.09946	5.64E-06	0.000439	-8.42299	Down	phosphoinositide-3-kinase interacting protein 1
ILMN_1656868	NPIPB4	-1.00465	5.70E-06	0.000441	-8.41371	Down	nuclear pore complex interacting protein family member B4
ILMN_1732923	SIPA1L2	-1.30516	5.71E-06	0.000441	-8.41198	Down	signal induced proliferation associated 1 like 2
ILMN_1658835	CAV2	-1.4338	5.73E-06	0.000441	-8.40892	Down	caveolin 2
ILMN_1703374	NAV1	-1.37105	5.78E-06	0.000442	-8.40166	Down	neuron navigator 1
ILMN_1724194	NPEPL1	-1.02588	5.80E-06	0.000443	-8.39784	Down	aminopeptidase-like 1
ILMN_1658356	PAMR1	-1.53077	6.09E-06	0.000459	-8.35411	Down	peptidase domain containing associated with muscle regeneration 1
ILMN_1886493	HNRNPA2B1	-1.4144	6.33E-06	0.000468	-8.31897	Down	heterogeneous nuclear ribonucleoprotein A2/B1
ILMN_1801043	GSN	-1.00426	6.37E-06	0.000469	-8.31262	Down	gelsolin
ILMN_2203588	MYL5	-1.05078	6.38E-06	0.000469	-8.31118	Down	myosin light chain 5
ILMN_1685441	ASAP3	-1.39685	6.39E-06	0.000469	-8.31061	Down	ArfGAP with SH3 domain, ankyrin repeat and PH domain 3
ILMN_1772612	ANGPTL2	-2.30564	6.43E-06	0.000472	-8.30389	Down	angiopoietin like 2
ILMN_1770290	CNN2	-1.03719	6.84E-06	0.000484	-8.24875	Down	calponin 2
ILMN_1719972	PLXNA3	-1.15609	6.90E-06	0.000487	-8.24103	Down	plexin A3
ILMN_1768754	PILRB	-1.27148	6.97E-06	0.000491	-8.23191	Down	paired immunoglobin-like type 2 receptor beta
ILMN_2077905	PTGFRN	-1.18815	7.08E-06	0.000495	-8.21761	Down	prostaglandin F2 receptor inhibitor
ILMN_1691290	CELSR3	-1.19446	7.10E-06	0.000495	-8.21499	Down	cadherin EGF LAG seven-pass G-type receptor 3
ILMN_1783840	RFX8	-1.68308	7.11E-06	0.000495	-8.21345	Down	RFX family member 8, lacking RFX DNA binding domain
ILMN_2085862	SLC15A3	-1.10441	7.14E-06	0.000496	-8.20996	Down	solute carrier family 15 member 3
ILMN_1703316	INAFM1	-1.16299	7.17E-06	0.000497	-8.20685	Down	InaF motif containing 1
ILMN_1658333	ECM1	-1.32688	7.31E-06	0.000503	-8.18955	Down	extracellular matrix protein 1
ILMN_1656057	PLAU	-1.61088	7.33E-06	0.000504	-8.18642	Down	plasminogen activator, urokinase
ILMN_1712684	FAM20C	-2.03273	7.35E-06	0.000505	-8.1842	Down	FAM20C, golgi associated secretory pathway kinase
ILMN_1829845	NA	-1.63435	7.44E-06	0.000507	-8.17341	Down	NA
ILMN_1712026	NLRP3	-1.05051	7.49E-06	0.000509	-8.16677	Down	NLR family pyrin domain containing 3
ILMN_3224934	PNISR	-1.41205	7.50E-06	0.000509	-8.16633	Down	PNN interacting serine and arginine rich protein
ILMN_1768940	COL15A1	-2.68161	7.64E-06	0.000515	-8.14908	Down	collagen type XV alpha 1 chain
ILMN_3183544	NA	-1.13196	7.86E-06	0.000525	-8.12397	Down	NA
ILMN_1684984	MAGED2	-1.39451	7.87E-06	0.000525	-8.12345	Down	MAGE family member D2
ILMN_1761277	PDE4C	-1.20539	7.89E-06	0.000525	-8.12128	Down	phosphodiesterase 4C
ILMN_1680738	NREP	-2.06102	7.92E-06	0.000526	-8.11766	Down	neuronal regeneration related protein
ILMN_1697491	PRR5L	-1.13169	7.94E-06	0.000527	-8.11527	Down	proline rich 5 like

ILMN_1789196	TPM2	-1.60385	8.01E-06	0.000529	-8.10742	Down	tropomyosin 2 (beta)
ILMN_1701613	RARRES3	-1.33366	8.17E-06	0.000538	-8.08974	Down	retinoic acid receptor responder 3
ILMN_3218292	PAXIP1-AS1	-1.3383	8.20E-06	0.000538	-8.08712	Down	PAXIP1 antisense RNA 1 (head to head)
ILMN_1713732	ABL1	-1.06801	8.53E-06	0.000552	-8.05203	Down	ABL proto-oncogene 1, non-receptor tyrosine kinase
ILMN_3307877	C21orf58	-1.35545	8.55E-06	0.000552	-8.04926	Down	chromosome 21 open reading frame 58
ILMN_2361862	VLDLR	-1.0087	8.57E-06	0.000552	-8.048	Down	very low density lipoprotein receptor
ILMN_3304111	NPIPB2	-1.17488	8.57E-06	0.000552	-8.04763	Down	nuclear pore complex interacting protein family, member B2
ILMN_1719343	WDR26	-1.45498	8.57E-06	0.000552	-8.04739	Down	WD repeat domain 26
ILMN_1664294	P3H1	-1.5344	8.63E-06	0.000554	-8.04129	Down	prolyl 3-hydroxylase 1
ILMN_1775486	SSPN	-1.18154	8.69E-06	0.000557	-8.03504	Down	sarcospan
ILMN_1751028	SERPINH1	-1.62732	8.79E-06	0.000561	-8.02535	Down	serpin family H member 1
ILMN_1728605	TTC3	-1.21427	9.00E-06	0.000571	-8.00457	Down	tetratricopeptide repeat domain 3
ILMN_1726928	TCEA3	-2.09151	9.03E-06	0.000572	-8.00156	Down	transcription elongation factor A3
ILMN_1790052	NA	-1.23779	9.06E-06	0.000572	-7.99862	Down	NA
ILMN_1752728	FUCA1	-1.00782	9.10E-06	0.000573	-7.99435	Down	fucosidase, alpha-L- 1, tissue
ILMN_1703650	TNIP1	-1.31515	9.27E-06	0.000575	-7.97875	Down	TNFAIP3 interacting protein 1
ILMN_1688630	RECK	-1.13307	9.39E-06	0.000577	-7.96694	Down	reversion inducing cysteine rich protein with kazal motifs
ILMN_1762204	GCSAML	-1.39961	9.53E-06	0.000584	-7.95363	Down	germinal center associated signaling and motility like
ILMN_1654773	CASK	-1.06603	1.05E-05	0.000622	-7.86872	Down	calcium/calmodulin dependent serine protein kinase
ILMN_1671843	PSRC1	-1.08454	1.06E-05	0.000624	-7.86264	Down	proline and serine rich coiled-coil 1
ILMN_1705144	ULK1	-1.26604	1.08E-05	0.000632	-7.84233	Down	unc-51 like autophagy activating kinase 1
ILMN_1760727	ANG	-1.34559	1.09E-05	0.000635	-7.83571	Down	angiogenin
ILMN_1772991	CHPF2	-1.0736	1.10E-05	0.000639	-7.82856	Down	chondroitin polymerizing factor 2
ILMN_1707070	PCOLCE	-1.18462	1.13E-05	0.00065	-7.80505	Down	procollagen C-endopeptidase enhancer
ILMN_1773567	LAMA5	-1.09467	1.13E-05	0.00065	-7.80337	Down	laminin subunit alpha 5
ILMN_1657373	P3H2	-2.23731	1.15E-05	0.000657	-7.78793	Down	prolyl 3-hydroxylase 2
ILMN_2112638	SVEP1	-2.77659	1.17E-05	0.000664	-7.77221	Down	sushi, von Willebrand factor type A, EGF and pentraxin domain containing 1
ILMN_2232177	ACTN1	-1.36238	1.21E-05	0.000673	-7.75036	Down	actinin alpha 1
ILMN_1772492	SSTR2	-1.29433	1.21E-05	0.000673	-7.74993	Down	somatostatin receptor 2
ILMN_2406132	LILRB3	-1.0423	1.21E-05	0.000673	-7.7496	Down	leukocyte immunoglobulin like receptor B3
ILMN_1663618	STAT3	-1.27194	1.24E-05	0.000686	-7.72854	Down	signal transducer and activator of transcription 3
ILMN_2180519	LOC729603	-1.16289	1.26E-05	0.000691	-7.71357	Down	calcineurin like EF-hand protein 1 pseudogene
ILMN_1903568	CNIH1	-1.43774	1.27E-05	0.000698	-7.70214	Down	cornichon family AMPA receptor auxiliary protein 1
ILMN_1716687	TPM1	-1.05495	1.28E-05	0.0007	-7.69673	Down	tropomyosin 1 (alpha)
ILMN_1798581	MCM8	-1.24992	1.31E-05	0.00071	-7.68003	Down	minichromosome maintenance 8 homologous recombination repair factor
ILMN_2079786	NUAK1	-1.27415	1.32E-05	0.000712	-7.67444	Down	NUAK family kinase 1
ILMN_1757467	H1F0	-1.4804	1.34E-05	0.000722	-7.65761	Down	H1 histone family member 0
ILMN_1764364	FAM19A3	-1.27222	1.36E-05	0.000725	-7.64996	Down	family with sequence similarity 19 member A3, C-C motif chemokine like
ILMN_1760556	RSRP1	-1.13349	1.36E-05	0.000725	-7.64933	Down	arginine and serine rich protein 1
ILMN_1754969	LMCD1	-2.52042	1.37E-05	0.00073	-7.64005	Down	LIM and cysteine rich domains 1
ILMN_1668194	LMTK3	-1.53018	1.42E-05	0.000752	-7.61112	Down	lemur tyrosine kinase 3
ILMN_1652826	LRRC17	-2.73147	1.45E-05	0.000764	-7.59439	Down	leucine rich repeat containing 17
ILMN_1671478	CKB	-1.27623	1.45E-05	0.000764	-7.59433	Down	creatine kinase B
ILMN_1789733	CLIP3	-1.13178	1.48E-05	0.000777	-7.57636	Down	CAP-Gly domain containing linker protein 3
ILMN_1759792	CLIP4	-1.13625	1.48E-05	0.000777	-7.57507	Down	CAP-Gly domain containing linker protein family member 4
ILMN_2179778	PHLDB2	-1.15245	1.49E-05	0.000779	-7.57054	Down	pleckstrin homology like domain family B member 2
ILMN_1789599	NBL1	-1.40328	1.49E-05	0.000779	-7.56729	Down	neuroblastoma 1, DAN family BMP antagonist
ILMN_1765310	TCEAL2	-1.26567	1.52E-05	0.000788	-7.55354	Down	transcription elongation factor A like 2
ILMN_1831106	LINC02381	-1.68168	1.52E-05	0.000788	-7.55296	Down	long intergenic non-protein coding RNA 2381
ILMN_1731374	CPE	-1.53323	1.55E-05	0.0008	-7.53411	Down	carboxypeptidase E
ILMN_1718182	THRA	-1.45599	1.56E-05	0.000802	-7.52815	Down	thyroid hormone receptor, alpha
ILMN_2261519	AIRE	-1.15173	1.57E-05	0.000802	-7.52695	Down	autoimmune regulator
ILMN_1733851	DACT3	-1.35302	1.64E-05	0.000827	-7.48852	Down	dishevelled binding antagonist of beta catenin 3
ILMN_1695025	CD2	-1.18779	1.65E-05	0.000832	-7.48157	Down	CD2 molecule
ILMN_2073307	IL10	-1.2727	1.70E-05	0.000848	-7.45648	Down	interleukin 10
ILMN_3232282	AZFP	-1.20437	1.75E-05	0.00086	-7.43635	Down	AML-associated zinc finger protein
ILMN_1792679	ITGA5	-1.11827	1.75E-05	0.000861	-7.43457	Down	integrin subunit alpha 5
ILMN_3236377	C2orf69	-1.09379	1.76E-05	0.000864	-7.42998	Down	chromosome 2 open reading frame 69
ILMN_1678842	THBS2	-1.91055	1.77E-05	0.000866	-7.42498	Down	thrombospondin 2
ILMN_2237428	SCD5	-1.14409	1.77E-05	0.000867	-7.42323	Down	stearoyl-CoA desaturase 5
ILMN_1785424	ABLIM1	-1.3418	1.80E-05	0.000877	-7.40999	Down	actin binding LIM protein 1
ILMN_2097410	DAPP1	-1.05825	1.84E-05	0.000889	-7.39271	Down	dual adaptor of phosphotyrosine and 3-phosphoinositides 1
ILMN_1766499	HSPA2	-1.58311	1.84E-05	0.000889	-7.39201	Down	heat shock protein family A (Hsp70) member 2
ILMN_3187612	FAM126B	-1.25656	1.89E-05	0.000902	-7.372	Down	family with sequence similarity 126 member B
ILMN_1738075	CMIP	-1.62575	1.91E-05	0.00091	-7.36255	Down	c-Maf inducing protein
ILMN_1684158	GPT2	-1.36418	1.92E-05	0.000912	-7.35764	Down	glutamic--pyruvic transaminase 2
ILMN_2222880	SLC25A42	-1.08987	2.00E-05	0.000939	-7.3251	Down	solute carrier family 25 member 42

ILMN_2410924	PLOD2	-1.33473	2.04E-05	0.000953	-7.30782	Down	procollagen-lysine,2-oxoglutarate 5-dioxygenase 2
ILMN_1658560	NA	-1.17154	2.04E-05	0.000953	-7.30773	Down	NA
ILMN_1690125	PDLIM7	-1.17193	2.07E-05	0.00096	-7.29411	Down	PDZ and LIM domain 7
ILMN_1735052	ULK1	-1.17529	2.09E-05	0.000962	-7.2893	Down	unc-51 like autophagy activating kinase 1
ILMN_1778668	TAGLN	-2.70343	2.09E-05	0.000962	-7.28715	Down	transgelin
ILMN_2147435	MAN2A1	-1.13102	2.10E-05	0.000965	-7.28411	Down	mannosidase alpha class 2A member 1
ILMN_3245600	LRRC37BP1	-1.20223	2.19E-05	0.000988	-7.25042	Down	leucine rich repeat containing 37B pseudogene 1
ILMN_1707434	SLC25A37	-1.51479	2.19E-05	0.000988	-7.24958	Down	solute carrier family 25 member 37
ILMN_1701052	TUBG2	-1.38593	2.19E-05	0.000988	-7.24939	Down	tubulin gamma 2

NA-Not Annotated

Table 2 The enriched pathway terms of the up-regulated differentially expressed genes

Kyoto Encyclopedia of Genes and Genomes (KEGG), GenMAPP(MAP) and Pathway Ontology (PW)

Table 3 The enriched pathway terms of the down-regulated differentially expressed genes

Pathway ID	Pathway Name	P-value	FDR B&H	FDR B&Y	Bonferroni	Gene Count	Gene
**KEGG**							
83036	Ribosome	4.419E-8	7.203E-6	4.087E-5	7.203E-6	12 / 154	RPL34,RPL41,RPL36A, RPLP0,RPS7,MRPL30, RPS24,MRPL35,MRPS17 , MRPL20, RPL14 , RPL26L1
**KEGG**							
**Pathway ID**	**Pathway Name**	**P-value**	**FDR B&H**	**FDR B&Y**	**Bonferroni**	**Gene Count**	**Gene**
212237	Mineral absorption	1.528E-4	1.246E-2	7.068E-2	2.491E-2	5/51	MT1E,MT1F,MT1H, MT1M,MT1X
82998	Pyruvate metabolism	6.069E-4	3.298E-2	1.871E-1	9.893E-2	4/39	ACAT2,ME2,GLO1,LDHB
**REACTOME**							
1268845	Mitochondrial translation termination	1.115E-9	5.900E-7	4.041E-6	5.900E-7	11/88	MRPS31,MRPL30, MRPL35,MRPS30, MRPS17,MRRF, MRPL20,MRPL58, MRPL39,MRPS23, MRPL40
1268842	Mitochondrial translation	2.565E-9	6.783E-7	4.646E-6	1.357E-6	11/95	MRPS31,MRPL30, MRPL35,MRPS30, MRPS17,MRRF, MRPL20,MRPL58, MRPL39,MRPS23, MRPL40

Pathway ID	Pathway Name	P-value	FDR B&H	FDR B&Y	Bonferroni	Gene Count	Gene
83067	Focal adhesion	3.753E-10	1.036E-7	6.421E-7	1.036E-7	19/199	ERBB2,MYL5, MYLK, PDGFRA, PDGFRB, ACTN1,ITGA5,AKT1, COL1A1,COL1A2,COL4A5, COL6A1,COL6A2,SHC1,LAMA4,LAMA5,LAMB2, CAV2 , THBS2
172847	Protein digestion and absorption	1.734E-7	2.393E-5	1.484E-4	4.787E-5	11/90	COL1A1,COL1A2,COL4A5,COL5A1,COL5A2,DPP4,COL6A1, COL6A2,COL7A1,COL13A1,COL15A1
83068	ECM-receptor interaction	6.526E-7	6.004E-5	3.722E-4	1.801E-4	10/82	ITGA5,COL1A1,COL1A2,COL4A5,COL6A1,LAMA4,LAMA5, LAMB2,THBS2
169642	Toxoplasmosis	1.236E-5	8.529E-4	5.287E-3	3.412E-3	10/113	IL10,HLA-DRB1,HLA-DRB4,STAT3,AKT1,HSPA2,LAMA4,LAMA5,LAMB2,TGFB3
167324	Amoebiasis	2.133E-5	1.177E-3	7.299E-3	5.887E-3	9/96	IL10,ACTN1,COL1A1,COL1A2,COL4A5,LAMA4,LAMA5,LAMB2,TGFB3

**Pathway Interaction Database**							
137996	VEGFR3 signaling in lymphatic endothelium	4.335E-4	2.614E-2	1.379E-1	4.726E-2	4/23	ITGA5,AKT1,COL1A1, COL1A2
137933	IL4-mediated signaling events	4.797E-4	2.614E-2	1.379E-1	5.229E-2	6/63	IL10,IL13RA2,AKT1,COL1A1, COL1A2,SHC1
**REACTOME**							
**GenMAPP**							
MAP00620	Pyruvate metabolism	3.617E-3	4.702E-2	1.495E-1	4.702E-2	3/31	ME2,GLO1,LDHB
**Pathway Ontology**							
PW:0000034	oxidative phosphorylation	1.807E-3	3.072E-2	1.057E-1	3.072E-2	4/52	NDUFA9, NDUFB9, ATP6V0D2, COX5B

1268843	Mitochondrial translation initiation	1.687E-8	2.493E-6	1.707E-5	8.925E-6	10/88	MRPS31,MRPL30, MRPL35, MRPS30, MRPS17,MRPL20, MRPL58,MRPL39, MRPS23,MRPL40
1268844	Mitochondrial translation elongation	1.885E-8	2.493E-6	1.707E-5	9.970E-6	10/89	MRPS31,MRPL30, MRPL35,MRPS30, MRPS17,MRPL20, MRPL58,MRPL39, MRPS23,MRPL40
1339158	Metallothioneins bind metals	4.145E-8	3.654E-6	2.503E-5	2.193E-5	5/11	MT1E,MT1F,MT1H, MT1M,MT1X
1270246	Collagen biosynthesis and modifying enzymes	3.282E-20	1.007E-17	6.905E-17	1.752E-17	20/70	BMP1,P3H1,PLOD2, COL1A1, COL1A2, COL4A5,COL5A1,COL5A2, COL6A1,COL6A2,COL7A1, COL8A1,COL13A1, COL15A1,COL16A1,SERPINH1,P4HA1,P3H2,PCOLCE2, PCOLCE
1270245	Collagen formation	3.771E-20	1.007E-17	6.905E-17	2.014E-17	22/93	BMP1,P3H1,PLOD2,COL1A1 ,COL1A2, COL4A5,COL5A1, COL5A2,COL6A1,COL6A2,COL7A1, COL8A1,COL13A1, COL15A1,COL16A1,SERPINH1,P4HA1,P3H2,LOX,LOXL1, PCOLCE2,PCOLCE
1270244	Extracellular matrix organization	6.993E-19	1.245E-16	8.537E-16	3.734E-16	33/298	SPARC,ACTN1,ITGA5, BGN,BMP1,MFAP4,P3H1, PLOD2, COL1A1, COL1A2, COL4A5, COL5A1,COL5A2,NID2, COL6A1,COL6A2, COL7A1,COL8A1, COL13A1,COL15A1, OL16A1, LAMA4, LAMA5, LAMB2, SERPINH1, CASK,TGFB3, P4HA1,P3H2, LOX,LOXL1,PCOLCE2, PCOLCE
1270247	Assembly of collagen fibrils and other multimeric structures	3.812E-13	5.089E-11	3.490E-10	2.036E-10	14/60	BMP1,COL1A1,COL1A2 , COL4A5, COL5A1,COL5A2, COL6A1,COL6A2,COL7A1, COL8A1, COL15A1,LOX,LOXL1, PCOLCE
1470926	Collagen chain trimerization	6.018E-12	6.427E-10	4.408E-9	3.214E-9	12/47	COL1A1,COL1A2,COL4A5, COL5A1, COL5A2,COL6A1, COL6A2,COL7A1,COL8A1, COL13A1, COL15A1,COL16A1

Kyoto Encyclopedia of Genes and Genomes (KEGG), MSigDB C2 BIOCARTA(M) and PantorDB (P)

MSigDB C2 BIOCARTA (v6.0)

M5889	Ensemble of genes encoding extracellular matrix and extracellular matrix-associated proteins	3.937E-21	5.905E-19	3.301E-18	5.905E-19	62/1028	IL10,PCSK5,SPARC, IL18, CTSC,GAS6,SVEP1, BGN,IK3IP1,BMP1 , MFAP4, FAM20C,SRPX2,P3H1, CCL2,PLAT,C1QTNF1, PLAU,C1QTNF6,PLOD2, CXCL12,COL1A1, COL1A2,PLXNA3, COL4A5,COL5A1, COL5A2,NID2,COL6A1, COL6A2,SEMA4B, COL7A1, COL8A1,SFRP2, COL15A1,COL16A1, ANGPT1 , PAMR1,MMP23B , LAMA4,NTNG1, LAMA5,LAMB2, CXCL14,ECM1, SERPINH1,TGFB3, TGM2,THBS2, ANGPTL2,FSTL1, IGFBP5,IGFBP7, P4HA1,P3H2,LOX, LOXL1,PCOLCE2, MUC1,CTSO,PCOLCE
M5884	Ensemble of genes encoding core extracellular matrix including ECM glycoproteins, collagens and proteoglycans	4.218E-16	3.164E-14	1.769E-13	6.327E-14	29/275	SPARC,GAS6,SVEP1, BGN,MFAP4, SRPX2,COL1A1, COL1A2,COL4A5, COL5A1,COL5A2, NID2,COL6A1,COL6A2, COL7A1,COL8A1, COL13A1,COL15A1, COL16A1,LAMA4, NTNG1,LAMA5, LAMB2,ECM1,THBS2, IGFBP5,IGFBP7, PCOLCE2,PCOLCE
M3005	Genes encoding collagen proteins	2.532E-12	1.266E-10	7.080E-10	3.799E-10	12/44	COL1A1,COL1A2, COL4A5,COL5A1, COL5A2,COL6A1, COL6A2,COL7A1, COL8A1,COL13A1 ,COL15A1,COL16A1
M5887	Genes encoding structural components of basement membranes	9.690E-9	3.634E-7	2.032E-6	1.453E-6	9/40	COL4A5,NID2, COL6A1,COL6A2, COL15A1,LAMA4, NTNG1,LAMA5, LAMB2

M3008	Genes encoding structural ECM glycoproteins	8.983E-8	2.340E-6	1.308E-5	1.348E-5	16/196	SPARC,GAS6, SVEP1,MFAP4, SRPX2,NID2, LAMA4,NTNG1, LAMA5,LAMB2, ECM1,THBS2, IGFBP5,IGFBP7, PCOLCE2,PCOLCE
			**PantherDB**				
P00034	Integrin signalling pathway	1.514E-10	6.209E-9	2.672E-8	6.209E-9	18/167	ACTN1,ITGA5, COL1A1,COL1A2, COL4A5,COL5A1, COL5A2,COL6A1, COL6A2,COL7A1, COL8A1,COL13A1, COL15A1, COL16A1,SHC1, LAMA4,LAMA5, LAMB2
P00036	Interleukin signaling pathway	6.477E-4	1.328E-2	5.714E-2	2.656E-2	7/92	IL10,IL11RA, L13RA2,IL18, STAT3,AKT1,SHC1
P00008	Axon guidance mediated by Slit/Robo	2.711E-3	2.797E-2	1.203E-1	1.111E-1	3/18	ABL1,CXCL12, NTNG1
P00005	Angiogenesis	2.729E-3	2.797E-2	1.203E-1	1.119E-1	8/150	PDGFRA,PDGFRB, STAT3,AKT1, ANGPT1,SHC1, TGFB1I1,FZD2

Table 4 The enriched GO terms of the up-regulated differentially expressed genes

GO ID	Category	GO Name	P Value	FDR B&H	FDR B&Y	Bonferroni	Gene Count	Gene
GO:0006412	BP	translation	4.335E-13	1.190E-9	1.011E-8	1.190E-9	31/667	MRPS31,RPL34,RPL41,RPL36A,RPLP0, RSL1D1,RPS7,ETF1,MRPL30,RPS24, SLC25A26,MRPL35,MRPS30,MRPS17, CNOT7, SNU13,MRRF,NPM1,MRPL20,C8orf88, SLC25A4, MRPL58,RPL14,NHP2,MRPL39,EEF1B2, RPL26L1,EIF4E,MRPS23,MRPL40,CPEB1

GO:0043043	BP	peptide biosynthetic process	1.320E-12	1.812E-9	1.539E-8	3.624E-9	31/696	MRPS31,RPL34,RPL41,RPL36A,RPLP0, RSL1D1,RPS7,ETF1,MRPL30,RPS24, SLC25A26,MRPL35,MRPS30,MRPS17, CNOT7,SNU13,MRRF,NPM1,MRPL20, C8orf88,SLC25A4,MRPL58,RPL14,NHP2, MRPL39,EEF1B2,RPL26L1,EIF4E,MRPS23, MRPL40,CPEB1
GO:0006518	BP	peptide metabolic process	8.626E-12	7.326E-9	6.223E-8	2.368E-8	33/847	MRPS31,RPL34,RPL41,RPL36A,RPLP0, RSL1D1,RPS7,ETF1,MRPL30,RPS24, SLC25A26,MRPL35,MRPS30,MGST1, MRPS17,GLO1,CNOT7,SNU13,MRRF, NPM1,MRPL20,C8orf88,SLC25A4,MRPL58, RPL14,NHP2,MRPL39,EEF1B2,RPL26L1, EIF4E,MRPS23,MRPL40,CPEB1
GO:0042273	BP	ribosomal large subunit biogenesis	1.068E-11	7.326E-9	6.223E-8	2.930E-8	11/59	RSL1D1,RPF2,SNU13,NPM1,MRPL20, NOP16,RRP15,RPL14,NHP2,RPL26L1, WDR12
GO:0043604	BP	amide biosynthetic process	2.453E-11	1.347E-8	1.144E-7	6.734E-8	31/780	MRPS31,RPL34,RPL41,RPL36A,RPLP0, RSL1D1,RPS7,ETF1,MRPL30,RPS24, SLC25A26,MRPL35,MRPS30,MRPS17 ,CNOT7,SNU13,MRRF,NPM1,MRPL20, C8orf88,SLC25A4,MRPL58,NHP2,MRPL39, EEF1B2,RPL26L1,EIF4E,MRPS23,MRPL40, CPEB1
GO:0005840	CC	ribosome	7.260E-16	2.737E-13	1.782E-12	2.737E-13	22/235	MRPS31,RPL34,RPL41,RPL36A,RPLP0, RSL1D1,RPS7,MRPL30,RPS24,MRPL35, MRPS30,MRPS17,SNU13,NPM1,MRPL20, MRPL58,RPL14,NHP2,MRPL39,RPL26L1, MRPS23,MRPL40
GO:0044391	CC	ribosomal subunit	6.895E-14	1.300E-11	8.462E-11	2.599E-11	18/175	MRPS31,RPL34,RPL41,RPL36A,RPLP0, RSL1D1,RPS7,PS24,MRPS17,SNU13, NPM1,MRPL20,MRPL58,RPL14,NHP2, MRPL39,RPL26L1,MRPS23
GO:0015934	CC	large ribosomal subunit	2.160E-11	2.714E-9	1.767E-8	8.142E-9	13/105	RPL34,RPL41,RPL36A,RPLP0,RSL1D1, SNU13,NPM1,MRPL20,MRPL58,RPL14, NHP2,MRPL39,RPL26L1
GO:0030529	CC	intracellular ribonucleoprotein complex	4.722E-10	3.561E-8	2.318E-7	1.780E-7	28/745	MRPS31,RPL34,RPL41,RPL36A,RPLP0, RSL1D1,RPS7,MRPL30,RPS24,MRPL35, MRPS30,MRPS17,CNOT7,EMG1,SNU13, NPM1,MRPL20,MRPL58,RRP15,RPL14 , NHP2,MRPL39,RPL26L1,EIF4E,MRPS23, WDR12,MRPL40,CPEB1
GO:1990904	CC	ribonucleoprotein complex	4.722E-10	3.561E-8	2.318E-7	1.780E-7	28/745	MRPS31,RPL34,RPL41,RPL36A,RPLP0, RSL1D1,RPS7,MRPL30,RPS24,MRPL35, MRPS30,MRPS17,CNOT7,EMG1,SNU13, NPM1,MRPL20,MRPL58,RRP15,RPL14, NHP2,MRPL39,RPL26L1,EIF4E,MRPS23, WDR12,MRPL40,CPEB1

GO:0003735	MF	structural constituent of ribosome	5.292E-11	3.339E-8	2.346E-7	3.339E-8	17/216	MRPS31,RPL34,RPL41,RPL36A,RPLP0,RPS7 ,RPS24,SLC25A26,MRPL35,MRPS30, MRPS17,MRPL20,SLC25A4,RPL14, MRPL39, RPL26L1,MRPS23
GO:0003723	MF	RNA binding	1.472E-6	4.644E-4	3.263E-3	9.289E-4	37/1632	MRPS31,RPL34,RPL41,RPL36A,RPLP0, RSL1D1,RPS7,ETF1,RPS24,MRPS30, MRPS17,CNOT7,RPF2,EMG1,SNU13,STIP1, RBM18,HSPD1,NPM1,MRPL20,NOP16, TCP1,GLRX3,EXOSC3,MRPL58,PPHLN1, RPL14,CMSS1,NHP2,MRPL39,EEF1B2,EIF4E, MRPS23,CSTB,MRPL40,TAF15,CPEB1
GO:0005198	MF	structural molecule activity	7.007E-6	1.474E-3	1.035E-2	4.421E-3	22/762	MRPS31,RPL34,RPL41,RPL36A,RPLP0,RPS7, TUBA3E,RPS24,SLC25A26,MRPL35, MRPS30,MRPS17,HOMER2,MRPL20, SLC25A4,TFPI2,RPL14,AKAP12,MRPL39, NDC1, RPL26L1, MRPS23
GO:0019843	MF	rRNA binding	5.289E-5	8.344E-3	5.862E-2	3.338E-2	6/67	RPLP0,MRPS17,RPF2,EMG1,NPM1, MRPL20

Biological Process(BP), Cellular Component(CC) and Molecular Functions (MF)

Table 5 The enriched GO terms of the down-regulated differentially expressed genes

GO ID	Category	GO Name	P Value	FDR B&H	FDR B&Y	Bonferroni	Gene Count	Gene
GO:0030198	BP	extracellular matrix organization	1.799E-21	4.372E-18	3.919E-17	7.910E-18	38/354	SPARC,ABL1,PDGFRA,GAS6,ITGA5, BGN,BMP1,MFAP4,P3H1,RECK, COL1A1,COL1A2,COL4A5,COL5A1, COL5A2,DPP4,NID2,COL6A1, COL6A2,COL7A1,COL8A1,SFRP2, COL13A1,COL16A1,ERO1A,LAMA4, LAMA5,LAMB2,APLP1,LCP1, SERPINH1,GSN,P4HA1,LOX,LOXL1, FSCN1,ENG,PHLDB2
GO:0043062	BP	extracellular structure organization	1.988E-21	4.372E-18	3.919E-17	8.743E-18	38/355	SPARC.ABL1,PDGFRA,GAS6,ITGA5, BGN,BMP1,MFAP4,P3H1,RECK, COL1A1,COL1A2,COL4A5,COL5A1, COL5A2,DPP4,NID2,COL6A1, COL6A2,COL7A1,COL8A1,SFRP2, COL13A1,COL16A1,ERO1A,LAMA4, LAMA5,LAMB2,APLP1,LCP1, SERPINH1,GSN,P4HA1,LOX,LOXL1, FSCN1,ENG,PHLDB2
GO:0032963	BP	collagen metabolic process	2.318E-15	3.397E-12	3.046E-11	1.019E-11	20/122	IL18,PDGFRB,MFAP4,P3H1,CCL2, COL1A1,COL1A2,COL4A5,COL5A1, COL5A2,COL6A1,COL6A2,COL7A1, COL8A1,COL13A1,COL15A1, SERPINH1,TGFB3,P3H2,ENG

GO:0044259	BP	multicellular organismal macromolecule metabolic process	5.179E-15	5.693E-12	5.104E-11	2.277E-11	20/127	IL18,PDGFRB,MFAP4,P3H1,CCL2, COL1A1,COL1A2,COL4A5,COL5A1, COL5A2,COL6A1,COL6A2,COL7A1, COL8A1,COL13A1,COL15A1, SERPINH1,TGFB3,P3H2,ENG
GO:0022610	BP	biological adhesion	6.524E-15	5.737E-12	5.144E-11	2.868E-11	66/1542	TPM1,IL10,ERBB2,IL18,ABL1, PDGFRA,CADM4,HLA-DRB1,HLA-DRB4,PCDHB5,GAS6,GBP2,TNIP1, ACTN1,ITGA5,SVEP1,MFAP4,FAT1, SRPX2,PTPRF,NUAK1,CLPTM1, CCL2,AKT1,C1QTNF1,PLAU, FERMT2,CXCL12,COL1A1,COL5A1, DPP4,NID2,COL6A1,COL6A2, COL7A1,COL8A1,SFRP2,COL13A1 ,COL15A1,COL16A1,ANGPT1,SHC1, LAMA4,AIRE,LAMA5,LAMB2, APLP1,LCP1,DICER1,NLRP3,GSN ,CASK,TGFB1I1,PDPN,TGM2,SSPN, CD2,THBS2,CELSR3,IGFBP7,FEZ1, CERCAM,ENG,MUC1,MARCKS, PHLDB2
GO:0031012	CC	extracellular matrix	2.772E-18	1.231E-15	8.215E-15	1.231E-15	38/444	SPARC,BGN,BMP1,MFAP4,P3H1, MAMDC2,PLAT,COL1A1,COL1A2, COL4A5,COL5A1,COL5A2,NID2, COL6A1,COL6A2,COL7A1,COL8A1, SFRP2,COL15A1,COL16A1, ANG,LMCD1,MMP23B,LAMA4, LAMA5,LAMB2,APLP1,ECM1, CASK,TGFB1I1,TGFB3,TGM2, THBS2,IGFBP7,P3H2,LOX,LOXL1, PCOLCE
GO:0044420	CC	extracellular matrix component	1.907E-16	4.234E-14	2.826E-13	8.468E-14	22/141	SPARC,MFAP4,P3H1,COL1A1, COL1A2,COL4A5,COL5A1,COL5A2, NID2,COL6A1,COL7A1,COL8A1, COL15A1,ANG,LAMA4,LAMA5, LAMB2,APLP1,CASK,THBS2,P3H2, LOXL1
GO:0005578	CC	proteinaceous extracellular matrix	2.287E-15	3.385E-13	2.259E-12	1.016E-12	32/379	SPARC,BGN,BMP1,MFAP4,P3H1, MAMDC2,COL1A1,COL1A2, COL4A5,COL5A1,COL5A2,NID2, COL6A1,COL6A2,COL7A1,COL8A1, COL15A1,COL16A1,ANG,MMP23B, LAMA4,LAMA5,LAMB2,APLP1, ECM1,CASK,TGFB3,TGM2,THBS2, P3H2,LOX,LOXL1
GO:0005604	CC	basement membrane	5.503E-13	6.108E-11	4.076E-10	2.443E-10	17/109	SPARC,P3H1,COL4A5,COL5A1, NID2,COL7A1,COL8A1,COL15A1 ,ANG,LAMA4,LAMA5,LAMB2, APLP1,CASK,THBS2,P3H2,LOXL
GO:0005615	CC	extracellular space	1.078E-12	9.573E-11	6.389E-10	4.786E-10	59/1449	IL10,PCSK5,IL13RA2,SPARC,IL18, PDE4C,MAN2A1,CTSC,GAS6,NBL1, ACTN1,CHI3L2,CKB,ADM,BMP1, FAM20C,SRPX2,SCG2,CCL2,PLAT, C1QTNF1,PLAU,C1QTNF6,HSPA2,

								CXCL12,PCYOX1,COL1A1,COL1A2, COL6A2,VLDLR,SEMA4B,COL7A1, SFRP2,COL15A1,ANG,ANGPT1, LMCD1,PPFIBP2,VASH1,LAMA5, CXCL14,CPE,LCP1,ECM1, SERPINH1,GSN,TGFB3,ANGPTL2, FSTL1,IGFBP5,IGFBP7,LOX,LOXL1, PAM,ENG,MUC1,CTSO,LRRC17, PCOLCE
GO:0048407	MF	platelet-derived growth factor binding	1.009E-8	9.008E-6	6.641E-5	9.008E-6	6/12	PDGFRA,PDGFRB,COL1A1,COL1A2, COL5A1,COL6A1
GO:0019838	MF	growth factor binding	3.652E-8	1.630E-5	1.202E-4	3.261E-5	14/142	IL11RA,ERBB2,PDGFRA,PDGFRB, SRPX2,COL1A1,COL1A2, COL5A1 , COL6A1,SHC1,TGFB3,IGFBP5, IGFBP7,ENG
GO:0005539	MF	glycosaminoglycan binding	7.023E-6	1.999E-3	1.474E-2	6.272E-3	14/219	BGN,PTPRF,MAMDC2,CCL2, COL5A1,COL13A1,ANG,APLP1, NLRP3,THBS2,FSTL1,PCOLCE2, ENG,PCOLCE
GO:0005102	MF	receptor binding	9.287E-6	1.999E-3	1.474E-2	8.293E-3	47/1601	IL10,ERBB2,IL18,ABL1,HLA-B,PDGFRA,PDGFRB,CADM4,GAS6, ACTN1,ITGA5,STAT3,ADM,BMP1, SRPX2,PTPRF,SCG2,CCL2,PLAT, FAM3C,CXCL12,COL5A1,DPP4, SEMA4B,SFRP2,COL16A1,ANG, ANGPT1,PILRB,SHC1,LAMA4, LAMA5,LAMB2,CXCL14,APLP1, CPE,CAV2,IDUA,ECM1,CASK, TGFB1I1,TGFB3,NCOR2,CD2, ANGPTL2,SMARCD3,ENG
GO:0031418	MF	L-ascorbic acid binding	1.313E-5	1.999E-3	1.474E-2	1.173E-2	5/21	P3H1,PLOD2,P4HA1,P3H2,PAM

Biological Process(BP), Cellular Component(CC) and Molecular Functions (MF)

Table 6 Topology table for up and down regulated genes

Regulation	Node	Degree	Betweenness	Stress	Closeness	Clustering Coefficient
UP	NPM1	945	0.301626	1.52E+08	0.393093	0.004613
UP	CUL4A	650	0.109005	41895530	0.35694	0.003079
UP	PSME3	545	0.105846	39532730	0.342083	9.64E-04
UP	GLRX3	491	0.039865	33998508	0.293765	0
UP	HSPD1	430	0.079507	50527810	0.339525	0.002241
UP	EIF4E	392	0.033837	20526756	0.31111	0.038073
UP	RPL14	362	0.041203	19606474	0.34047	0.039971
UP	GEM	348	0.035319	17519240	0.272334	0

UP	RPL26L1	326	0.027836	10748090	0.327193	0.046006
UP	TCP1	289	0.058986	24645646	0.332327	0.002048
UP	RPLP0	282	0.022584	18458904	0.337109	0.053245
UP	MAPKAP1	263	0.032676	20062178	0.277936	0
UP	RPS7	258	0.022248	21031692	0.325236	0.05825
UP	ELMO1	255	0.052834	24104106	0.269749	0
UP	MRPL58	252	0.073303	14201496	0.329543	0.010795
UP	MAGEA1	212	0.024457	14631838	0.275985	0
UP	HEBP1	210	0.08713	41181458	0.27306	0
UP	STIP1	210	0.042784	39773200	0.302853	0
UP	RPS24	209	0.0116	8465680	0.330284	0.0803
UP	CCND3	209	0.01113	5731318	0.274832	0
UP	CNOT7	207	0.018381	8484440	0.28965	0.02057
UP	RPL34	199	0.005788	5349978	0.311263	0.08545
UP	NDUFA9	195	0.036534	17629808	0.305563	0.014325
UP	EEF1B2	186	0.01062	7007208	0.329494	0.095766
UP	RPL36A	179	0.004607	3912490	0.308193	0.093292
UP	ETF1	177	0.013593	9182680	0.307569	0.084481
UP	RAC2	170	0.06204	44831196	0.287176	0
UP	RPL41	169	0.008327	3801038	0.309644	0.101183
UP	COX5B	154	0.019084	17307864	0.26744	0
UP	SNU13	153	0.031235	35847562	0.275037	0
UP	PCBD1	151	0.015214	2920542	0.30457	0.009428
UP	NDUFB9	146	0.013293	7259370	0.271244	0.015454
UP	MRPS30	136	0.009211	1995560	0.310015	0.074234
UP	NTS	134	0.048506	24963062	0.250997	0
UP	HMGA1	129	0.021558	28910786	0.285372	0
UP	EXOSC3	126	0.011782	3484842	0.281362	0.035714
UP	MRPL39	124	0.009498	1822486	0.312301	0.072379
UP	RAD51C	122	0.010761	10526584	0.245911	0
UP	POLR1D	112	0.02595	19110742	0.264057	0
UP	ALDH3B1	108	0.011533	6346056	0.272014	0
UP	AP1S1	99	0.024439	10811160	0.269386	0
UP	MRPS31	99	0.009701	4657428	0.284359	0.020632
UP	TTC1	96	0.014675	8619812	0.282263	0
UP	LDHB	92	0.00874	10241224	0.285594	0
UP	CENPM	91	0.027478	48791388	0.263221	0
UP	MRPL40	84	0.00325	1663794	0.279292	0.093651
UP	POMP	76	0.005661	3384902	0.257211	0.025397
UP	MTERF3	75	0.00553	1941298	0.269353	0.028226
UP	ATP6V0D2	75	0.019339	12095016	0.260358	0
UP	AMPH	73	0.013216	3958518	0.259011	0
UP	CD33	72	0.004162	1416990	0.243707	0

UP	ACOT7	71	0.005973	1921782	0.245049	0
UP	TSPAN17	70	0.016109	4269350	0.233489	0
UP	DKK1	70	0.005498	2024836	0.232675	0
UP	ORC5	70	0.012508	15541348	0.263552	0
UP	IL36RN	69	0.005426	1851816	0.236713	0
UP	PPHLN1	69	0.013153	8136810	0.272654	0
UP	SLC25A4	67	0.014795	11773820	0.283827	0.00508
UP	GALNT12	67	0.018289	7714008	0.256388	0
UP	RPF2	66	0.007214	1621040	0.31462	0.02935
UP	MRPS23	65	0.006532	4806344	0.286933	0.026361
UP	GRPEL1	63	0.013371	4830022	0.281452	0.00443
UP	ACAT2	62	0.003268	2163656	0.267911	0
UP	EVA1C	61	0.014548	3594904	0.231246	0
UP	EMG1	60	0.005908	1328458	0.307419	0.018519
UP	MRPL20	60	0.004654	2128160	0.274164	0.095798
UP	NDUFAF4	59	0.004597	3012546	0.259194	0
UP	RRAGD	59	0.003646	1230298	0.224207	0
UP	PSMG1	58	0.004777	2613824	0.262062	0.045584
UP	TAF15	56	0.009834	3508062	0.297575	0.009752
UP	WDR12	55	0.003156	2610562	0.248547	0
UP	SFR1	55	0.002244	1028382	0.241807	0
UP	NMT2	53	0.011852	9935166	0.264517	0
UP	BCKDK	52	0.008128	2811708	0.259606	0
UP	MRPL35	50	0.002989	859940	0.272755	0.099884
UP	MNS1	50	0.004934	1907394	0.257557	0
UP	PLA2G16	49	0.009765	3370618	0.236447	0
UP	TOMM34	49	0.005237	6263964	0.261393	0
UP	RSL1D1	48	0.003372	1797580	0.312456	0.031039
UP	BATF3	48	0.010848	3593886	0.262202	0
UP	RECQL	46	0.005662	4115656	0.265234	0
UP	NHP2	46	0.006025	3726180	0.269964	0
UP	LYST	45	0.006491	2117548	0.25414	0.002646
UP	GNPTAB	45	0.007501	2949966	0.229248	0
UP	GNG11	45	0.004851	2734780	0.247166	0
UP	TTC26	43	0.0091	3134914	0.249208	0
UP	AKAP12	43	0.006325	4665754	0.262671	0
UP	GCNT3	40	0.009466	3111594	0.247583	0
UP	NDC1	40	0.011972	8968156	0.265282	0
UP	TPRKB	39	0.002992	975934	0.255674	0
UP	CMTM7	38	0.006755	3353658	0.18482	0
UP	MRRF	36	0.00663	1642102	0.264962	0.003953
UP	C12orf49	36	0.004996	3932028	0.220437	0
UP	AIM2	34	0.003617	1480124	0.224562	0

UP	PROCR	34	0.0069	1428924	0.212228	0
UP	PWP1	33	0.004575	819506	0.305055	0.054187
UP	C11orf1	33	0.004189	2788740	0.214178	0
UP	TUBA3E	33	0.00261	1908738	0.255126	0
UP	CSTB	33	0.001309	1181778	0.270063	0
UP	MRPL30	32	5.64E-04	379396	0.254831	0.150198
UP	RPE	31	0.001824	571484	0.236802	0
UP	SLC25A46	31	0.006306	1067264	0.220714	0
UP	HOMER2	30	0.003451	1032842	0.243923	0
UP	CTSK	30	0.007625	2047938	0.247111	0
UP	BOLA3	29	0.006744	1134428	0.268827	0
UP	CMSS1	29	0.006321	1205830	0.287814	0.003953
UP	AGPAT5	29	0.007633	2487268	0.222362	0
UP	SNX8	28	0.006214	3222756	0.237812	0
UP	TIMM10	28	0.003625	1353168	0.234022	0
UP	CDCA2	28	0.003243	2449836	0.259851	0
UP	UNG	28	0.002921	4060574	0.254742	0
UP	HRK	27	0.00105	299978	0.23693	0
UP	YIPF1	25	1	42	1	0
UP	MND1	25	0.003582	3195092	0.254757	0
UP	ARC	23	0.005004	6401076	0.183282	0
UP	RRP15	22	0.001183	1079650	0.268434	0
UP	NEMP1	21	0.008595	3526912	0.241264	0
UP	OXCT1	21	0.002983	1300180	0.253876	0
UP	CCL26	21	0.002294	826426	0.239545	0
UP	NOP16	20	0.002057	309258	0.292964	0.023392
UP	RBM18	20	0.003647	635716	0.228974	0
UP	COQ3	20	0.003469	1066612	0.242339	0
UP	SBSN	20	0.005063	2350816	0.252175	0
UP	ALPL	20	6.86E-04	743310	0.247444	0
UP	FAM92A	19	0.001118	470888	0.217724	0
UP	DHRS9	19	0.003222	1834928	0.190866	0
UP	C12orf45	18	0.002549	847750	0.238611	0
UP	ME2	17	0.002159	808064	0.26904	0
UP	LBHD1	16	0.002392	1811616	0.242259	0
UP	MT1X	16	0.001364	1528356	0.211352	0
UP	TMEM126A	16	0.004142	1990502	0.237774	0
UP	DTNA	15	0.001428	417488	0.215046	0
UP	MAGEC1	15	0.002301	561898	0.236586	0
UP	KYNU	14	0.003176	1178128	0.162931	0
UP	THY1	14	0.002354	524132	0.220736	0
UP	MRPS17	13	0.001302	564214	0.260635	0
UP	SMAGP	13	0.001347	1091748	0.178398	0

UP	TM4SF19	12	0.001455	545230	0.217081	0
UP	CDH11	12	0.00265	857172	0.23807	0
UP	GAGE1	12	6.49E-05	43402	0.211718	0
UP	MT1M	12	0.00122	531098	0.244017	0
UP	PIGP	12	0.002292	902270	0.160298	0
UP	TIGAR	12	0.001374	440528	0.234983	0
UP	M6PR	12	0.003284	902224	0.238057	0
UP	MGST1	11	0.001541	485838	0.248197	0
UP	SPTLC3	11	1	6	1	0
UP	CFAP36	11	0.003201	1703976	0.179247	0
UP	GLO1	11	0.001682	1051088	0.251341	0
UP	TDP1	11	0.001584	478454	0.23172	0
UP	EFCAB7	10	1	30	1	0
UP	MT1H	10	4.54E-04	95670	0.158163	0
UP	VGF	9	4.95E-04	261878	0.254624	0
UP	TMEM38B	9	0.001458	892914	0.207979	0
UP	GTF3C6	9	0.001416	466460	0.218069	0
UP	MCFD2	9	0.002269	1096058	0.167262	0
UP	SPR	8	9.59E-04	366092	0.233786	0
UP	GAGE2B	8	6.05E-04	786762	0.187128	0
UP	THYN1	8	0.001261	522284	0.256298	0
UP	GAGE6	6	1.51E-04	393376	0.187112	0
UP	PTGES	5	0	0	0.192048	0
UP	ANKRD39	5	8.62E-05	76166	0.250014	0
UP	MT1F	5	0	0	0.223343	0
UP	TATDN1	5	4.54E-04	202112	0.195595	0
UP	CPEB1	4	1	2	1	0
UP	TFPI2	4	7.45E-05	30926	0.208669	0
UP	MT1E	3	3.88E-05	18910	0.235285	0
UP	TMPRSS15	3	0	0	0.155467	0
UP	ZNF586	2	1	2	1	0
UP	SPINK1	2	0	0	1	0
UP	IL36B	1	0	0	0.217038	0
UP	PKD1L2	1	0	0	0.176638	0
UP	ZDHHC12	1	0	0	0.125349	0
UP	SP140	1	0	0	1	0
UP	LYPLAL1	1	0	0	0.186748	0
UP	TAGLN3	1	0	0	1	0
Down	SHC1	1092	0.123965	58212628	0.368086	0.004075
Down	AKT1	884	0.119658	1.07E+08	0.356054	0.001681
Down	STAT3	693	0.093758	71075836	0.344698	0.001575
Down	ABL1	644	0.103055	72823840	0.348882	0.001547
Down	ERBB2	623	0.056468	31545102	0.345783	0.004291

Down	LDOC1	434	0.025486	6721166	0.298889	0.001237
Down	TNIP1	422	0.030998	22969838	0.307133	0
Down	ACTN1	370	0.030143	20962894	0.323836	0.001567
Down	NCOR2	319	0.047137	37159610	0.314902	0.003954
Down	COL8A1	308	0.012797	7316652	0.304865	0.028891
Down	HNRNPA2B1	304	0.058735	34643382	0.338722	4.61E-04
Down	PDGFRB	276	0.021343	13762004	0.330404	0.01563
Down	SPRY1	264	0.020628	15205000	0.306105	0.006683
Down	PDLIM7	263	0.039346	17926678	0.324641	0.004214
Down	PCSK5	248	0.008888	5312074	0.282349	0.013221
Down	MUC1	232	0.023834	8834952	0.31681	0.008025
Down	ULK1	231	0.014321	4663448	0.296005	0.001586
Down	TPM1	228	0.019333	7172214	0.304831	0.016147
Down	CXCL12	227	0.034488	56002026	0.289237	0.009121
Down	SSTR2	219	0.033341	57301776	0.286146	0.009476
Down	DICER1	204	0.019009	15408606	0.291525	0
Down	RHOJ	200	0.023077	16574106	0.289359	0.008249
Down	HOXB5	200	0.022713	13386288	0.303903	4.58E-04
Down	DAPP1	185	0.005064	2290332	0.298532	0.017826
Down	HOXC8	184	0.023429	8548472	0.31133	0.002554
Down	COL1A2	179	0.021259	17218962	0.322316	0.05931
Down	TGM2	177	0.018612	8827328	0.315862	0.002404
Down	APLP1	177	0.0099	4492668	0.291432	0
Down	EPM2AIP1	173	0.009991	2304004	0.287826	0.003846
Down	COL1A1	167	0.023895	18695344	0.324391	0.053318
Down	AIRE	154	0.022399	12156104	0.315789	0.002912
Down	NLRP3	153	0.015111	6377934	0.288325	0.001695
Down	TPM2	152	0.025473	7972520	0.311348	0.01329
Down	CASK	151	0.008606	4709624	0.269316	0
Down	FEZ1	144	0.016182	3975414	0.295161	9.92E-04
Down	ERO1A	144	0.034597	32740382	0.283589	1.50E-04
Down	PDGFRA	138	0.011853	5589982	0.316719	0.024144
Down	HSPA2	137	0.021505	25355280	0.301997	0
Down	ECM1	133	0.011579	7163332	0.285076	0.001169
Down	IL13RA2	133	0.030231	7572740	0.303818	0.001078
Down	ENG	131	0.002987	1313786	0.273461	0.038961
Down	AKAP13	130	0.017283	8134770	0.298046	0.013812
Down	PHLDB2	126	0.011433	6453964	0.292862	4.02E-04
Down	GSN	126	0.015162	18679874	0.300345	0
Down	SMARCD3	122	0.010317	5582800	0.288325	0.015959
Down	HSPB6	122	0.010944	9132014	0.261429	0
Down	ZFYVE21	120	0.003171	3405556	0.266634	0
Down	CKB	117	0.007648	3119446	0.302897	0.003322

Down	NAV1	117	0.006075	4857636	0.27599	0
Down	RECK	116	0.004907	2120414	0.28942	0
Down	QPRT	116	0.011036	13444886	0.248012	0
Down	ABLIM1	115	0.0148	10900168	0.312358	0.004566
Down	LAMA4	108	0.005756	1808214	0.28942	0.039112
Down	FRMD6	108	0.004551	2978894	0.274075	0
Down	SERPINH1	104	0.015932	8954600	0.324219	0.067413
Down	THRA	104	0.009514	4747398	0.288053	0.013575
Down	CBS	103	0.004899	3454832	0.287902	0
Down	CERCAM	100	0.008339	7344280	0.266505	0
Down	COL6A2	99	0.010664	4212442	0.29199	0.091549
Down	FHL1	99	0.011976	3127700	0.312091	0.007401
Down	KDM5B	97	0.013764	13655368	0.28232	0
Down	FSCN1	97	0.006824	6952304	0.291742	0
Down	TUBG2	96	0.017449	19517480	0.286954	0
Down	MAGED2	93	0.012383	13641950	0.298094	0
Down	ITGA5	93	0.007525	4747812	0.292706	0.004646
Down	ADM	93	0.023088	40126436	0.247856	0
Down	COL4A5	92	0.005218	2445854	0.295989	0.138431
Down	COL7A1	92	0.003902	1832162	0.293113	0.148077
Down	LOX	92	0.003612	1407304	0.290108	0.036782
Down	WDR26	92	0.014961	9211540	0.303281	0.004464
Down	MCM8	92	0.018293	19422074	0.286012	0
Down	FSTL1	92	0.006647	3628196	0.271863	0
Down	CCL2	91	0.003586	5339100	0.257227	0
Down	P3H1	90	0.012122	5268876	0.311631	0.066387
Down	PRDM1	89	0.013478	14092258	0.285076	5.84E-04
Down	HNRNPDL	89	0.01039	11875414	0.29618	0
Down	BMP1	83	0.01073	3106430	0.292847	0.042199
Down	CLIP4	82	0.005392	1475428	0.274157	0.014778
Down	COL6A1	76	0.005091	2847790	0.297207	0.120328
Down	PLXNA3	75	0.014425	6381048	0.301036	0.00121
Down	FAM19A3	74	0.019547	11527582	0.267414	0
Down	RNF216	74	0.00527	4458584	0.254226	0
Down	LAMB2	71	0.01029	3667434	0.30614	0.046465
Down	COL5A1	70	0.002378	1308374	0.29083	0.151977
Down	PYGL	70	0.007461	7623440	0.26644	0
Down	KLHDC2	69	0.010627	5906618	0.272484	0
Down	NUAK1	68	0.003319	2114704	0.277848	0
Down	IGFBP5	67	0.007589	1812356	0.25982	0
Down	CPE	67	0.002836	1387998	0.244772	0
Down	CD2	67	0.002235	2797184	0.257131	0
Down	LAMA5	64	0.00414	2182024	0.287947	0.065338

Down	GBP2	63	0.006039	9035820	0.265269	0
Down	BGN	62	0.013183	5291372	0.267805	0.016162
Down	DACT1	62	0.00168	1954074	0.271944	0
Down	LDB2	61	0.004815	1799672	0.261031	0.003333
Down	FAM3C	60	0.006118	1420720	0.258633	0.002646
Down	NFIX	60	0.007835	5099496	0.276602	0.00135
Down	C1QTNF1	60	2.40E-04	151982	0.222899	0
Down	LGMN	59	0.008703	2046824	0.285358	0.014103
Down	COL5A2	58	0.001611	1024074	0.292021	0.185884
Down	TGFB3	58	0.003358	2516710	0.254343	0.031579
Down	MAP4K4	57	0.009345	8407794	0.295973	0
Down	PTPRF	57	0.008782	6104046	0.289008	0.00135
Down	SLC26A6	57	0.007205	7390250	0.244489	0
Down	DPP4	56	0.002371	2238784	0.25142	0
Down	GAS6	53	0.00544	1432578	0.26273	0.005698
Down	SGSM2	53	0.004346	1908940	0.267361	0
Down	TGFB1I1	53	0.00253	2752252	0.27051	0
Down	P4HA1	51	0.005333	4169942	0.26962	0
Down	GSTA4	50	0.002244	1504920	0.212626	0
Down	NID2	49	0.003334	1097194	0.268407	0.089076
Down	LUC7L3	49	0.00593	3586268	0.297174	0.001587
Down	IL10	48	0.003597	861210	0.285432	0.021053
Down	TNS3	48	0.002419	2123010	0.279205	0
Down	FZD2	47	0.009644	3202854	0.283604	0
Down	ANG	47	0.001572	628628	0.265	0
Down	EVI5L	47	0.0065	3735200	0.256016	0
Down	COL16A1	46	9.09E-04	533796	0.287751	0.233333
Down	SFRP2	46	0.001631	1653632	0.261678	0
Down	PDE4C	45	0.002801	4729838	0.25695	0
Down	TAGLN	45	0.002292	1928898	0.254284	0
Down	SIPA1L2	42	0.004583	1279310	0.278017	0
Down	LRP10	40	0.005853	4794446	0.231051	0
Down	RSRP1	39	0.004155	2370844	0.23323	0
Down	CELSR3	38	0.008017	1695216	0.292613	0.006048
Down	MYLK	38	0.002117	1217032	0.287164	0.095238
Down	ASAP3	38	0.002598	1241474	0.253779	0
Down	FAT1	37	0.005624	1803368	0.271272	0
Down	SPARC	37	0.00208	687530	0.264885	0.008333
Down	PLEKHA1	37	0.003085	2616724	0.251086	0
Down	SCG2	37	2.87E-04	270752	0.258791	0
Down	DDIT4	37	7.65E-04	537070	0.274363	0
Down	IL18	36	0.00231	1111182	0.242695	0
Down	PLAT	36	0.004839	3100588	0.239414	0

Down	SLC15A3	36	7.40E-04	625402	0.248675	0
Down	PPIC	36	0.001538	1373418	0.216431	0
Down	H1F0	35	0.005249	4747144	0.275174	0
Down	TTC3	34	0.003038	652586	0.293457	0.026316
Down	RRBP1	34	0.004594	3461946	0.277609	0
Down	ATP6AP1	33	0.00368	928556	0.285165	0.004329
Down	MFAP4	33	0.002012	1010338	0.271998	0.033333
Down	CXCL14	33	4.03E-05	24618	0.220695	0
Down	FERMT2	33	0.003537	2498500	0.277175	0
Down	FAM20C	33	0.001162	769176	0.240652	0
Down	PDLIM3	32	0.001043	274996	0.26219	0.015152
Down	PRR5L	32	1.80E-05	10990	0.235329	0
Down	CDAN1	31	0.0014	491038	0.247867	0
Down	ANGPT1	30	5.90E-04	282310	0.292379	0.091667
Down	CCDC102A	30	0.004588	1560406	0.25998	0.007246
Down	CAV2	30	0.00204	2123302	0.252124	0
Down	GALNT10	29	0.005365	1654292	0.243848	0.02381
Down	KLF11	29	7.07E-04	589510	0.275976	0
Down	PAM	29	0.002612	1232062	0.231246	0
Down	PLAU	28	0.001537	761178	0.254449	0
Down	PAPSS2	28	0.005223	2107194	0.2644	0
Down	LCP1	28	0.002174	1939890	0.264171	0
Down	PTGFRN	27	0.001957	1164272	0.247465	0
Down	PCOLCE2	26	0.001529	644414	0.257408	0.162055
Down	IFITM3	26	5.92E-04	248608	0.28567	0.111111
Down	PIK3IP1	26	5.08E-05	46004	0.240747	0
Down	RARRES3	26	6.98E-04	716306	0.257565	0
Down	PLOD2	25	9.94E-04	913538	0.253042	0
Down	EPSTI1	24	0.002777	763310	0.292878	0.013072
Down	PSRC1	24	0.003038	1858634	0.263019	0
Down	BST2	23	0.00159	1087236	0.257179	0
Down	ZHX2	23	0.001679	1110158	0.263524	0
Down	NREP	23	4.06E-04	366878	0.214127	0
Down	PDPN	23	4.92E-04	342726	0.243751	0
Down	ZHX3	23	9.55E-04	585224	0.23955	0
Down	P3H2	22	0.001415	515958	0.26426	0.022059
Down	LOXL1	22	8.06E-04	327914	0.260795	0.141026
Down	PPFIBP2	22	6.08E-04	511546	0.252159	0
Down	VLDLR	22	1	2	1	0
Down	VASH1	21	0.001943	1214222	0.240885	0
Down	IDUA	21	0.001636	1161236	0.235037	0
Down	PCYOX1	21	0.002968	1461502	0.228683	0
Down	MARCKS	21	0.0012	1431898	0.268224	0

Down	CLIP3	20	0.001499	528338	0.268801	0.044444
Down	HSD17B6	20	0.003023	1003338	0.230769	0
Down	SIDT2	20	0.002336	1002570	0.208674	0
Down	RYR1	19	0.002069	2175076	0.253802	0
Down	CTSC	19	0.001913	1422046	0.240885	0
Down	JMJD8	19	0.002472	1860564	0.235047	0
Down	SPTSSA	19	0.002218	1000408	0.228474	0
Down	FUCA1	19	0.002386	1436696	0.2402	0
Down	LMCD1	19	0.001215	970668	0.248225	0
Down	CHPF2	18	0.002939	1470318	0.241989	0.036765
Down	ASIC1	18	0.001882	908402	0.232657	0
Down	FAM126B	18	5.81E-04	562938	0.273012	0
Down	C1QTNF6	18	7.82E-04	513412	0.2196	0
Down	FAM46C	18	2.32E-04	158076	0.251662	0
Down	CCNG2	18	0.001916	1490586	0.227028	0
Down	RBM33	18	0.002477	1916460	0.262428	0
Down	THBS2	18	0.002975	3867136	0.230983	0
Down	CLPTM1	18	0.00299	1240010	0.264476	0
Down	PCOLCE	17	8.53E-05	45856	0.259244	0.375
Down	AKR1D1	17	0.002595	1297132	0.227263	0
Down	TMEM2	17	0.001912	1587340	0.278765	0
Down	C6orf120	17	0.002965	2114430	0.253545	0
Down	PRAG1	17	4.85E-04	633908	0.252136	0
Down	D2HGDH	15	0.002984	1293952	0.230401	0
Down	CNN2	15	0.001594	1445236	0.259746	0
Down	COL13A1	14	1.05E-04	65232	0.269858	0.166667
Down	PCDHB5	14	0.001793	973546	0.255682	0
Down	FARP1	14	0.002763	893434	0.271191	0.021978
Down	TCEAL2	14	0.002114	635898	0.283633	0
Down	CNIH1	14	0.001182	383438	0.220934	0
Down	MXD4	14	3.64E-04	108850	0.202658	0
Down	IGFBP7	14	7.70E-04	484138	0.236995	0
Down	COL15A1	13	2.03E-05	8484	0.250777	0.333333
Down	CMIP	13	0.001155	692868	0.258207	0
Down	PNISR	13	0.001333	832530	0.262906	0
Down	SVEP1	13	4.78E-04	395906	0.231988	0
Down	HOXC6	12	4.82E-04	262132	0.237047	0
Down	DACT3	12	1.39E-06	3290	0.197617	0
Down	GPT2	11	8.86E-04	537224	0.253838	0
Down	SLC25A42	11	5.89E-05	61924	0.229017	0
Down	TMEM108	11	4.74E-04	231172	0.220393	0
Down	MAN2A1	11	0.001152	746898	0.214831	0
Down	IGSF3	10	0.001	702820	0.26398	0

Down	DEXI	10	7.29E-04	297578	0.198855	0
Down	IFITM2	10	7.58E-04	584330	0.254414	0
Down	MAMDC2	9	0.001131	253708	0.222547	0
Down	GPX8	9	0.001148	222058	0.241531	0.035714
Down	IL11RA	9	3.69E-04	183838	0.23882	0
Down	KIFC2	8	4.28E-04	103042	0.268683	0.133333
Down	ANGPTL2	8	3.74E-04	225168	0.234555	0
Down	ZNF541	8	3.64E-04	110998	0.21473	0
Down	ABCC3	8	0.002189	1138928	0.208976	0
Down	TM9SF1	8	0.001274	718152	0.230517	0
Down	FIBCD1	7	0	0	1	0
Down	CTSO	7	7.45E-04	382130	0.2045	0
Down	PYROXD2	7	2.16E-05	12400	0.21803	0
Down	TGIF2	7	6.54E-05	51664	0.243697	0
Down	ADSSL1	7	0.001193	748042	0.236924	0
Down	PILRB	7	4.60E-04	187910	0.240547	0
Down	NPEPL1	7	6.85E-06	2370	0.201291	0
Down	HOXD10	7	3.84E-05	20346	0.21644	0
Down	DENND5A	6	1.16E-05	6786	0.226289	0
Down	ZNF358	6	3.90E-04	139154	0.20612	0
Down	BHMT2	6	7.29E-04	262614	0.218013	0
Down	SLC25A37	6	0.001095	497474	0.210716	0
Down	IGIP	6	0	0	0.209231	0
Down	SLC44A2	6	0.00182	1340556	0.204218	0
Down	C2orf69	6	7.99E-04	383358	0.229496	0
Down	CADM4	6	7.41E-04	305676	0.208318	0
Down	SRPX2	6	3.75E-04	97888	0.205241	0
Down	NTNG1	5	5.26E-05	13420	0.224237	0
Down	CYP2J2	5	7.38E-04	336912	0.195013	0
Down	HSD17B2	5	0.001093	457452	0.238841	0
Down	C21orf58	5	3.93E-04	250496	0.242609	0
Down	DAP	5	3.90E-07	146	0.212948	0
Down	SLC44A4	4	2.00E-07	12	0.145035	0
Down	MMP23B	4	3.64E-04	182042	0.229065	0
Down	TMEM45A	4	0	0	0.201831	0
Down	MYL5	4	3.35E-05	26836	0.241595	0
Down	SOBP	4	3.64E-04	186086	0.21967	0
Down	LRRN3	3	5.39E-04	271808	0.247721	0
Down	SCD5	3	1.00E-05	2730	0.208215	0
Down	FILIP1L	2	0	0	1	0
Down	LMTK3	2	0	0	0.199086	0
Down	CHI3L2	2	1.00E-05	1588	0.210047	0
Down	UBL3	2	0	0	1	0

Down	TCEA3	1	0	0	1	0
Down	C5orf15	1	0	0	1	0

Table 7 target gene - miRNA interaction table

Regulation	Target gene	Degree	Top MicroRNA	Regulation	Target gene	Degree	Top MicroRNA
Up	VGF	25	hsa-miR-548p		RYR1	12	hsa-miR-4447
Up	HRK	77	hsa-miR-4505	Down	COL5A1	72	hsa-miR-3175
Up	RRAGD	142	hsa-miR-1297	Down	IL11RA	11	hsa-miR-3941
Up	TMPRSS15	36	hsa-miR-548z	Down	FARP1	98	hsa-miR-7641
Up	IL36B	26	hsa-miR-4522	Down	SPTSSA	107	hsa-miR-429
Up	CNOT7	182	hsa-miR-527	Down	MAMDC2	47	hsa-miR-4420
Up	SYT13	61	hsa-miR-3911	Down	LDB2	55	hsa-miR-8485
Up	AKAP12	97	hsa-miR-302f	Down	IGSF3	179	hsa-miR-3919
Up	IL36B	26	hsa-miR-32-5p	Down	PCSK5	174	hsa-miR-4775
Up	MRPS30	16	hsa-miR-30d-3p	Down	BST2	4	hsa-miR-4469
Up	SLC25A26	15	hsa-miR-622	Down	SLC44A2	46	hsa-miR-4498
Up	RECQL	35	hsa-miR-4426	Down	JMJD8	51	hsa-miR-4492
Up	POMP	25	hsa-miR-2054	Down	GAS6	13	hsa-miR-4267
Up	RPL41	4	hsa-miR-6837-5p	Down	CERCAM	56	hsa-miR-3119
Up	GEM	52	hsa-miR-429	Down	ENG	25	hsa-miR-5787
Up	AP1S1	74	hsa-miR-1321	Down	FERMT2	94	hsa-miR-1279
Up	RPF2	61	hsa-miR-8084	Down	PPFIBP2	9	hsa-miR-4318
Up	MRPL30	54	hsa-miR-548m	Down	MUC1	9	hsa-miR-4283
Up	ELMO1	153	hsa-miR-152-3p	Down	SEPT5	34	hsa-miR-3656
Up	DHRS9	18	hsa-miR-765	Down	COL5A2	128	hsa-miR-5682
Up	ATP6V0D2	33	hsa-miR-451b	Down	PLAT	61	hsa-miR-3943
Up	RBM18	111	hsa-miR-9500	Down	CYP2J2	15	hsa-miR-4303
Up	NPM1	49	hsa-miR-548x-3p	Down	JMJD8	51	hsa-miR-4296
Up	MT1H	5	hsa-miR-1290	Down	TMEM2	143	hsa-miR-4500
Up	RECQL	35	hsa-miR-325	Down	ERBB2	57	hsa-miR-4455
Up	TFPI2	55	hsa-miR-548an	Down	FZD2	72	hsa-miR-4261
Up	THY1	80	hsa-miR-1207-5p	Down	EPSTI1	71	hsa-miR-4493
Up	NDUFA9	11	hsa-miR-7110-3p	Down	COL1A2	80	hsa-miR-4503
Up	CPEB1	158	hsa-miR-548am-3p	Down	HLA-DRB1	11	hsa-miR-6165
Up	SLC25A26	15	hsa-miR-378a-5p	Down	SEPT4	26	hsa-miR-4314
Up	CNOT7	182	hsa-miR-300	Down	HSPB6	80	hsa-miR-4505
Up	GLO1	56	hsa-miR-30d-3p	Down	LDOC1	40	hsa-miR-3148
Up	TTC1	25	hsa-miR-6875-5p	Down	HOXB5	81	hsa-miR-4306
Up	TCP1	19	hsa-miR-185-5p	Down	SCG2	41	hsa-miR-8485
Up	THYN1	2	hsa-miR-7162-3p	Down	CXCL14	77	hsa-miR-4326
Up	RPL14	2	hsa-miR-33a-3p	Down	DICER1	233	hsa-miR-4282
Up	RSL1D1	33	hsa-miR-8080	Down	FHL1	74	hsa-miR-4773
Up	CPEB1	158	hsa-miR-519a-3p	Down	SRPX2	18	hsa-miR-3116
Up	PNLIPRP3	22	hsa-miR-18a-5p	Down	CLPTM1	23	hsa-miR-4441
Up	NDC1	73	hsa-miR-548e-5p	Down	TMEM108	98	hsa-miR-6074
Up	TIGAR	2	hsa-mir-34a-5p	Down	COL6A2	19	hsa-miR-1275
Up	MAGEA1	11	hsa-miR-31-5p	Down	HOXD11	16	hsa-miR-4533
Up	C8orf88	18	hsa-miR-1303	Down	RYR1	12	hsa-miR-4472
Up	NPM1	49	hsa-miR-4520-2-3p	Down	STAT3	113	hsa-miR-4293
Up	EVA1C	14	hsa-miR-211-5p	Down	NCOR2	60	hsa-miR-4532
Up	NMT2	52	hsa-miR-661	Down	PCOLCE2	23	hsa-miR-3686
Up	SPR	29	hsa-miR-421	Down	COL13A1	31	hsa-miR-6086
Up	TPRKB	1	hsa-miR-627-3p	Down	PDGFRA	186	hsa-miR-5695
Up	GRPEL1	25	hsa-miR-22-5p	Down	GAS6	13	hsa-miR-5702
Up	POLR1D	44	hsa-miR-548am-3p	Down	C1QTNF1	35	hsa-miR-4682
Up	GEM	52	hsa-miR-5692a	Down	LCP1	79	hsa-miR-3978
Up	EIF4E	157	hsa-miR-7-1-3p	Down	LRRN3	34	hsa-miR-4310
Up	ANKRD39	13	hsa-miR-4654	Down	FAM43B	36	hsa-miR-5190
Up	RNFT2	95	hsa-miR-922	Down	HLA-DRB4	13	hsa-miR-1286

Up	STIP1	24	hsa-miR-892b	Down	DACT1	83	hsa-miR-1253
Up	CMTM7	22	hsa-miR-1273f	Down	SEMA4B	71	hsa-miR-4430
Up	HOMER2	50	hsa-miR-378j	Down	IGFBP5	129	hsa-miR-4288
Up	LDHB	12	hsa-miR-23c	Down	PDGFRB	83	hsa-miR-5684
Up	GNPTAB	96	hsa-miR-548n	Down	DICER1	233	hsa-miR-4307
Up	MT1M	5	hsa-miR-24-3p	Down	TGFB1I1	20	hsa-miR-6134
Up	TM4SF19	5	hsa-miR-4327	Down	PDPN	73	hsa-miR-5091
Up	GLRX3	21	hsa-miR-940	Down	ZHX3	121	hsa-miR-4464
Up	AP1S1	74	hsa-miR-4500	Down	DPP4	62	hsa-miR-8075
Up	KYNU	15	hsa-miR-200c-3p	Down	COL1A2	80	hsa-miR-5698
Up	TAF15	19	hsa-miR-4999-3p	Down	AKAP13	179	hsa-miR-6088
Up	PLA2G16	6	hsa-miR-581	Down	FSCN1	38	hsa-miR-1912
Up	GCNT3	15	hsa-miR-606	Down	ADM	46	hsa-miR-1297
Up	HMGA1	90	hsa-let-7e-5p	Down	C5orf15	72	hsa-miR-4491
Up	HOMER2	50	hsa-miR-6856-3p	Down	MAGED2	8	hsa-miR-4463
Up	NEMP1	19	hsa-miR-16-2-3p	Down	BST2	4	hsa-miR-6756-5p
Up	PTGES	32	hsa-miR-4419a	Down	PLOD2	56	hsa-miR-3662
Up	KYNU	15	hsa-miR-489-3p	Down	HSD17B6	11	hsa-miR-3646
Up	C10orf128	43	hsa-miR-6887-3p	Down	FILIP1L	46	hsa-miR-3609
Up	PIGP	6	hsa-miR-5197-5p	Down	DAP	44	hsa-miR-3139
Up	CDCA2	9	hsa-miR-888-5p	Down	TNS3	67	hsa-miR-1184
Up	PWP1	9	hsa-miR-548ai	Down	BHMT2	41	hsa-miR-5095
Up	AP1S1	74	hsa-let-7g-5p	Down	GBP2	67	hsa-miR-4660
Up	PCBD1	34	hsa-miR-204-3p	Down	KLHDC2	17	hsa-miR-448
Up	BOLA3	12	hsa-miR-5692c	Down	MFAP4	32	hsa-miR-4419a
Up	GALNT12	29	hsa-miR-548an	Down	ASIC1	182	hsa-miR-608
Up	MRRF	66	hsa-miR-1273g-5p	Down	LAMA4	38	hsa-miR-8061
Up	COQ3	4	hsa-miR-7110-3p	Down	FRMD6	132	hsa-miR-4803
Up	EMG1	3	hsa-miR-4530	Down	TGM2	101	hsa-miR-3155a
Up	EFCAB7	11	hsa-miR-548k	Down	ZFYVE21	33	hsa-miR-892b
Up	RPL34	11	hsa-miR-513c-5p	Down	KDM5B	114	hsa-miR-6124
Up	MAGEC1	8	hsa-miR-6771-3p	Down	SOBP	40	hsa-miR-4642
Up	GAGE2A	4	hsa-miR-30c-2-3p	Down	D2HGDH	3	hsa-miR-4262
Up	TTC26	95	hsa-miR-137	Down	SLC29A4	51	hsa-miR-4455
Up	PSME3	163	hsa-miR-15b-5p	Down	PTPRF	99	hsa-miR-7974
Up	TUBA3E	3	hsa-miR-1185-2-3p	Down	CERCAM	56	hsa-miR-1321
Up	ME2	53	hsa-miR-302d-5p	Down	SHC1	99	hsa-miR-3201
Up	ACAT2	10	hsa-miR-133b	Down	CCDC102A	23	hsa-miR-548aa
Up	TDP1	42	hsa-miR-3609	Down	COL1A1	72	hsa-miR-4510
Up	M6PR	85	hsa-miR-5006-3p	Down	ZHX2	79	hsa-miR-5787
Up	SFR1	43	hsa-miR-93-5p	Down	PRDM1	235	hsa-miR-2113
Up	BOLA3	12	hsa-miR-9500	Down	VASH1	223	hsa-miR-761
Up	MNS1	15	hsa-miR-4252	Down	IGFBP7	22	hsa-miR-4718
Up	NOP16	15	hsa-miR-548ax	Down	PLEKHA1	125	hsa-miR-1827
Up	TCP1	19	hsa-miR-548av-5p	Down	FIBCD1	131	hsa-miR-2861
Up	CD33	66	hsa-miR-6750-3p	Down	CCNG2	158	hsa-miR-3166
Up	CUL4A	51	hsa-miR-548l	Down	DDIT4	70	hsa-miR-548v
Up	LYST	69	hsa-miR-20b-5p	Down	COL6A2	19	hsa-miR-29c-3p
Up	RPS24	34	hsa-miR-616-5p	Down	APLP1	15	hsa-miR-6827-3p
Up	HSPD1	39	hsa-miR-1-3p	Down	HLA-DRB1	11	hsa-miR-939-5p
Up	EEF1B2	8	hsa-mir-548ba	Down	COL8A1	57	hsa-miR-335-3p
Up	NDUFAF4	65	hsa-miR-450a-1-3p	Down	FSTL1	88	hsa-miR-651-3p
Up	ALPL	34	hsa-miR-548s	Down	HOXD10	39	hsa-miR-23c
Up	IL36RN	60	hsa-miR-30c-5p	Down	NTNG1	72	hsa-miR-938
Up	AIM2	3	hsa-miR-4306	Down	EVI5L	41	hsa-miR-449a
Up	YIPF1	15	hsa-miR-1273h-3p	Down	GSTA4	16	hsa-miR-711
Up	AMPH	60	hsa-miR-548az-3p	Down	ATP6AP1	20	hsa-miR-1286
Up	GAGE12D	4	hsa-miR-30c-2-3p	Down	ALPK2	15	hsa-miR-5708
Up	EVI2B	24	hsa-miR-202-3p	Down	MAP4K4	120	hsa-miR-548ak
Up	MGST1	52	hsa-miR-548u	Down	KIFC2	12	hsa-miR-637
Up	MRPL20	11	hsa-miR-548f-3p	Down	SPARC	71	hsa-miR-591
Up	BCKDK	16	hsa-miR-650	Down	LAMA4	38	hsa-miR-590-5p
Up	GAGE12H	1	hsa-mir-335-5p	Down	FAT1	66	hsa-miR-9-3p
Up	TOMM34	38	hsa-miR-7845-5p	Down	CTSC	58	hsa-miR-6077
Up	MCFD2	85	hsa-miR-199a-3p	Down	COL13A1	34	hsa-miR-4477a
Up	ZDHHC12	11	hsa-miR-6715b-5p	Down	LRRC17	49	hsa-miR-6751-3p

Up	PCBD1	34	hsa-miR-204-3p	Down	PAPSS2	62	hsa-miR-548an
Up	TAGLN3	22	hsa-miR-6769b-3p	Down	PYROXD2	6	hsa-miR-4802-5p
Up	OXCT1	70	hsa-miR-22-5p	Down	C1QTNF6	44	hsa-miR-939-5p
Up	ZNF586	47	hsa-miR-4499	Down	MXD4	33	hsa-miR-6890-5p
Up	IL36B	26	hsa-miR-199b-5p	Down	COL16A1	20	hsa-miR-181b-5p
Up	C11orf1	10	hsa-miR-610	Down	HSD17B2	3	hsa-miR-4745-5p
Up	GAGE1	48	hsa-miR-1273h-5p	Down	IL18	13	hsa-miR-1273f
Up	ORC5	67	hsa-miR-1909-3p	Down	FEZ1	28	hsa-miR-4720-3p
Up	CCND3	35	hsa-miR-4516	Down	LOXL1	7	hsa-miR-548s
Up	ACOT7	16	hsa-miR-3650	Down	SGSM2	69	hsa-miR-105-5p
Up	HSPD1	39	hsa-miR-1-3p	Down	UBL3	157	hsa-miR-19b-3p
Up	PPHLN1	108	hsa-miR-3148	Down	NTNG1	72	hsa-miR-643
Up	C12orf49	78	hsa-miR-6745	Down	CXCL12	79	hsa-miR-137
Up	TM4SF19	5	hsa-miR-6894-5p	Down	SAMD14	64	hsa-miR-326
Up	SMAGP	23	hsa-miR-2110	Down	PAM	88	hsa-miR-3976
Up	NTS	33	hsa-miR-95-5p	Down	TGFB3	52	hsa-miR-2278
Up	ARC	61	hsa-miR-19b-3p	Down	CDAN1	24	hsa-miR-4755-3p
Up	GNG11	17	hsa-miR-5584-3p	Down	COL4A5	86	hsa-miR-23c
Up	MRPS17	13	hsa-miR-138-2-3p	Down	GALNT10	125	hsa-miR-532-3p
Up	RNFT2	95	hsa-miR-7977	Down	CERCAM	56	hsa-miR-5189-5p
Up	RPL36A	30	hsa-miR-411-3p	Down	KLF11	104	hsa-miR-20b-5p
Up	CDH11	90	hsa-miR-595	Down	FILIP1L	46	hsa-miR-300
Up	MRPL35	76	hsa-miR-548x-5p	Down	NID2	20	hsa-miR-33a-3p
Up	ALDH3B1	36	hsa-miR-4441	Down	MUC1	9	hsa-miR-5589-5p
Up	MT1F	8	hsa-miR-30c-1-3p	Down	ECM1	20	hsa-miR-4755-3p
Up	DKK1	36	hsa-miR-4503	Down	NFIX	108	hsa-miR-3180
Up	CMSS1	6	hsa-miR-4652-3p	Down	LOX	114	hsa-miR-4303
Up	ETF1	145	hsa-miR-548c-3p	Down	PYGL	9	hsa-miR-548z
Up	SNX8	95	hsa-miR-1976	Down	PLOD2	56	hsa-miR-3166
Up	MND1	18	hsa-miR-3143	Down	BGN	33	hsa-miR-211-3p
Up	PSME3	163	hsa-miR-185-5p	Down	FILIP1L	46	hsa-miR-4665-5p
Up	MGST1	52	hsa-miR-505-3p	Down	HOXC6	41	hsa-miR-5008-5p
Up	TSPAN17	27	hsa-miR-507	Down	SIDT2	76	hsa-miR-3150a-3p
Up	UNG	32	hsa-miR-2392	Down	LGMN	16	hsa-miR-6780b-5p
Up	AGPAT5	96	hsa-miR-382-5p	Down	MARCKS	188	hsa-miR-140-3p
Up	SBSN	1	hsa-miR-3591-5p	Down	CHI3L2	3	hsa-miR-1343-3p
Up	CTSK	49	hsa-miR-4701-5p	Down	MAP4K4	120	hsa-miR-4477b
Up	BATF3	15	hsa-miR-550a-3-5p	Down	FAM46C	62	hsa-miR-3127-3p
Up	SP140	18	hsa-miR-501-3p	Down	QPRT	8	hsa-miR-4659a-3p
Up	PKD1L2	22	hsa-miR-4286	Down	PPIC	52	hsa-miR-340-5p
Up	RPL14	2	hsa-miR-203b-3p	Down	PCYOX1	86	hsa-miR-519c-3p
Up	MAPKAP1	47	hsa-miR-759	Down	MYLK	57	hsa-miR-15b-5p
Up	CSTB	23	hsa-miR-548x-5p	Down	DACT1	83	hsa-let-7f-1-3p
Up	MRPL40	1	hsa-miR-7114-5p	Down	NKX3-2	13	hsa-miR-4789-3p
Up	EXOSC3	29	hsa-miR-1273d	Down	SMARCD3	6	hsa-miR-1227-5p
Up	GTF3C6	1	hsa-miR-4499	Down	IL13RA2	4	hsa-miR-937-5p
Up	RRP15	103	hsa-miR-199a-5p	Down	ZSWIM4	66	hsa-miR-1200
Up	MGST1	52	hsa-miR-6507-5p	Down	ANGPT1	76	hsa-miR-944
Up	COX5B	5	hsa-miR-30c-1-3p	Down	ABCC3	17	hsa-miR-4754
Up	NHP2	13	hsa-miR-3168	Down	LGMN	16	hsa-miR-5571-5p
Up	HSPD1	39	hsa-miR-4710	Down	HNRNPDL	91	hsa-miR-548j-3p
Up	PROCR	16	hsa-miR-129-5p	Down	ANGPT1	76	hsa-miR-4666b
Up	RAC2	30	hsa-miR-4329	Down	GPX8	48	hsa-miR-935
Up	DTNA	182	hsa-miR-4319	Down	C6orf120	145	hsa-miR-548o-5p
Up	NOP16	15	hsa-miR-761	Down	LRP10	30	hsa-miR-3150a-3p
Up	DTNA	182	hsa-miR-320d	Down	DEXI	26	hsa-miR-374a-3p
Up	SLC25A46	51	hsa-miR-1225-5p	Down	AKT1	22	hsa-miR-548h-3p
Up	SPTLC3	73	hsa-miR-586	Down	P4HA1	65	hsa-miR-122-5p
Up	CENPM	5	hsa-miR-7976	Down	TGIF2	109	hsa-miR-34a-5p
Up	SLC25A4	55	hsa-miR-4251	Down	CTSC	58	hsa-miR-425-5p
Up	RPE	80	hsa-miR-642a-3p	Down	AKT1	22	hsa-miR-548bb-3p
Up	TIMM10	15	hsa-miR-665	Down	CADM4	59	hsa-miR-548t-3p
Up	PSMG1	4	hsa-miR-1283	Down	PTGFRN	225	hsa-miR-30c-5p
Up	RPL26L1	4	hsa-miR-5093	Down	IGFBP5	129	hsa-miR-203a-3p
Up	TMEM38B	130	hsa-miR-378g	Down	LAMB2	3	hsa-miR-29b-1-5p

Up	MT1X	6	hsa-miR-376a-3p	Down	PLEKHA1	125	hsa-miR-6715b-5p
Up	RAD51C	6	hsa-miR-3154	Down	HOXC8	36	hsa-miR-567
Up	MT1E	5	hsa-miR-4704-3p	Down	COL6A1	37	hsa-miR-218-2-3p
Up	HEBP1	17	hsa-miR-4328	Down	SPRY1	56	hsa-miR-642b-3p
Up	NDUFB9	2	hsa-miR-2909	Down	LUC7L3	139	hsa-miR-1179
Up	LYPLAL1	52	hsa-miR-496	Down	FAT1	66	hsa-miR-9-3p
				Down	ADSSL1	7	hsa-miR-3677-5p
				Down	COL7A1	23	hsa-miR-3621
				Down	IDUA	11	hsa-miR-4436b-3p
				Down	HLA-B	16	hsa-miR-30e-3p
				Down	BMP1	40	hsa-miR-874-3p
				Down	DENND5A	77	hsa-miR-1976
				Down	RECK	61	hsa-miR-4714-5p
				Down	IGIP	14	hsa-miR-129-5p
				Down	FAM3C	80	hsa-miR-935
				Down	IFITM3	5	hsa-miR-4446-3p
				Down	CBS	18	hsa-miR-4786-3p
				Down	DENND5A	77	hsa-miR-548aw
				Down	IFITM2	4	hsa-miR-3674
				Down	SLC44A4	6	hsa-miR-504-3p
				Down	RRBP1	8	hsa-miR-4508
				Down	COL8A1	57	hsa-miR-6828-5p
				Down	FAM3C	80	hsa-miR-302f
				Down	AKR1D1	55	hsa-miR-4659a-3p
				Down	ZNF541	23	hsa-miR-5047
				Down	SLC26A6	14	hsa-miR-544b
				Down	RNF216	173	hsa-miR-4292
				Down	CTSO	63	hsa-miR-5197-3p
				Down	RHOJ	75	hsa-miR-185-5p
				Down	CCL2	25	hsa-miR-2110
				Down	EPM2AIP1	220	hsa-miR-3139
				Down	PDLIM3	39	hsa-miR-4666a-5p
				Down	SFRP2	53	hsa-miR-1272
				Down	RBM33	117	hsa-miR-548y
				Down	TMEM45A	18	hsa-miR-577
				Down	PCDHB5	14	hsa-miR-421
				Down	FRMD6	132	hsa-miR-641
				Down	TM9SF1	33	hsa-miR-3914
				Down	PIK3IP1	69	hsa-miR-1202
				Down	NPIPB4	3	hsa-miR-3934-3p
				Down	SIPA1L2	90	hsa-miR-107
				Down	CAV2	127	hsa-miR-548ab
				Down	NAV1	153	hsa-miR-449a
				Down	NPEPL1	36	hsa-miR-130a-3p
				Down	PAMR1	14	hsa-miR-4646-3p
				Down	HNRNPA2B1	65	hsa-miR-5692a
				Down	GSN	10	hsa-miR-216b-3p
				Down	ASAP3	34	hsa-miR-143-3p
				Down	ANGPTL2	58	hsa-miR-150-3p
				Down	CNN2	31	hsa-miR-1908-5p
				Down	PLXNA3	48	hsa-miR-92a-2-5p
				Down	PILRB	15	hsa-miR-3936
				Down	PTGFRN	225	hsa-miR-548w
				Down	CELSR3	136	hsa-miR-449c-5p
				Down	RFX8	4	hsa-miR-222-3p
				Down	SLC15A3	1	hsa-miR-4496
				Down	ECM1	20	hsa-miR-4755-3p
				Down	PLAU	33	hsa-miR-634
				Down	FAM20C	21	hsa-miR-5008-5p
				Down	NLRP3	13	hsa-miR-589-3p
				Down	PNISR	63	hsa-miR-107
				Down	COL15A1	60	hsa-miR-5692b
				Down	MAGED2	8	hsa-miR-3121-3p
				Down	PDE4C	39	hsa-miR-4524b-3p
				Down	NREP	88	hsa-let-7f-2-3p
				Down	PRR5L	60	hsa-miR-7977

Down	TPM2	22	hsa-miR-185-5p
Down	RARRES3	3	hsa-miR-6873-5p
Down	ABL1	105	hsa-miR-378g
Down	C21orf58	17	hsa-miR-1203
Down	VLDLR	116	hsa-miR-302e
Down	WDR26	283	hsa-miR-5090
Down	SSPN	161	hsa-miR-378c
Down	SERPINH1	26	hsa-miR-1298-3p
Down	TTC3	51	hsa-miR-105-5p
Down	TCEA3	3	hsa-miR-5000-5p
Down	FUCA1	17	hsa-miR-5680
Down	TNIP1	37	hsa-miR-5194
Down	RECK	61	hsa-miR-219a-5p
Down	GCSAML	54	hsa-miR-936
Down	CASK	164	hsa-miR-16-5p
Down	PSRC1	18	hsa-miR-513a-5p
Down	ULK1	94	hsa-miR-302e
Down	ANG	7	hsa-miR-144-5p
Down	CHPF2	7	hsa-miR-298
Down	PCOLCE	3	hsa-miR-670-3p
Down	LAMA5	10	hsa-miR-363-5p
Down	SVEP1	50	hsa-miR-922
Down	ACTN1	25	hsa-miR-374b-5p
Down	SSTR2	31	hsa-miR-3200-5p
Down	LILRB3	8	hsa-miR-103a-2-5p
Down	STAT3	117	hsa-miR-5692c
Down	CNIH1	95	hsa-miR-548y
Down	TPM1	80	hsa-miR-4499
Down	MCM8	27	hsa-miR-16-1-3p
Down	NUAK1	114	hsa-miR-3689d
Down	H1F0	63	hsa-miR-4477b
Down	FAM19A3	27	hsa-miR-663b
Down	RSRP1	23	hsa-miR-892a
Down	LMCD1	79	hsa-miR-548bb-5p
Down	LMTK3	9	hsa-miR-96-5p
Down	LRRC17	49	hsa-miR-1199-5p
Down	CKB	12	hsa-miR-1972
Down	CLIP3	106	hsa-miR-762
Down	CLIP4	128	hsa-miR-548aw
Down	PHLDB2	71	hsa-miR-135a-5p
Down	NBL1	20	hsa-miR-6785-5p
Down	TCEAL2	2	hsa-miR-556-3p
Down	CPE	22	hsa-miR-431-5p
Down	THRA	75	hsa-miR-6780b-5p
Down	AIRE	9	hsa-miR-181a-2-3p
Down	DACT3	55	hsa-miR-92a-1-5p
Down	CD2	10	hsa-miR-508-5p
Down	IL10	29	hsa-miR-3121-3p
Down	ITGA5	77	hsa-miR-222-5p
Down	C2orf69	145	hsa-miR-371b-5p
Down	THBS2	71	hsa-miR-499a-5p
Down	SCD5	61	hsa-miR-4999-5p
Down	ABLIM1	69	hsa-miR-450a-1-3p
Down	DAPP1	66	hsa-miR-548ba
Down	HSPA2	20	hsa-miR-19b-3p
Down	FAM126B	289	hsa-miR-302c-5p
Down	CMIP	58	hsa-miR-92a-2-5p
Down	GPT2	26	hsa-miR-548g-3p
Down	SLC25A42	108	hsa-miR-4725-3p
Down	PLOD2	56	hsa-miR-520h
Down	PDLIM7	31	hsa-miR-1249-5p
Down	ULK1	94	hsa-miR-17-5p
Down	TAGLN	30	hsa-miR-6808-5p
Down	MAN2A1	180	hsa-miR-32-5p
Down	SLC25A37	38	hsa-miR-892c-3p
Down	TUBG2	16	hsa-miR-4739

Degree – No of miRNA interact with target gene. We taken any one miRNA in table.

Table 8 target gene - miRNA interaction table

Regulation	Target gene	Degree	TFs	Regulation	Target gene	Degree	TFs
Up	RPE	14	CREB1	Down	SRPX2	28	TFAP4
Up	RRP15	8	ZNF146	Down	ANG	22	RCOR2
Up	EXOSC3	18	MXD4	Down	CXCL12	27	SOX13
Up	RPL41	28	PHF8	Down	HOXC8	39	EZH2
Up	MRPL20	35	SMAD5	Down	MAP4K4	13	TEAD3
Up	MCFD2	15	HMG20B	Down	CMIP	23	ELF3
Up	AGPAT5	18	ZNF580	Down	SLC44A4	11	TEAD1
Up	RPL36A	26	MYNN	Down	DEXI	38	NR2F6
Up	SFR1	14	ZNF2	Down	C1QTNF1	18	HMG20A
Up	SLC25A46	18	POLR2H	Down	ADSSL1	32	ZBTB26
Up	SBSN	4	ZNF488	Down	ULK1	17	SOX5
Up	SNHG9	16	DDX20	Down	C6orf120	29	SSRP1
Up	ACAT2	19	ZNF207	Down	DDIT4	30	ZFP64
Up	VTRNA2-1	19	REST	Down	AKT1	20	SMAD4
Up	RPL26L1	24	POLR2A	Down	ABCC3	34	DMAP1
Up	TMEM126A	28	ELK1	Down	BGN	55	KLF9
Up	EXOSC3	28	KDM5B	Down	CAV2	13	ATF3
Up	ZDHHC12	16	ZNF335	Down	D2HGDH	25	HMG20B
Up	TSPAN17	7	EGR2	Down	CELSR3	34	ZNF341
Up	SPR	25	MXD3	Down	ASAP3	44	SP2
Up	VGF	22	INSM2	Down	KIFC2	9	PKNOX1
Up	LYPLAL1	17	NR2C2	Down	ADM	14	NFIA
Up	RPLP0	34	TAF7	Down	CNN2	25	KDM1A
Up	ALDH3B1	15	ETV4	Down	TGIF2	29	THRB
Up	GLRX3	18	ZHX2	Down	FSCN1	25	RARA
Up	TCP1	19	GATA4	Down	KLHDC2	15	FOXA3
Up	MT1F	13	GATAD1	Down	STAT3	18	FOSL2
Up	MRPL45P2	28	SAP30	Down	ECM1	23	MBD1
Up	GNG11	10	RNF2	Down	AKR1D1	32	NR2F1
Up	SNX8	18	ZNF501	Down	CCL2	43	KLF16
Up	ME2	21	BCL11B	Down	HOXB5	25	GLIS1
Up	CENPM	16	BCL11A	Down	LAMA5	27	MLX
Up	NDUFA9	16	GLI4	Down	COL6A1	21	ZNF175
Up	CD33	28	ZBTB11	Down	MAN2A1	35	HBP1
Up	PWP1	10	RCOR2	Down	MEG3	26	RXRB
Up	TTC26	16	NR2F6	Down	HSPB6	45	MXD3
Up	RPL14	9	JUNB	Down	RARRES3	16	ZNF644
Up	TIMM10	2	GATA1	Down	HSD17B2	44	ZNF580
Up	SP140	13	EED	Down	IL11RA	41	SP1
Up	CCND3	14	RARA	Down	ABL1	38	REST
Up	MRPS23	17	ZNF644	Down	CBS	32	CREB3L1
Up	MRPL40	29	ZNF76	Down	LRP10	37	ZNF24
Up	RPS7	5	NFIC	Down	HOXC6	34	BCL11A
Up	ORC5	28	CREB3L1	Down	FAT1	47	ZFX
Up	CSTB	8	ZNF121	Down	GAS6	46	ZNF423
Up	RAD51C	27	ATF1	Down	IDUA	26	KLF13
Up	RPL34	47	TFDP1	Down	IFITM2	35	POLR2A
Up	NHP2	9	MLLT1	Down	HOXD11	45	ZBTB7A
Up	COX5B	12	ETV1	Down	NFIX	33	ZNF2
Up	EMG1	18	NFRKB	Down	H1F0	39	ZNF324
Up	PSME3	12	NCOA1	Down	GSTA4	29	ZNF263
Up	POLR1D	21	ZNF324	Down	KDM5B	26	RFXANK
Up	UNG	11	GATAD2A	Down	ATP6AP1	26	ZNF335
Up	M6PR	28	SP1	Down	SPTSSA	13	ZNF384
Up	DKK1	25	HBP1	Down	ZNF358	16	ZNF121
Up	MRPS31	18	FOSL1	Down	WDR26	20	GFI1B
Up	EIF4E	10	TFAP4	Down	MMP23B	42	ZFP2
Up	ANKRD39	18	KLF16	Down	C21orf58	37	KLF1
Up	RAC2	17	HIC1	Down	LUC7L3	32	SP3
Up	NOP16	28	IRF1	Down	JMJD8	37	WT1
Up	RPS24	12	MYC	Down	LAMA5	4	ZC3H11A
Up	C11orf1	17	RERE	Down	DPP4	23	RERE
Up	WDR12	30	MAZ	Down	SHC1	9	SMC3
Up	RNFT2	14	TFE3	Down	ADM	1	NFE2L2

Up	SNHG7	21	ZBTB33	Down	LOX	27	ZFP37
Up	CUL4A	17	ZNF197	Down	HOXC8	4	UBTF
Up	PIGP	10	SOX5	Down	CKB	36	ZNF610
Up	LOC374443	42	KLF9	Down	FAM20C	38	SIN3A
Up	MT1X	13	ZBTB26	Down	SIPA1L2	12	ZNF558
Up	MT1M	14	SMAD4	Down	THRA	21	BCL6
Up	C12orf49	8	HES1	Down	PDLIM3	18	RNF2
Up	CMTM7	17	DMAP1	Down	IFITM3	17	TRIM28
Up	NPM1	22	CHD1	Down	PLAU	42	BCL11B
Up	MT1E	19	ZFP64	Down	LAMB2	23	ZNF197
Up	HSPD1	10	KDM1A	Down	SPRY1	22	ETV4
Up	CNOT7	11	ZKSCAN1	Down	SLC26A6	38	PHF8
Up	ALDH3B1	14	MLX	Down	ANG	2	CHD4
Up	ANKRD39	18	PPARG	Down	NBL1	23	RAD21
Up	ZDHHC12	7	SREBF2	Down	GALNT10	15	SUZ12
Up	LYPLAL1	10	USF2	Down	NKX3-2	59	TFDP1
Up	SLC25A4	14	ZNF639	Down	HLA-DRB1	16	SUPT5H
Up	SMAGP	16	IRF4	Down	FER1L4	23	FOSL1
Up	GLRX3	13	FOXA3	Down	CLPTM1	38	SAP30
Up	RAC2	12	HDGF	Down	KLF11	40	IRF4
Up	PROCR	9	IKZF1	Down	IL10	31	ID3
Up	MRPS31	16	RXRB	Down	SEPT5	40	EGR1
Up	MRPL45P2	5	FOXK2	Down	ZFYVE21	42	ZBTB33
Up	SNX8	16	FOXM1	Down	PLXNA3	27	CCNT2
Up	THYN1	8	YY1	Down	ZHX2	18	PPARG
Up	PIGP	8	HDAC6	Down	TPM1	50	GLIS2
Up	STIP1	14	HMGN3	Down	TUBG2	21	GATA4
Up	RPS7	7	ZNF7	Down	RYR1	41	HIC1
Up	TFPI2	8	HDAC2	Down	APLP1	33	CTCF
Up	M6PR	10	TARDBP	Down	TMEM45A	22	GTF2F1
Up	COX5B	20	WRNIP1	Down	RBM33	23	ZNF639
Up	EMG1	11	MTA1	Down	C5orf15	47	IRF1
Up	TSPAN17	21	RFXANK	Down	PCYOX1	17	GATAD2A
Up	RSL1D1	5	NR2F2	Down	PYGL	9	MYBL2
Up	RPLP0	21	KLF11	Down	SLC25A42	9	MBD4
Up	ARC	15	GLIS2	Down	AKR1D1	9	HNF4G
Up	GALNT12	16	EGR1	Down	FAM46C	41	NRF1
Up	CTSK	6	PRDM10	Down	SEMA4B	38	HMGN3
Up	LDHB	25	SIN3A	Down	CNN2	23	NFIL3
Up	EEF1B2	10	NFIA	Down	QPRT	22	DRAP1
Up	ZDHHC12	14	CCNT2	Down	MYL5	64	MAZ
Up	SPINK1	3	HHEX	Down	NREP	26	INSM2
Up	RPS7	9	ATF4	Down	CERCAM	40	FOXM1
Up	CNOT7	2	TBX3	Down	COL6A2	32	KLF8
Up	NOP16	13	THRB	Down	MAP4K4	18	YY1
Up	UNG	5	THAP1	Down	PDLIM7	22	CHD1
Up	NHP2	8	MBD2	Down	MEG3	23	EGR2
Up	TCP1	14	HDAC1	Down	CHI3L2	5	RUNX3
Up	MRPS31	14	DPF2	Down	C1QTNF6	31	ARID4B
Up	VGF	9	KLF4	Down	LCP1	5	SPI1
Up	RPL26L1	10	ZNF623	Down	UBL3	15	ZNF382
Up	VTRNA1-3	6	EHMT2	Down	PHLDB2	7	ATF4
Up	BATF3	17	ZBTB7A	Down	HSD17B2	14	CREB1
Up	RPS24	15	ZFP37	Down	CHPF2	24	ELK1
Up	LOC374443	14	KLF7	Down	NPEPL1	12	MTA1
Up	MT1M	11	CTBP2	Down	SGSM2	33	GATAD1
Up	NDUFB9	13	CTCF	Down	FEZ1	29	ZNF501
Up	RPL41	21	ZFX	Down	SEPT4	20	KLF4
Up	THY1	15	EZH2	Down	JMJD8	11	PRDM2
Up	C11orf1	12	TRIM22	Down	COL4A5	43	ZNF76
Up	RAD51C	16	ZNF394	Down	DACT3	29	ZEB1
Up	RPS24	20	ZEB1	Down	ASIC1	12	ZNF146
Up	EXOSC3	16	GLIS1	Down	SOBP	28	KLF7
Up	NMT2	11	SP3	Down	GAS6	29	SP7
Up	ARC	20	ZFP2	Down	LMCD1	36	ATF1
Up	HMGA1	16	ZNF423	Down	GSTA4	2	POLR3A
Up	BATF3	5	STAT3	Down	KDM5B	6	RCOR1
Up	SNHG9	6	CREM	Down	ANG	1	BDP1
Up	RPLP0	5	SCRT2	Down	ANG	1	BRF1
Up	CDCA2	10	ZNF18	Down	LRRN3	16	CBFB

Up	LYPLAL1	7	EBF1	Down	PCOLCE	13	ZNF101
Up	PIGP	14	ZNF71	Down	LILRB3	19	ZKSCAN1
Up	SBSN	7	PRDM2	Down	CELSR3	15	ETS1
Up	PKD1L2	4	CEBPB	Down	HLA-B	17	WRNIP1
Up	RPE	17	CEBPG	Down	SERPINH1	16	ARNT
Up	MRPL45P2	21	HCFC1	Down	TM9SF1	25	ZNF394
Up	MRPL20	17	SSRP1	Down	FUCA1	20	CEBPG
Up	MT1H	26	ETS1	Down	THRA	21	USF2
Up	NOP16	7	NFIL3	Down	SIDT2	41	ELF1
Up	COQ3	10	ELF3	Down	COL16A1	14	NR2F2
Up	CSTB	15	KLF8	Down	ZSWIM4	13	TRIM24
Up	ZDHHC12	16	ESRRA	Down	TGFB1I1	36	ZNF71
Up	DKK1	15	MBD1	Down	LRP10	23	ETV1
Up	EVI2B	9	SP7	Down	CMIP	20	KLF6
Up	CNOT7	9	L3MBTL2	Down	IFITM3	5	TBP
Up	PPHLN1	10	BCL6	Down	CXCL12	8	HDAC2
Up	HOMER2	14	ZNF341	Down	TTC3	8	ZNF7
Up	MCFD2	14	TGIF2	Down	CELSR3	16	GMEB2
Up	NOP16	15	GMEB2	Down	MEG3	25	NFYC
Up	GLO1	20	E2F5	Down	COL1A1	14	THAP1
Up	MRPL40	20	SMARCA5	Down	COL5A1	6	ZNF217
Up	RAD51C	21	ZNF610	Down	MARCKS	7	TARDBP
Up	GTF3C6	6	RFX3	Down	HOXB5	7	ZNF641
Up	RPS24	7	CDC5L	Down	PSRC1	7	ZNF407
Up	MRPL35	7	TRIM24	Down	ANG	1	GTF3C2
Up	C11orf1	1	NFATC1	Down	EPM2AIP1	8	SREBF2
Up	TTC26	4	DNMT1	Down	LUC7L3	10	CUX1
Up	EVI2B	14	HMBOX1	Down	ANG	1	HNF4A
Up	STIP1	6	MNT	Down	LGMN	29	L3MBTL2
Up	CCL26	3	PTRF	Down	MARCKS	26	HDAC1
Up	TPRKB	37	ELF1	Down	KLHDC2	30	IKZF1
Up	RPL41	17	KLF13	Down	SLC15A3	8	TBX21
Up	OXCT1	25	KLF1	Down	DDIT4	5	MAFK
Up	MRPS17	29	KDM5A	Down	COL6A1	22	ADNP
Up	GTF3C6	29	GTF2E2	Down	MFAP4	27	PRDM10
Up	NDUFB9	14	MEF2D	Down	SSTR2	8	HDAC6
Up	RNFT2	9	DIDO1	Down	PDGFRA	10	BHLHE40
Up	DTNA	15	NRF1	Down	GPX8	17	ZNF366
Up	CENPM	41	ZNF24	Down	THBS2	2	POU5F1
Up	ORC5	15	KLF6	Down	PLAT	16	ZBTB17
Up	TEAD3	7	TEAD3	Down	KDM5B	2	EHMT2
Up	C11orf1	1	MTA3	Down	FAM19A3	34	MYNN
Up	SNX8	8	ZNF407	Down	C5orf15	7	ZNF16
Up	VTRNA1-3	9	GFI1B	Down	KLF11	6	E2F6
Up	MRPL20	9	RAD21	Down	VASH1	6	ZNF264
Up	POLR1D	17	GTF2F1	Down	CKB	15	ZBTB1
Up	PIGP	7	ZNF239	Down	MAN2A1	19	FOXJ2
Up	SPR	16	NR2F1	Down	ASAP3	3	PTRF
Up	KYNU	9	HMG20A	Down	ENG	29	NR4A1
Up	ANKRD39	7	ZBTB17	Down	DEXI	7	ZNF584
Up	EFCAB7	3	CTBP1	Down	SLC25A42	20	ZNF589
Up	NHP2	15	ZNF263	Down	H1F0	13	NCOA1
Up	NPM1	18	NCOR1	Down	RARRES3	14	GTF2A2
Up	TOMM34	11	ADNP	Down	PNISR	21	TAF7
Up	RPS24	13	LEF1	Down	IDUA	36	SMAD5
Up	MRPL40	14	BCOR	Down	FSCN1	22	NCOR1
Up	MT1E	11	GATA2	Down	CTSO	21	TRIM22
Up	PIGP	11	ZBTB40	Down	ASIC1	5	CEBPD
Up	GALNT12	17	ID3	Down	HSPA2	14	PBX2
Up	RPS7	10	ARNT	Down	TEAD4	5	TEAD4
Up	PKD1L2	10	CREB3	Down	FOXA2	5	FOXA2
Up	CCND3	10	MIXL1	Down	DENND5A	12	HES1
Up	MRPS23	4	EP300	Down	KLF11	7	MITF
Up	DHRS9	8	FOXA2	Down	C1QTNF6	10	RFX1
Up	RPL41	6	PKNOX1	Down	IFITM2	24	ESRRA
Up	LYPLAL1	12	TRIM28	Down	LUC7L3	12	KDM5A
Up	GLO1	6	MAFK	Down	ECM1	30	NR2C2
Up	HMGA1	14	ZNF589	Down	FZD2	20	CTBP2
Up	MRPL40	8	CEBPD	Down	HLA-DRB6	23	ZNF18
Up	CSTB	3	TEAD2	Down	PLEKHA1	12	ZNF83

Up	PSMG1	13	JUND	Down	IL11RA	31	SMARCA5
Up	RRP15	8	TEAD4	Down	BST2	4	HDAC8
Up	RPS24	10	BACH1	Down	THRA	8	IRF2
Up	MCFD2	12	ARID4B	Down	TM9SF1	6	STAT1
Up	RAD51C	5	ETV6	Down	LRRN3	11	JUNB
Up	GLRX3	16	SMARCE1	Down	GSN	31	E2F5
Up	MRPL45P2	12	NR4A1	Down	MUC1	28	GTF2E2
Up	NPM1	8	WT1	Down	PLAT	8	JUND
Up	CDCA2	2	CHD2	Down	SHC1	17	NFRKB
Up	RAC2	11	STAT1	Down	WDR26	11	POLR2H
Up	TSPAN17	11	SP2	Down	MAP4K4	16	CREM
Up	RPL36A	6	ZNF384	Down	C1QTNF1	5	SETDB1
Up	CENPM	8	ZNF143	Down	SPTSSA	7	SIN3B
Up	SP140	4	MTA2	Down	TTC3	17	GLI4
Up	COQ3	25	GABPA	Down	KIFC2	7	MNT
Up	TTC26	4	TAL1	Down	LMCD1	2	ESR1
Up	SBSN	6	ZNF558	Down	NPEPL1	10	MBD2
Up	RPS7	14	FOXJ2	Down	C21orf58	3	E2F4
Up	MT1H	7	TBP	Down	ZSWIM4	14	DDX20
Up	MT1F	16	ATF3	Down	JMJD8	9	ZNF239
Up	MRPL39	12	GTF2A2	Down	CADM4	13	ZNF623
Up	NDUFAF4	16	CUX1	Down	STAT3	16	EED
Up	GRPEL1	15	MAX	Down	LGMN	14	HCFC1
Up	TTC26	3	RELA	Down	PCYOX1	3	RBBP5
Up	RPL14	7	MITF	Down	MUC1	5	DEK
Up	CUL4A	5	ZNF547	Down	MCM8	5	RFX3
Up	CHD1	1	ZNF707	Down	MAN2A1	12	ZNF143
Up	SMAGP	4	PRDM1	Down	QPRT	12	EBF1
Up	RPLP0	6	TSHZ1	Down	NFIX	9	CDC5L
Up	STIP1	6	RAD51	Down	TGIF2	18	TSHZ1
Up	EEF1B2	6	CEBPA	Down	CADM4	2	ZNF140
Up	DKK1	3	ZNF217	Down	CKB	5	ZNF8
Up	SNHG7	4	GATA3	Down	RYR1	9	SCRT1
Up	MT1IP	11	TEAD1	Down	SPTSSA	5	ATF2
Up	MRPS23	11	SIN3B	Down	CADM4	1	ZNF292
Up	SFR1	8	DRAP1	Down	EPM2AIP1	3	THRAP3
Up	HMGA1	13	NFYC	Down	COL6A1	8	ZNF512
Up	MRPS17	12	SOX13	Down	LOC155060	20	ZBTB40
Up	NOP16	10	IRF2	Down	CBS	4	ZNF585B
Up	RPL26L1	6	GTF2B	Down	SPDYE7P	22	BCOR
Up	M6PR	7	ZNF175	Down	LRP10	22	LEF1
Up	ME2	15	ZNF101	Down	MXD4	19	HDGF
Up	RAC2	9	TBX21	Down	RBM33	2	JUN
Up	MRPL20	11	PML	Down	PLAT	16	MEF2D
Up	SNHG9	5	ZNF366	Down	TM9SF1	7	MXI1
Up	ORC5	7	DEK	Down	CHI3L2	17	ZNF207
Up	MRPL39	15	CBFB	Down	SEPT5	2	MEF2A
Up	POMP	5	RBBP5	Down	IL10	4	POU2F2
Up	PTGES	8	NFE2L2	Down	LGMN	16	MLLT1
Up	GALNT12	7	ZNF584	Down	CHI3L2	1	MYB
Up	POLR1D	11	FOSL2	Down	CHI3L2	1	BCL3
Up	RPS7	6	BHLHE40	Down	SEPT5	2	MEF2C
Up	MRPL40	2	MAFG	Down	NLRP3	7	NFIC
Up	ZDHHC12	7	ZNF382	Down	GALNT10	35	ZBTB11
Up	GRPEL1	3	E2F6	Down	PDLIM7	16	SIRT6
Up	SYT13	5	RFX1	Down	PCYOX1	16	SMARCE1
Up	MRPL20	9	ZNF512	Down	DDIT4	5	DIDO1
Up	RPL41	9	THRAP3	Down	SLC25A37	9	MIXL1
Up	MRPS30	4	ZHX1	Down	ECM1	16	SMARCA4
Up	TTC26	8	ZNF83	Down	CHPF2	3	SREBF1
Up	HMGA1	9	NFE2	Down	FAM46C	9	BACH1
Up	RPS7	3	RUNX1	Down	PYGL	15	CREB3
Up	RPL36A	5	SMARCA4	Down	ZSWIM4	10	ARID1B
Up	MRPL45P2	8	ARID1B	Down	FAT1	29	TFE3
Up	RPLP0	5	SIRT6	Down	IGSF3	14	GATA2
Up	SP140	6	TCF7	Down	IFITM3	13	RELA
Up	PNLIPRP3	4	FOS	Down	CMIP	1	ETV6
Up	MRPL20	8	MXI1	Down	CNN2	4	NFYB
Up	LYPLAL1	9	PBX2	Down	HSPB6	2	SP4
Up	MRPL39	2	BCL3	Down	CNN2	1	AEBP2

Up	SNX8	6	SUPT5H	Down	MAP4K4	10	CEBPB
Up	LYPLAL1	1	PRDM12	Down	H1F0	9	USF1
Up	MRPL20	2	MCM7	Down	KIFC2	11	RAD51
Up	NTS	4	SCRT1	Down	COL5A1	2	NFYA
Up	TDP1	2	E2F4	Down	HOXD10	2	ZNF202
Up	MRPL39	2	TCF3	Down	COL6A1	1	NONO
Up	RAC2	7	POU2F2	Down	LAMA4	8	SCRT2
Up	MRPL40	2	STAT5A	Down	LCP1	8	TCF7
Up	STIP1	3	SREBF1	Down	IDUA	14	GABPA
Up	MRPL40	1	TCF7L2	Down	TUBG2	6	NFE2
Up	MRPL40	1	SRF	Down	KLF11	8	MAX
Up	MRPL40	1	TBL1XR1	Down	DDIT4	5	GATA1
Up	MRPL40	1	ETS2	Down	MAN2A1	6	TAL1
Up	MRPL40	1	TSC22D4	Down	DDIT4	1	ZFP41
Up	RPL14	5	HDAC8	Down	RARRES3	2	PTTG1
Up	STIP1	2	USF1	Down	FSCN1	3	RUNX1
Up	STIP1	2	TAF1	Down	DDIT4	2	ETS2
Up	MRPS23	1	ZNF140	Down	TGM2	5	EP300
Up	MT1F	8	HNF4G	Down	RARRES3	2	PYGO2
Up	MT1H	8	MYBL2	Down	PIK3IP1	6	MAFF
Up	MT1IP	8	MBD4	Down	EPM2AIP1	7	HMBOX1
Up	RBM18	6	SUZ12	Down	DDIT4	2	TEAD2
Up	NHP2	1	SIX4	Down	SEPT5	2	DNMT1
Up	RPL41	2	GATAD2B	Down	HLA-DRB1	5	PML
Up	ZDHHC12	3	RCOR1	Down	SPTSSA	4	HHEX
Up	RPL36A	2	ZNF264	Down	SERPINH1	12	DPF2
Up	NOP16	2	SIX5	Down	SPRY1	7	TBX3
Up	NPM1	1	ZNF202	Down	ERBB2	2	FOXK2
Up	PIGP	3	ZBTB1	Down	EVI5L	6	CEBPA
Up	RPLP0	2	JUN	Down	FAM46C	2	TCF3
Up	PROCR	1	YBX1	Down	SLC25A37	2	STAT5A
Up	RAD51C	2	BATF	Down	PCDHB5	7	GATA3
Up	RPL41	2	ATF2	Down	TGIF2	4	MTA2
Up	SP140	3	SPI1	Down	FER1L4	4	ZNF547
Up	RPL36A	3	ZNF641	Down	HLA-DRB1	1	RFX5
Up	SP140	3	RUNX3	Down	HOXC8	8	CTBP1
Up	RPL41	1	KAT2B	Down	HOXB5	2	ZHX1
Up	RPL41	1	KAT2A	Down	HOXC6	3	ZNF488
Up	SFR1	1	SMC3	Down	PAM	2	CBX8
Up	TAF15	2	MAFF	Down	HSPB6	1	CEBPZ
Up	SNHG7	1	MYB	Down	HSPB6	1	SIX5
Up	SNHG9	1	IRF3	Down	HSPB6	1	KAT2B
Up	SNHG9	1	BRCA1	Down	SHC1	2	IRF3
Up	SPINK1	2	CBX8	Down	IFITM3	1	STAT2
Up	TTC26	1	PYGO2	Down	SIPA1L2	2	FOS
Up	VGF	1	SP4	Down	KDM5B	1	TAF1
Up	VGF	1	NANOG	Down	ZHX2	3	MYC
Up	VTRNA1-3	1	POLR3A	Down	KLHDC2	1	HSF1
Up	VTRNA1-3	1	BDP1	Down	KLHDC2	1	PPARGC1A
Up	VTRNA1-3	1	NFE2L1	Down	LRP10	2	YBX1
Up	VTRNA1-3	1	BRF1	Down	MUC1	2	GTF2B
				Down	NAV1	1	MTA3
				Down	NAV1	1	NFATC1
				Down	NPEPL1	2	SIX4
				Down	PDGFRA	1	CHD7
				Down	PNISR	3	TCF7L2
				Down	SEPT4	1	GATAD2B
				Down	TGM2	2	MCM7
				Down	SEPT5	1	TSC22D4
				Down	SEPT5	1	MCM3
				Down	SPTSSA	1	PLRG1

Degree – No of target gene interact with TF. We taken any one target gene in table. TF transcription factors
